# *In Vitro* Generation of Neuromesodermal Progenitors Reveals Distinct Roles for Wnt Signalling in the Specification of Spinal Cord and Paraxial Mesoderm Identity

**DOI:** 10.1371/journal.pbio.1001937

**Published:** 2014-08-26

**Authors:** Mina Gouti, Anestis Tsakiridis, Filip J. Wymeersch, Yali Huang, Jens Kleinjung, Valerie Wilson, James Briscoe

**Affiliations:** 1 MRC-National Institute for Medical Research, London, United Kingdom; 2 MRC Centre for Regenerative Medicine, Institute for Stem Cell Research, School of Biological Sciences, University of Edinburgh, Edinburgh, United Kingdom; Stanford University School of Medicine, Howard Hughes Medical Institute, United States of America

## Abstract

Cells of the spinal cord and somites arise from shared, dual-fated precursors, located towards the posterior of the elongating embryo. Here we show that these neuromesodermal progenitors (NMPs) can readily be generated *in vitro* from mouse and human pluripotent stem cells by activating Wnt and Fgf signalling, timed to emulate *in vivo* development. Similar to NMPs *in vivo*, these cells co-express the neural factor Sox2 and the mesodermal factor Brachyury and differentiate into neural and paraxial mesoderm *in vitro* and *in vivo*. The neural cells produced by NMPs have spinal cord but not anterior neural identity and can differentiate into spinal cord motor neurons. This is consistent with the shared origin of spinal cord and somites and the distinct ontogeny of the anterior and posterior nervous system. Systematic analysis of the transcriptome during differentiation identifies the molecular correlates of each of the cell identities and the routes by which they are obtained. Moreover, we take advantage of the system to provide evidence that Brachyury represses neural differentiation and that signals from mesoderm are not necessary to induce the posterior identity of spinal cord cells. This indicates that the mesoderm inducing and posteriorising functions of Wnt signalling represent two molecularly separate activities. Together the data illustrate how reverse engineering normal developmental mechanisms allows the differentiation of specific cell types *in vitro* and the analysis of previous difficult to access aspects of embryo development.

## Abstract 2

Timed pulses of WNT and FGF signaling convert human and mouse pluripotent stem cells into neuromesodermal progenitors that can be directed to differentiate into spinal cord and paraxial mesoderm cells

## Author Summary

Stem cells are providing insight into embryo development and offering new approaches to clinical and therapeutic research. In part this progress arises from “directed differentiation” – artificially controlling the types of cells produced from stem cells. Here we describe the directed differentiation of mouse and human pluripotent stem cells into cells of the spinal cord and paraxial mesoderm (the tissue that generates muscle and bone that is normally found adjacent to the spinal cord). During embryo development, spinal cord and paraxial mesoderm arise from a shared group of precursors known as neuromesodermal progenitors (NMPs). We show that signals to which NMPs are exposed in embryos can be used to generate NMPs from pluripotent stem cells in a dish. We define conditions for the conversion of these NMPs into either spinal cord or mesoderm cells. Using these conditions, we provide evidence that the decision between spinal cord and mesoderm involves a gene, *Brachyury*, that promotes mesoderm production by inhibiting spinal cord generation. Together the data illustrate how mimicking normal embryonic development allows the generation of specific cell types from stem cells and that this can be used to analyse cells that are otherwise difficult to study.

## Introduction

The differentiation of embryonic stem cells (ESCs) to specific cell types offers insight into developmental mechanisms and has potential therapeutic applications. For example the differentiation of neural progenitors (NPCs) from monolayers of ESCs seeded in serum free conditions is a model of neural induction and regional patterning [1]. In the absence of additional signals, NPCs differentiated from ESCs adopt an anterior-dorsal neural (telencephalon) identity [1],[2]. The addition of Sonic Hedgehog (Shh) ventralises these neural progenitors, mimicking the *in vivo* role of Shh [3],[4]. Exposing NPCs to retinoic acid (RA) results in the repression of anterior identity and the induction of genes that typify hindbrain and anterior spinal cord (cervical) identity [5]. This has been taken as support for the idea that newly generated NPCs are by default anterior and are then posteriorised by exposure to specific extrinsic signals [6],[7]. It is notable, however, that RA is actively excluded in the progenitors of the posterior spinal cord after gastrulation [8] and that commonly used ESC differentiation protocols do not efficiently generate neural cells of the more posterior spinal cord such as thoracic and lumbar spinal cord cells marked by posterior Hox gene expression, including Hoxc8–10 expression [9].

The anterior and posterior nervous system has distinct origins [10]–[12]. Anterior epiblast expresses *Otx2* and contributes cells to the anterior nervous system [2],[13] whereas spinal cord progenitors are located posteriorly [14]–[16]. Clonal analysis indicates that the spinal cord shares a common lineage, at least in part, with the trunk paraxial mesoderm that forms the somites [15]. The dual-fated neuromesodermal precursors (NMPs) of these tissues are located in the node-streak border (NSB), caudal lateral epiblast (CLE) cell layer adjacent to the regressing node and the chordoneural hinge of the tail bud [13],[14],[17],[18]. Cells in these regions coexpress the neural marker Sox2 and nascent mesoderm marker Brachyury [8],[19],[20]. Genetic lineage tracing experiments confirm that many spinal cord cells previously expressed Brachyury [21] indicating that as cells from regions harbouring NMPs move into the neural tube they downregulate *Brachyury* but maintain *Sox2* expression and consolidate neural identity. By contrast, NMPs that enter the primitive streak delaminate basally, downregulate *Sox2* and acquire expression of the paraxial mesoderm marker *Tbx6* [22] en route to somite formation. Strikingly, in embryos lacking *Tbx6*, paraxial mesoderm cells express Sox2 and transdifferentiate into neural cells, providing additional support for the inter-relationship between spinal cord and somitic mesoderm [22]–[24]. As yet, however, the existence of NMPs has only been revealed *in vivo* and the inaccessibility of this population makes them difficult to study.

The region occupied by NMPs is exposed to Wnt and Fgf ligands [16]. These signals are required for body axis elongation [16] and both Wnt and Fgf signalling have been implicated in mesoderm and neural induction [22],[25]–[31]. *In vivo* and *in vitro* evidence has suggested that Wnt signalling is responsible for posteriorising tissue by inducing posterior Hox genes [29],[32],[33]. Together, the data suggest that the generation of posterior neural tissue and paraxial mesoderm proceeds by Wnt and Fgf signalling inducing a neuromesodermal bipotential intermediate. To test this idea, we developed an efficient *in vitro* differentiation method for spinal cord and paraxial mesoderm from mouse and human pluripotent stem cells. We show that carefully timed and calibrated pulses of Wnt and Fgf signalling generate a population of cells that transiently coexpress Sox2 and Brachyury in which the expression of posterior Hox genes are induced. Transcriptome analysis is consistent with the equivalence of these cells to the NMPs found *in vivo*. *In vivo* grafting and directed *in vitro* differentiation confirm the ability of NMPs to assume spinal cord or paraxial mesoderm cell fates. We further show that Brachyury is not required for the production of posterior neural cells or for the induction of posterior Hox genes, hence separating the posteriorising and mesoderm inducing functions of Wnt signalling. Taken together the data define a means to generate posterior neural and paraxial mesodermal tissues *in vitro* and illustrate how the directed differentiation of stem cells provides novel insight into developmental mechanism.

## Results

### Generation of Neural Progenitor Cells with Spinal Cord Identity

**Figure 1:**
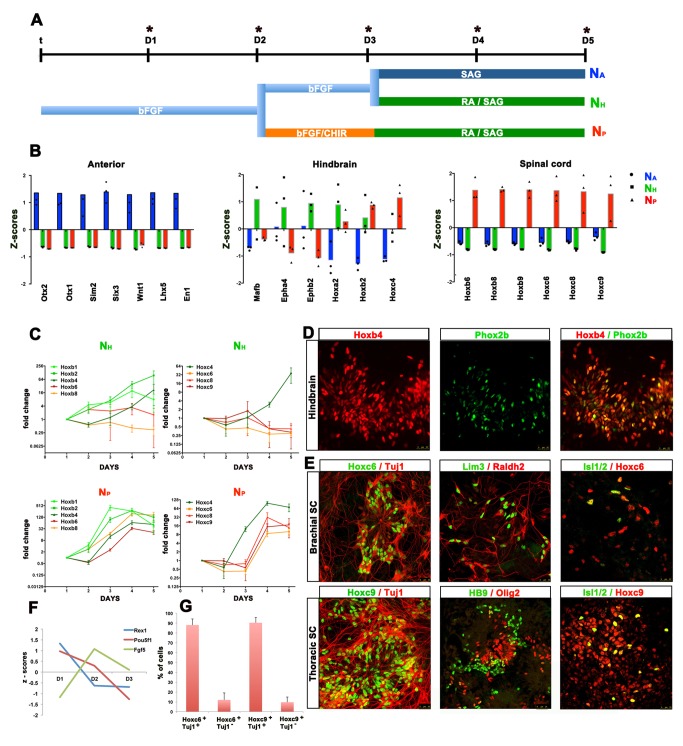
Generation of neural cells with specific AP identities from ESCs. (A) Schematic representation of differentiation conditions used for the generation of NPCs with specific Anterior (N_A_), Hindbrain (N_H_) and Spinal cord (N_P_) identities. (B) Relative expression levels of the indicated genes from N_A_, N_H_ and N_P_ cells at day 5 (D5) of differentiation indicate that N_A_, N_H_ and N_P_ cells express distinct sets of genes. The standard scores (z-scores) of the indicated genes from mRNA-seq analysis reveals that N_A_ cells express high levels of forebrain markers including *Otx1* and *Otx2*; N_H_ cells express genes characteristic of hindbrain including *Mafb* and *Hoxa2* genes; N_P_ cells express high levels of posterior 5′ Hox genes including *Hoxc8* and *Hoxc9*. The individual Z-score for each replicate is indicated on the graph with circles, triangles and squares. (C) Time course of Hoxb and Hoxc cluster activation in cells cultured in N_H_ and N_P_ conditions showing fold change compared to D1. Posterior Hox genes are selectively activated only in the N_P_ conditions and show temporal colinearity with the induction of anterior Hox genes prior to posterior Hox genes [74]. In N_H_ cells *Hoxb1* and *Hoxb2* are induced prior to *Hoxc4*. However, the more posterior Hox genes are not induced. By contrast, in N_P_ conditions the 5′ Hox genes *Hoxc6*, *Hoxc8* and *Hoxc9* are induced at D4 and their expression is maintained at day 5. (Note log_2_ scale). (D) Immunohistochemistry indicates that N_H_ cells analysed at D8 differentiate into MNs of hindbrain identity coexpressing Hoxb4 and Phox2b. (E) N_P_ cells exposed to SAG generate spinal neurons coexpressing Hoxc6 and Hoxc9 with b-tubulin (Tuj1). These were not detected in N_H_ conditions. Coexpression of Hoxc6 and Hoxc9 with Islet1 indicates the generation of spinal MNs of forelimb and thoracic identity, respectively. These MNs also expressed Lim3/Raldh2 and HB9. (F) Graph showing the standard scores (z-scores) of Zfp42 (Rex1), Pou5F1 (Oct3/4) and Fgf5 from the mRNA-seq from D1 to D3. The kinetics of gene expression indicate that ESCs progressively lose their stem cell identity and acquire a transient epiblast identity at D2. (G) Hoxc6/Tuj1 and Hoxc9/Tuj1 positive cells were quantified in independent fields of D8 cells differentiated in N_P_ conditions. All data used to generate the plots of Figure 1 can be found in Data S1.

To identify conditions for the generation of posterior neural cells from monolayers of mouse ES cells (mESCs), we cultured mESCs in serum free media containing bFgf for 3 days (D1–D3) and then transferred these to media lacking bFgf for an additional 2 days [1] (Figure 1A). This resulted in the induction of a post-implantation epiblast-like intermediate by D2, indicated by the downregulation of the “naïve” pluripotency marker Zfp42 (Rex1) and the upregulation of the epiblast marker Fgf5 (Figure 1F) [34]. At this stage, Pou5f1, which is expressed in both mESCs and epiblast-like cells, is maintained (Figure 1F) [34]. In all experiments a Shh agonist, SAG, was added at D3 in order to generate a predictable ventralised identity for subsequent comparisons. The transcriptome of cells was then analysed at D5 by mRNA-seq. Consistent with previous studies [35]–[39], cells in these conditions had acquired an anterior neural identity (N_A_), exemplified by the expression of *Otx1* and *Otx2* [2]. The presence of SAG induced the expression of ventral neural markers (Figure S1A). Addition of retinoic acid (RA) and SAG to differentiating mESCs at D3 downregulated anterior neural markers (e.g. *Otx2, Six3, Lhx5*) and instead genes typical of hindbrain identity, including *Hoxa2*, *Hoxb2*, *Mafb*, *Epha4* and *Ephb2* were expressed (Figure 1B) [40]. However markers of spinal regions of the neural tube, such as the 5′ Hox genes *Hoxc6*, *Hoxc8* and *Hoxc9* were not detected (Figure 1B) [5],[9]. Changing the timing or concentration of RA used in these experiments did not result in the efficient induction of more posterior spinal cord identity [29].

To recapitulate the sequence of signalling events that generate the spinal cord, we seeded mESCs into serum free media containing bFgf. At D2, Wnt signalling was induced by the addition of the Wnt agonist CHIR99021 (CHIR). bFgf and Wnt agonist were removed at D3 and cells exposed to media containing RA and SAG until D5. Examination of gene expression profiles indicated that cells subjected to the FGF/CHIR/RA regime expressed genes characteristic of the spinal cord including high levels of 5′ Hox genes *Hoxb6, Hoxb8, Hoxc6, Hoxc8, Hoxc9* and low levels of the anterior neural and brainstem markers *Otx2* and *Mafb* (Figure 1B). Together, the data suggested that a brief pulse of Wnt signalling between D2–D3 was sufficient to posteriorise differentiating mESCs. We termed the neural cells generated in this regime N_P_ cells and cells that display anterior and brainstem identity N_A_ and N_H_, respectively (Figure 1A).

We confirmed the posteriorisation and neural identity of N_p_ cells using qRT-PCR and immunostaining (Figure S1C–D). Analysis of the time course of Hox gene expression in N_H_ and N_P_ cells indicated that their temporal sequence of induction matched the *in vivo* time course [40]: *Hoxb1* was induced within 12 h of exposure to Wnt signalling, whereas more 5′ Hox genes were induced later (Figure 1C). Notably the more posterior Hox genes, e.g, *Hoxc6* and *Hoxc9* were not induced in N_H_ cells. In N_p_ cells *Hoxc6*, *Hoxc8* and *Hoxc9* were strongly induced at D4 (Figure 1C). Delaying the addition of CHIR to differentiating mESCs until D3 resulted in a concomitant shift in the timing of Hox gene induction (Figure S2A–C). Furthermore, in agreement with studies indicating that RA represses the most posterior Hox genes [41], exposure of cells to FGF/CHIR without subsequent addition of RA induced *Hoxc10* characteristic of the lumbar spinal cord (Figure S2E). Finally we passaged N_H_ and N_P_ cells at D5 and allowed them to differentiate until D8, at which point we assayed the expression of genes expressed in motor neurons (MNs). Both N_H_ and N_P_ cells adopted a neuronal morphology and expressed the neuronal marker class III β-tubulin (Tuj1). The N_H_ cells acquired a posterior hindbrain MN identity evident by the coexpression of Hoxb4 and the cranial motor neuron marker Phox2b [42] (Figure 1D). In the case of N_P_ cells however, only a few Hoxb4 expressing cells were detected (Figure S1D) and most of the β-tubulin expressing neurons acquired a Hoxc6 and Hoxc9 identity characteristic of neurons of the brachial and thoracic spinal cord, respectively [43] (Figure 1E,G). Moreover N_P_ cells expressed Olig2, a marker of somatic motor neuron progenitors, as well as the differentiated MN markers Hlxb9 and Islet1/2 [3] (Figure 1E). Taken together these data indicate that similar to the situation *in vivo* [44] and in embryoid bodies [29] exposure of monolayers of differentiating ESCs to a combination of Wnt, Fgf and RA signalling generates spinal cord cells.

### Generation of N_P_ Cells Proceeds via Neuromesodermal Progenitors

**Figure 2:**
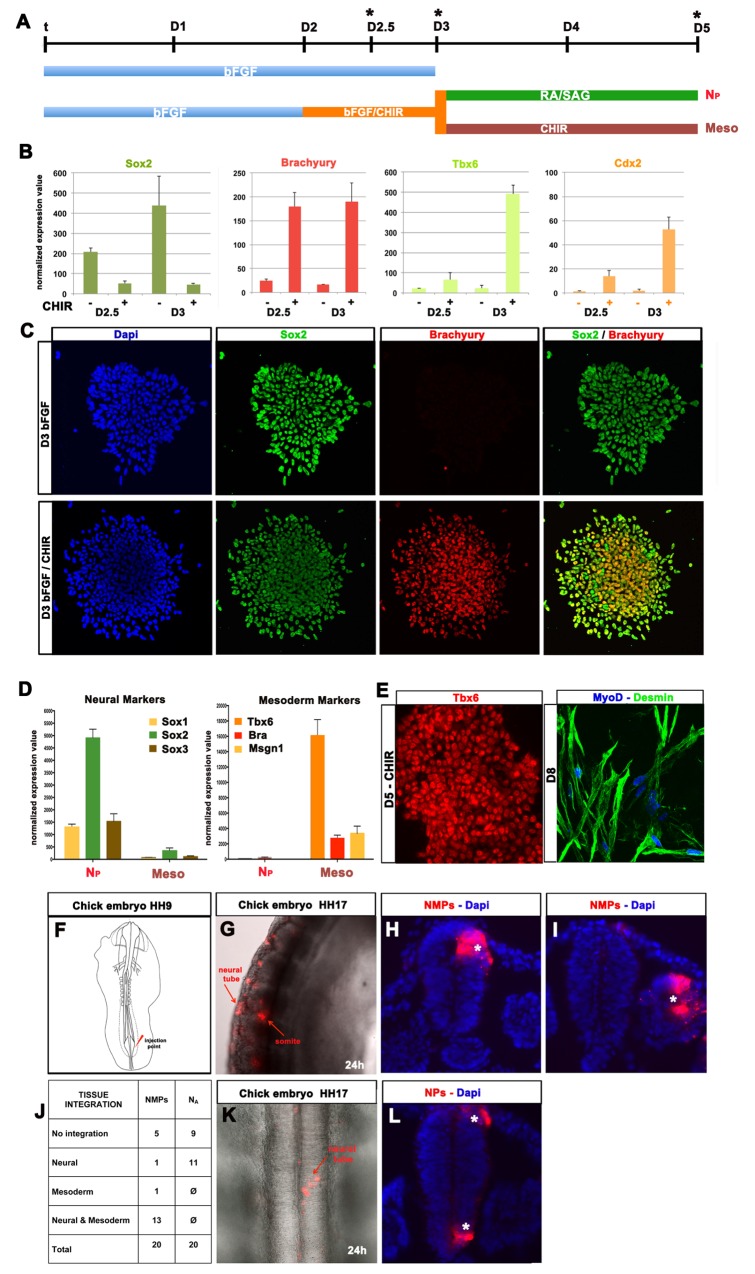
Transient Wnt and FGF signalling induce dual fated neuromesodermal progenitors. (A) Schematic of differentiation protocols used to generate mesoderm and neural cells from a common NM progenitor population. (B) mRNA-seq expression values of *Sox2*, *Brachyury*, *Tbx6* and *Cdx2* following exposure to bFGF alone or bFGF/CHIR for 12 h (D2.5) and 24 h (D3). Activation of Wnt signalling with CHIR upregulated Brachyury within 12 h. Expression of *Tbx6* and *Cdx2* was also upregulated in NMPs by D3, whereas *Sox2* transcript levels were decreased. (C) Immunostaining of cells treated with FGF/Wnt revealed the coexpression of Brachyury with Sox2 (NMPs). In the absence of Wnt, NPCs express Sox2 but the expression of Brachyury is only evident in a very small proportion of cells. (D) mRNA expression values of neural (*Sox1*, *Sox2*, *Sox3*) and mesodermal progenitors markers (*Tbx6*, *Bra*, *Msgn1*) in posterior neural (N_P_) and mesodermal cells (Meso) at D5 show the generation of distinct populations depending on treatment after D3. Removal of Wnt at D3 results in the generation of neural cells expressing Sox1–3 whereas continued Wnt exposure induces expression of Tbx6, Brachyury and Msgn1, characteristic of paraxial mesodermal. (E) Immunostaining indicates that continued Wnt exposure generates paraxial mesodermal progenitors that express Tbx6 at D5 and Desmin and MyoD at D8. (F) Sketch of a chick embryo (HH8–9) showing the injection site (IS) of NMP or N_A_ cells. (G) NMP cells were labelled with DiI and transplanted in the CLE region. After 24 h the cells had incorporated into both the neural tube and somites. Whole-mount and transverse sections of HH17 chick embryos show the incorporation (asterisks) in the neural tube (H) and somites (I). (J) Table summarizing the number of chick embryos that were injected at stage HH8–9 and had engrafted cells in the neural tube, the somites or both 24 h later. Injection of N_A_ cells resulted in incorporation only in the neural tube (K, L). All data used to generate the plots of Figure 2 can be found in Data S2.

To address how the combination of Wnt and Fgf signalling induces spinal cord identity we examined gene expression in differentiating ESCs at D2.5 and D3 (Figure 2A). ESCs that had been exposed to Fgf/Wnt signalling for 12 h (D2.5) and 24 h (D3) induced the expression of *Cdx2* and the mesoderm transcription factors *Brachyury* and *Tbx6* (Figure 2B). Recombinant Wnt3a protein had a similar activity to CHIR in these assays (Figure S2F). By contrast, ESCs cultured in the absence of Wnt agonist, expressed significantly lower levels of these genes (Figure 2B). These data suggest that Wnt signalling, in combination with Fgf, is initiating a mesodermal transcriptional program. This is consistent with the loss of mesoderm in mouse embryos lacking Wnt3a [45] and the induction of *Brachyury* by β-catenin [28].

It was also noticeable that the levels of *Sox2* mRNA were transiently reduced in D2.5 and D3 N_P_ cells treated with FGF/CHIR (Figure 2B). We therefore assayed Sox2 and Brachyury proteins by immunostaining in D3 N_P_ and N_A_ cells. Strikingly, the level of Sox2 protein was similar in N_P_ and N_A_ cells, consistent with the long half-life of Sox2 protein [46]. Moreover, ∼80% of N_P_ cells coexpressed Brachyury and Sox2 (Figure 2C) whereas only a small number of N_A_ cells expressed Brachyury. These data suggest that the exposure to bFgf and Wnt signalling induces a cell identity reminiscent of the dual-fated neuromesodermal progenitors present during axial elongation in the CLE [15],[44] (Figure S4E).

If D2–D3 N_P_ cells represent NMPs, they should form mesoderm. To test this, we transferred cells at D3 into media containing Wnt agonist but lacking bFgf. In these conditions (termed Meso) the expression of *Sox1*, *Sox2* and *Brachyury* were downregulated and several genes characteristic of paraxial mesoderm, including *Tbx6* and *Msgn1* [47], were significantly upregulated (Figure 2D). Immunostaining revealed that more than 90% of cells in this condition expressed Tbx6 protein at D5 (Figure 2E). By D8 Desmin, the intermediate filament protein of muscle sarcomeres [48] and the muscle transcription factor MyoD were highly expressed (Figure 2E). Thus the continued exposure of cells to Wnt signalling induces a paraxial mesodermal identity that differentiates to a muscle-like identity. This provides evidence that ESCs exposed to Wnt and bFgf at D2–D3 represent bipotential neuromesodermal cells that can differentiate into either mesoderm or neural tissue.

We next tested the *in vivo* potential of NMP cells. For these experiments we took advantage of the chick. Cells with NMP-like behaviour have been identified in chick [16] and chick embryos provide an accessible and experimentally tractable vertebrate host for grafts of mouse ESCs [49]. We grafted small groups of DiI labelled D3 N_A_ cells, not exposed to Wnt signalling, or D3 NMPs, exposed to Fgf/Wnt signalling for 24 h, into the caudal lateral epiblast of Hamburger-Hamilton (HH) stage 8–9 chick embryos (Figure 2F). Analysis of embryos 24 h later revealed efficient incorporation and migration of the NMP cells to both the neural tube and the somites (Figure 2G). Transplanted cells from a single graft contributed to multiple anterior-posterior levels and most embryos showed contribution to both spinal cord and somites (Figure 2G–J). In several embryos grafted cells were also observed in the tail bud of the embryo as well as the neural tube and somites (Figure 2J). Contribution to endoderm was not observed. By contrast, transplanted N_A_ cells showed somewhat lower rates of engraftment and contributed only to the neural tube and not to somites (Figure 2K–L). These data confirm the bipotency of the *in vitro derived* NMP cells and demonstrate that similar to *in* vivo NMPs [15] [44] they contribute to both neural and paraxial mesoderm lineage. Single cell and clonal analysis, *in vivo* and *in vitro*, will be necessary to test the potency of individual cells and to understand the molecular mechanism by which neural and/or mesodermal progeny are generated from NMP.

### A Distinct Transcriptional Programme Identifies Neuromesodermal Progenitors

**Figure 3:**
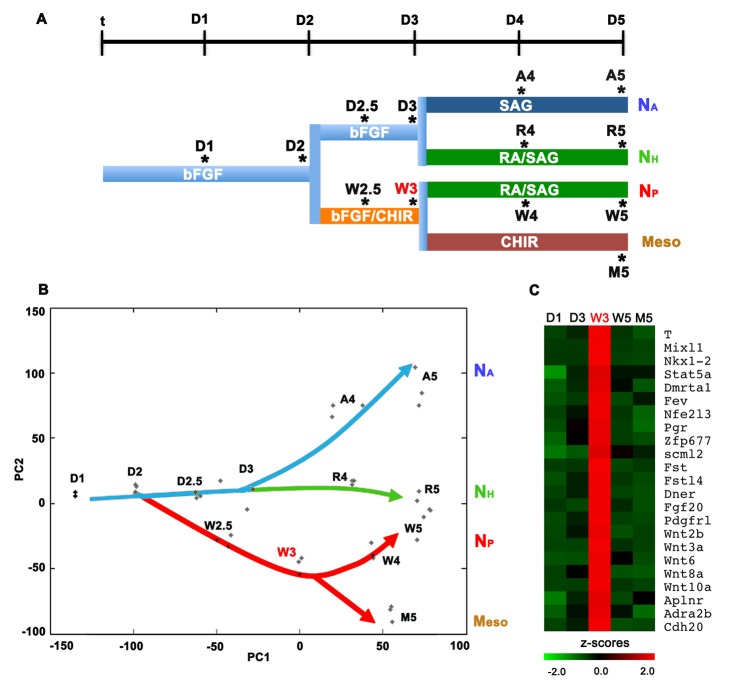
Induction of a distinct transcriptional programme in neuromesodermal progenitors. (A) Schematic of the differentiation conditions used for transcriptome analyses. Three independent samples of RNA were collected at the indicated time points from each of the conditions and analysed by mRNA-seq. (B) Principal component analysis of the triplicate samples at each time point of differentiation shows the distinct trajectories for each of the developmental pathways. The first principal component (PC1) appears to represent the time of differentiation whereas PC2 represents the treatment regime. Note that PC3 separates N_H_ and N_P_ at D5. Anterior neural (N_A_) and hindbrain neural (N_H_) appear to share a common trajectory, whereas posterior neural (N_P_) appear to share a common trajectory with paraxial mesodermal cells (Meso). (C) Heatmap of expression levels of a subset of the genes significantly and uniquely upregulated in NMPs compared to other samples. A complete list of genes uniquely upregulated in NMPs is summarized in Table S1. The data used to generate the heatmap can be found in Data S3.

We took advantage of the *in vitro* differentiation to analyse the transcriptional programmes that generate each of the neural and mesodermal lineages (Figure 3A). Principal component analysis of the transcriptomes indicated that each differentiation pathway could be clearly distinguished (Figure 3B). Strikingly, the first principal component (PC) appeared to represent developmental time and the second PC the tissue identity of the differentiated cells. The data revealed a set of genes that distinguished N_P_, N_A_, N_H_ cells and Meso cells (Figure S3 and Tables S2, S3). These included the upregulation of *Mafb* and *Phox2b* in N_H_ samples and the upregulation of posterior Hox genes, notably *Hoxc6*, *Hoxc8* and *Hoxc9* in N_P_ samples. By contrast, the induction of genes such as *Tbx6*, *Hes7* and *Hoxc8* and *Hoxc9* in D5 cells subjected to mesodermal conditions confirmed the posterior paraxial identity of these cells. Moreover, the analysis indicated a bifurcation in the transcriptional programmes that generate anterior neural and brainstem cells from those that produce posterior neural and paraxial mesodermal cells. It was notable that gene expression typical of paraxial mesoderm was evident at D4 of N_P_ differentiation suggesting a gradual separation of neural and mesodermal identity. Together these data provide a molecular correlate to the distinct cellular origins of anterior and posterior neural tissue [15] and identifies the NMP state as the branch point in the developmental trajectories.

We identified genes upregulated in NMPs compared to mESCs at D1 and N_A_ cells at D3. Comparing these to genes induced in D5 neural and mesodermal cells revealed a large intersection. Thus, in part, NMPs have a transcriptional programme that is a combination of neural and mesodermal gene expression. In addition however, a set of ∼240 genes appeared uniquely upregulated in NMP cells (Table S1). These included the transcription factors *Brachyury*, *Nkx1.2* (also known as *Sax1*), which is expressed in the stem zone of midgestation embryos [50],[51] *Mixl1* [52], *Wnt3a* and *Cdx2* which are expressed in the primitive streak and nascent mesoderm [32],[33]. In addition *Follistatin*, which plays a key role in neural induction by blocking TGFβ signalling [53] and components of the Fgf signalling pathway, which is implicated in mesoderm induction [16], are upregulated in NMPs (Figure 3C). Together these data support the idea that exposure of differentiating ESCs to Fgf/Wnt signalling between D2 and D3 induces a bipotential neuromesodermal population equivalent to that found *in vivo* in the CLE [15],[16],[18],[22] and that the balance and timing of these two signals influences the further differentiation of these cells into neural or mesodermal tissues.

### Generation of NMPs from Mouse Epiblast Stem Cells

**Figure 4:**
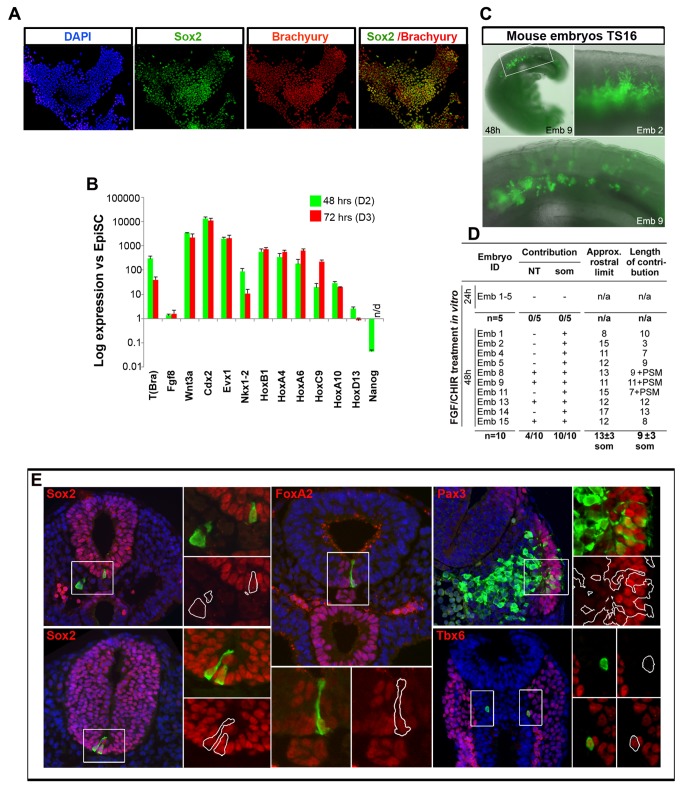
Generation of NMPs from EpiSCs. (A) Brachyury/Sox2 immunocytochemistry in EpiSC cultures treated with FGF/CHIR for 72 h. (B) qPCR analysis for indicated markers in mouse EpiSCs treated with FGF/CHIR. Error bars = s.d. (n = 3). n/d, not determined. Results are represented as log_10_ ratio of expression versus untreated EpiSCs. The data used to generate the plot can be found in Data S4. (C) Combined fluorescence/brightfield microscopy showing donor cell incorporation of grafted GFP^+^ EpiSC differentiated for 48 h in FGF/CHIR after 48 h embryo culture. (D) Table summarizing the incorporation of grafted GFP^+^ EpiSC differentiated for 24 h or 48 h in Fgf/Wnt within host embryos. NT, neural tube; Som, somite; PSM, presomitic mesoderm; n/a, not applicable. (E) Representative examples of donor cell incorporation (green, GFP) and differentiation (red, immunofluorescence for indicated markers). Cell nuclei were stained with DAPI (blue). White boxes indicate the position of magnified images of GFP^+^ cells.

The activation of Wnt signalling in differentiating mouse epiblast stem cells (EpiSCs) leads to a modest induction of Brachyury/Sox2 coexpressing cells, suggestive of NMP identity [19]. To improve the efficiency of this induction we adapted our mESC protocol to take account of the more advanced developmental state of EpiSC compared to mESCs (Figure 1F). Accordingly, we exposed EpiSCs to a range of CHIR (Wnt) and bFgf concentrations and assayed the expression of Sox2 and Brachyury (Figure S4A). Maximal proportions of Sox2/Brachyury coexpressing cells resulted from 3 µM CHIR and 20 ng/ml bFgf (hereafter referred to as FGF/CHIR) (Figure 4A, Figure S4A).

Assaying a broader panel of genes supported the idea that FGF/CHIR was inducing NMP identity. The expression of the pluripotency factor Nanog was undetectable and the majority of the Sox2 expressing cells expressed minimal levels of Oct4, suggesting that they had exited pluripotency (Figure 4B, Figure S4D). Moreover, the acquisition of Brachyury/Sox2 coexpression coincided with an upregulation of *Wnt3a*, *Cdx2* and *Nkx1.2* as well as trunk *Hox* genes (Figure 4B), characteristic of embryo and mESC derived NMPs. Consistent with this, the paraxial/somitic mesoderm markers *Tbx6* and *Meox1* and the neural factor *Sox1* were expressed in these conditions (Figure S5A). Immunostaining indicated that by D3 of differentiation Tbx6 and Sox2 expression were mutually exclusive (Figure S5B). By contrast the expression of genes characteristic of anterior neural plate (e.g. *Otx2* and *Six3*) and endoderm (*Foxa2*) [54] were largely absent in FGF/CHIR conditions (Figure S5A). Collectively, these data indicate that, similar to mESCs, stimulation of Wnt and Fgf signalling in mouse EpiSCs leads to the induction of an NMP state.

The developmental potential of differentiated mouse EpiSCs has previously been tested by transplantation into mouse embryos [19],[55]. We therefore grafted EpiSC-derived NMPs constitutively expressing GFP into the NSB of E8.5 embryos. After 48 h in culture, we observed extensive incorporation of GFP expressing cells (15/15 embryos) (Figure 4C–D). Sections from these embryos revealed integration of transplanted cells into the somites and presomitic mesoderm of host embryos (10/10) and neural tube (4/10) (Figure 4D). We did not observe contributions to endoderm or other tissues. Antibody staining for paraxial mesoderm (Tbx6), somite/dermomyotome (Pax3), neural (Sox2) and floor plate (Foxa2) markers confirmed that the engrafted cells had acquired the marker expression of their host environment (Figure 4E). Moreover, examination of the rostral limit of labelling using the somite level as a reference revealed that grafted EpiSC derived NMPs behaved similarly to homotopic grafts of microdissected E8.5 NSB cells [56]. Strikingly, few cells grafted into the node of E7.5 embryos showed any incorporation (2 out of 8 embryos had 8–10 incorporated cells/embryo), suggesting that these conditions produce a population incompatible with gastrulation-stage development. Similarly, cells differentiated for 24 h in FGF/CHIR did not incorporate into the NSB of E8.5 embryos (n = 5) (Figure 4D). Collectively, these results suggest that 48 h treatment of EpiSCs with FGF/CHIR results in coexpression of Brachyury/Sox2 (up to 90%, Figure S4B) and generates NMPs that functionally resemble their *in vivo* counterparts.

### Directed Differentiation of Human ES Cells to NMPs

**Figure 5:**
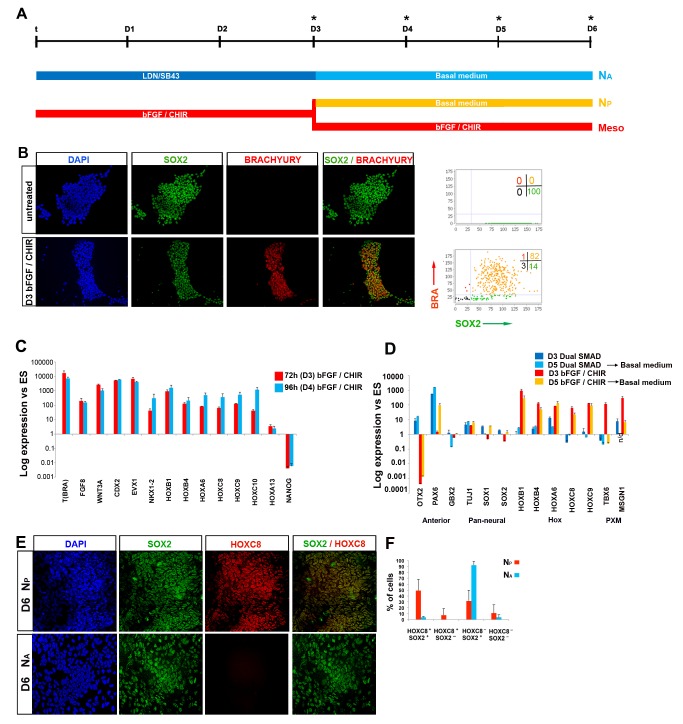
Generation and characterisation of hNMPs. (A) Scheme describing the culture conditions employed for neural differentiation of hES cells treated for 72 h either with FGF/CHIR or subjected to dual SMAD inhibition (LDN, LDN193189; SB43, SB431542). (B) BRACHYURY/SOX2 immunocytochemistry in undifferentiated and FGF/CHIR-treated (48 h) hES cells. Corresponding graphs depict image analysis of BRACHYURY and SOX2 expression in the indicated culture conditions. Numbers: percentages of cells in each quadrant. (C) qPCR analysis for indicated markers in hES cells treated with FGF/CHIR for 72 h (D3) or 96 h (D4). Error bars = s.d. (n = 2). Results are represented as log_10_ ratio of expression versus untreated hES cells. (D) qPCR analysis for indicated differentiation markers in hES cells differentiated in N2B27 following either an NM progenitor induction- (N_P_) or a dual SMAD inhibition-intermediate step (N_A_). Error bars = s.d. (n = 2). Anterior, anterior neural markers; PXM, paraxial mesoderm; n/d, not determined. (E) Immunocytochemistry for SOX2/HOXC8 in N_A_ and N_P_ culture conditions indicated in the scheme (A). (F) Quantitation of the coexpression of Hoxc8 with Sox2 in N_A_ and N_P_ conditions. All data used to generate the plots can be found in Data S5.

The resemblance of mouse EpiSCs to human embryonic stem cells (hESCs) prompted us to ask whether an analogous FGF/CHIR treatment regimen was sufficient to generate human NMPs. Treatment of three independent hESC lines with CHIR and bFgf from D0–D3 downregulated *NANOG* and OCT4 and upregulated the suite of NMP expressed genes—*BRACHYURY*, *NKX1.2* and *CDX2*—similar to mouse ESCs and EpiSCs (Figure 5C, Figure S4D). SOX2 expression was maintained in this population and up to ∼80% of cells co-expressed SOX2 and BRACHYURY (Figure 5B, Figure S4B). We also observed the spontaneous upregulation of paraxial mesoderm/somite markers (TBX6, *MSGN1, MEOX1*). (Figure S5C). By contrast, the expression of a lateral plate (*KDR*) and an endoderm (*FOXA2*) marker were minimal (Figure S5C). Thus FGF/CHIR treated hESCs appear to adopt an NMP identity and are likely to represent the *in vitro* correlates of the SOX2 and BRACHYURY co-expressing cells found in the caudal epiblast of human embryos [8]. Consequently we dubbed these cells hNMPs.

To test the potency of hNMPs, we treated hESCs with FGF/CHIR for 72 h to drive the generation of BRACHYURY^+^/SOX2^+^ cells and then re-plated them for a further 48 h in serum free media without additional factors to promote the induction of spinal cord identity (Figure 5A). We termed these cells N_P_ and compared them to neural cells derived from hESCs using a dual SMAD inhibition protocol involving Nodal and BMP inhibitors (SB/LDN) [57]. Both conditions induced neural identity, exemplified by increased levels of *SOX2*, *TUBB3* and *PAX6* (Figure 5D). As expected, neural cells generated using dual SMAD inhibition expressed the anterior marker *OTX2* but lacked expression of HOX genes (Figure 5D). By contrast, neural cells derived from NMPs expressed *SOX1* and the posterior HOX genes *HOXC6*, *HOXC8* and *HOXC9* but not *OTX2* (Figure 5D). A similar expression profile was obtained after treatment with RA and dual Shh agonists SAG and purmorphamine (Pur). This also induced expression of the motor neuron progenitor marker *OLIG2* (Figure S5D). Antibody staining verified HOXC8 expression in N_P_ conditions and revealed that the majority of HOXC8^+^ cells co-expressed SOX2, confirming their neural identity (Figure 5E, F). Treatment of neural cells for 48 h with FGF/CHIR following 72 h dual SMAD inhibition did not result in HOXC8 induction suggesting that posteriorisation is necessary before or concomitant with neural induction (Figure S5E). Together these data suggest that neural differentiation of hNMPs generates spinal cord progenitors similar to mNMPs.

We next tested whether hNMPs differentiate into mesoderm by culturing them in the presence of CHIR alone. This resulted in the expression of paraxial/somitic mesoderm markers *TBX6*, *MSGN1* and *MEOX1* (Figure S5D), but little if any expression of *KDR*, a lateral plate mesoderm marker (Figure S5D). Taken together these findings provide evidence of a human NMP population that gives rise to spinal cord and paraxial mesoderm derivatives but not anterior neurectoderm or lateral plate mesoderm. Moreover, a similar set of developmental cues induces and directs NMPs in human and mouse, consistent with a similar ontogeny of trunk tissues in these species.

### Brachyury Is Required for the Induction of Mesoderm but Not Posterior Neural Identity

The ability to generate NMPs *in vitro* allows experimental investigations of trunk development that are challenging or impossible *in vivo*. For example, although the requirement for *Brachyury* in mesoderm formation is well-established [58]–[60], the truncation of embryos lacking Brachyury has complicated analysis of its role in the elaboration of spinal cord identity. In zebrafish, a non-autonomous role for *Brachyury* orthologues has been identified [60]. It is unclear whether in mammals Brachyury is required directly to maintain NMPs and therefore generate spinal tissue or indirectly via Wnt induction to establish a mesodermal niche that signals to generate or maintain posterior neural tissue. To address this we took advantage of Brachyury null mESCs (BTBR10) derived from embryos lacking Brachyury [61].

**Figure 6:**
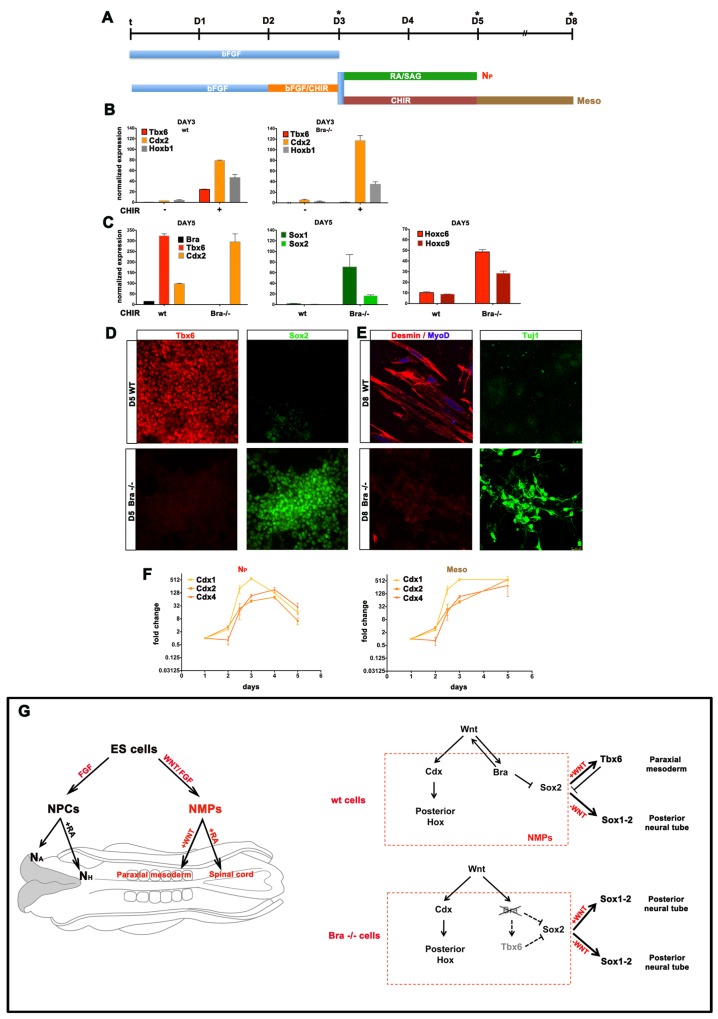
Brachyury is necessary for mesoderm formation but not posterior neural identity. (A) Schematic of the conditions used for mesoderm differentiation. (B) qRT-PCR analysis of the expression of *Tbx6*, *Cdx2* and *Hoxb1* relative to b-actin at D3 of differentiation in wild-type (wt) and Brachyury null cells (Bra^−/−^) with and without CHIR. In wild-type cells activation of Wnt signalling induces the expression of these three genes. In the absence of Brachyury while *Cdx2* and *Hoxb1* continue to be induced by Wnt signalling, Tbx6 induction is lost. (C) qRT-PCR analysis of the expression of mesodermal, neural and posterior marker genes at D5 of differentiation in wt and Bra^−/−^ ESCs exposed to CHIR from D2–D5 (Meso conditions). Posterior Hox genes *Hoxc8* and *Hoxc9* are induced in both wt and Brachyury null cells. However, in contrast to wild-type cells neural markers *Sox1* and *Sox2* are expressed only in Bra^−/−^ cells exposed to Meso conditions. (D) Immunostaining of Tbx6 and Sox2 at D5 of Meso differentiation in Bra^−/−^ and wild-type ESCs. Wild-type cells efficiently differentiate to paraxial mesoderm and expresses Tbx6 but not Sox2. By contrast Bra^−/−^ cells differentiate to a neural identity exemplified by Sox2 expression in the absence of Tbx6. (E) At D8 wt cells cultured in CHIR express Desmin/MyoD but not β-Tubulin (Tuj1) whereas Bra^−/−^ cells fail to produce Desmin/MyoD and differentiate into neurons expressing β-Tubulin (Tuj1). (F) The time course of Cdx gene expression in posterior neural (N_P_) and mesodermal inducing conditions (Meso). Cdx genes are transiently induced in posterior neural cells but continuously upregulated in mesodermal cells. (Note, log_2+_ scale). All data used for the plots can be found in Data S6. (G) Model for the generation of spinal cord and paraxial mesodermal tissue from ESCs. ESCs cultured in N2B27 with FGF generate anterior but not posterior neural tissue. The activation of Wnt signalling in differentiating ESCs results in the generation of a bipotential neuromesodermal progenitor, equivalent to those found in the CLE of the embryo, which generate spinal cord or paraxial mesodermal tissue. Wnt signalling activates homeodomain proteins of the Cdx family in these progenitors that could account for the posteriorisation. In addition, Wnt signalling activates the mesodermal specifier Brachyury (Bra) that is required for Tbx6 induction and the repression of Sox2. The induction of Brachyury induces the Brachyury-Wnt autoregulatory loop that is necessary for mesoderm induction. In the absence of this gene ESCs differentiate into posterior neural tissue even in the presence of continued Wnt signalling.

Assaying Brachyury null cells at D3 of differentiation indicated that, in contrast to wild-type ESCs, Tbx6 expression was not upregulated by exposure to FGF/CHIR signalling, whereas *Cdx2* and *Hoxb1* expression were induced (Figure 6B). This is consistent with the lack of posterior mesoderm induction in Brachyury mutant embryos and prompted us to address the fate of Brachyury mutant cells that would normally form mesoderm. In wild type cells exposed to Meso conditions, Tbx6 was highly expressed at D5 (Figure 6D), as were Desmin and MyoD at D8 (Figure 6E). By contrast Brachyury null cells subjected to the same conditions failed to differentiate into paraxial mesoderm as indicated by the absence of Tbx6 (Figure 6D). Instead these cells expressed *Sox1*, *Sox2* and posterior Hox genes (*Hoxc6* and *Hoxc9*) at D5 (Figure 6C) and differentiated into β-Tubulin expressing neurons (Figure 6E). These data indicate that within mouse NMPs, Brachyury not only specifies mesodermal identity via mechanism(s) in addition to its direct stimulation of Wnt signalling, but also represses neural identity. In the absence of Brachyury, NMPs adopt a neural differentiation route. Thus the induction of posterior neural tissue is not dependent on Brachyury. Moreover the data separate the mesoderm inducing and posteriorising activity of Wnt signalling and provide evidence that posteriorisation of the CNS is not dependent on mesoderm derived signals.

What could be responsible for the induction of posterior Hox genes? Analysis of the transcriptome data revealed the induction in NMPs of the Cdx genes *Cdx1*, *2* and *4*, which have been implicated in the regulation of Hox gene expression (Figure 6F) [29],[62]. Induction of both *Cdx1* and *Cdx2* were detectable within 12 h of FGF/CHIR exposure and the levels of all three genes increased further at D3 and D4 of N_P_ differentiation and at D5 of Meso differentiation. Moreover, the induction of *Cdx2* by Fgf/Wnt signalling was maintained in Brachyury null ESCs (Figure 6B). Thus the induction of Cdx proteins by Fgf/Wnt signalling represents a good candidate for the posteriorisation of NMPs. Moreover the temporal accumulation of Cdx levels following Wnt exposure might provide a timing mechanism for the progressive induction of increasingly more posterior Hox genes.

## Discussion

We describe the *in vitro* generation of bipotential neuromesodermal progenitors from both mouse and human pluripotent stem cells that are capable of producing posterior neural tissue and paraxial mesodermal tissue. This recapitulates the behaviour of NMPs residing in the CLE and NSB [15],[22] (Figure 6G). Moreover, we provide evidence that Wnt signalling has two distinct functions in NMPs, initiating a mesodermal differentiation programme by regulating Brachyury expression and independently posteriorising these cells. It is also likely that Brachyury maintains NMPs during axis elongation by forming a positive feedback loop with Wnt gene expression as has been previously shown [60]. Strikingly, a neuromesodermal precursor is also present in ascidian embryos [63]. Similar to vertebrates, the induction of these cells depends on the timing of Wnt and Fgf signalling [64],[65]. Moreover the mesoderm and posterior nervous system of many arthropods, including short germband insects, arises from a shared progenitor population that is exposed to Wingless signalling and expresses Cdx [66]. Thus molecular and cellular features of the development of the neural and mesodermal components of the trunk appear to be evolutionarily conserved across bilaterian embryos. This emphasizes the distinct developmental origins of cells that form anterior and posterior regions of bilaterian embryos, suggesting an explanation as to why it has proved difficult to generate spinal cells and skeletal muscle from ESCs. More generally, the ability to produce and manipulate NMPs in vitro has the potential to increase the efficiency with which cell types derived from posterior neural and paraxial mesodermal tissue can be generated and analysed.

## Materials and Methods

### Animal and Human ES Cell Experiments

Animal experiments were performed under the UK Home Office project licenses PPL80/2528 and PPL60/4435, approved by the Animal Welfare and Ethical Review Panel of the MRC-National Institute for Medical Research and MRC Centre for Regenerative Medicine and within the conditions of the Animals (Scientific Procedures) Act 1986. Human Embryonic Stem Cell UK Steering Committee approval has been obtained (ref. SCSC14-09).

### Cell Culture and Differentiation

The mouse ES cell lines, HM1 [67] and BTBR10 [68] were maintained in ES cell medium [69] with 1000 U/ml LIF (Chemicon) on mitotically inactive primary mouse embryo fibroblasts. To initiate differentiation, ES cells were removed from feeders by dissociation using 0.05% trypsin and then plated onto tissue culture plates for two short successive periods (20–30 mins) to remove feeder layers. To induce differentiation, the cells were plated on CellBINDSurface dishes (Corning) precoated with 0.1% gelatin (Sigma) at a density of 5×10^3^ cells cm^−2^ in ‘N2B27’ medium. This medium comprised Advanced Dulbecco's Modified Eagle Medium F12 (Gibco) and Neurobasal medium (Gibco) (1∶1), supplemented with 1×N2 (Gibco), 1×B27 (Gibco), 2 mM L-glutamine (Gibco), 40 µg/ml BSA (Sigma), 0.1 mM 2-mercaptoethanol. Cells were grown in N2B27 supplemented with 10 ng/ml bFgf (R&D) for 3 days (D1–D3) and then were transferred into serum free media without bFgf (D3–D5). To induce ventral hindbrain identity NPCs (N_H_) 100 nM RA (Sigma) and 500 nM SAG (Calbiochem) was added from D3–D5. Spinal cord identity (N_P_) was induced by the addition of 5 µM CHIR99021 (Axon) or 100 ng/ml Wnt3a (R&D) from D2 to D3 followed by 100 nM RA, 500 nM SAG from D3–D5. To induce mesodermal differentiation the cells were treated with CHIR99021 from D2–D5. To induce terminal differentiation, cells were trypsinised and plated as single-cell suspension on plates coated with Matrigel (BD Biosciences) at a density of 1×10^5^ cells cm^−2^ in N2B27 medium supplemented with bFgf (10 ng/ml). The next day bFgf was removed and cells were left to differentiate for an additional 3 days.

The mouse EpiSC line R04-GFP [55] was routinely maintained in N2B27 supplemented with Activin A (20 ng/ml; R&D Systems) and bFgf (10 ng/ml; Peprotech) as previously described [70]. For differentiation of EpiSCs into NM progenitors approximately 1500–2000 cells/cm^2^ were plated on fibronectin (Sigma)-coated wells in N2B27 medium supplemented with CHIR99021 (3 µM; Signal Transduction Division, Dundee) and bFgf (20 ng/ml). For grafting experiments the initial plating density was 2500 cells/cm^2^ and cells were plated on either fibronectin or gelatin.

Human ESC lines MasterShef 5 and 7 (a gift of Prof. Harry Moore, University of Sheffield) and a Sox2GFP reporter line (a gift of Dr Andrew Smith, University of Edinburgh) were cultured in Essential 8™ medium on Geltrex™-coated plates. For hNMP differentiation cells were pre-treated for 1 h with ROCK inhibitor Y-27632 (10 µM; Calbiochem), dissociated with accutase and plated at approximately 10,000 cells/cm^2^ (Sox2-GFP hESCs) or 80,000 cells/cm^2^ (MasterShef5 and 7 hESC lines) on fibronectin-coated wells in N2B27 medium supplemented with 3 µM CHIR99021/20 ng/ml bFgf and Y-27632 (10 µM). The medium was replaced the following day with fresh N2B27 containing the same components minus the ROCK inhibitor. For directed differentiation of hESCs, cultures were differentiated in the presence of CHIR99021/bFgf for 72 h as described above. For neural/spinal cord differentiation 72 h CHIR99021/bFgf-differentiated cells were treated with Accutase (Sigma) and transferred onto Geltrex (Life Technologies)-coated plates either in N2B27 alone or N2B27 supplemented with RA (0.1 µM; Sigma), SAG (0.5 µM; Calbiochem) and purmorphamine (1 µM; Calbiochem) for 48 h. For mesodermal differentiation 72 h CHIR99021/bFgf differentiated cells were cultured in N2B27 supplemented with CHIR99021 (3 µM) for a further 48 h. For dual SMAD inhibition Sox2-GFP hES cells were plated at 10,000 cells/cm^2^ on Geltrex™-coated wells in N2B27 supplemented with LDN193189 (100 nM; Stemgent) and SB431542 (10 µM; Sigma). This was followed either by re-plating and culture in N2B27 or in N2B27/CHIR99021 (3 µM)/bFgf (20 ng/ml) for a further 48–72 h. All experiments involving hES cells have been approved by the UK Stem Cell Bank steering committee.

### Grafting of Mouse NMP Cells in Chick and Mouse Embryos

To graft NMP and N_A_ cells into the CLE of stage HH8–9 chick embryos, plates of appropriately prepared cells were labelled for 10 mins at 37°C with DiI and washed 3 times with PBS. N2B27 medium was added and cells were incubated for 30 mins. Small clumps of cells were mechanically detached from the plate and transplanted using a manually pulled glass needle. Groups of 100–200 cells were grafted into the caudal lateral epiblast and the eggs were incubated for a further 24 h. Embryos were then fixed in 4% paraformaldehyde (PFA) for 60 mins at 4°C. Fixed embryos were cryoprotected by equilibration in 15% sucrose and then cryosectioned (14 µm). Images were taken using an Apotome2 (Zeiss) and Leica confocal microscope TCS-SP5.

For mouse embryo grafting, r04-GFP EpiSC were flow sorted for GFP expression using a BD FACSAria II sorter and plated overnight in EpiSC conditions followed by Fgf/Wnt for 24 h or 48 h. Mouse embryo grafting (∼10 cells/embryo), culture and imaging were performed as described previously (Huang et al., 2012).

### Immunofluorescence

Cells were fixed for 10 minutes at 4°C in 4% paraformaldehyde in phosphate buffer saline (PBS), then washed in PBST (PBS with 0.1% Triton X-100). Blocking was for 1 h in PBST with 3% donkey serum at room temperature. Primary and secondary antibodies were diluted in PBST containing 1% donkey serum. Cells were incubated with primary antibodies overnight at 4°C, with secondary antibodies at room temperature for 2 h, mounted with DAPI containing Prolong Antifade (Molecular Probes), and fluorescent images were taken using an inverted Leica SP5 confocal microscope or an Apotome 2 microscope or an Olympus IX51 inverted microscope (Olympus). Embryo processing and immunohistochemistry on tissue sections was performed as described previously [55]. Whole embryos were imaged using a Nikon NZ100 dissecting microscope, and sections were imaged in an Olympus BX61 fluorescence compound microscope. Nuclear segmentation followed by single cell fluorescence quantification was performed as described previously [70]. The following primary antibodies were used: mouse anti-Hoxc6 (1∶10) (DSHB), mouse anti-Hoxc9 (1∶10) (gift of T. Jessell), mouse anti-Hoxc10 (1∶50) (DSHB), rat anti-Hoxb4 (1∶100) (gift of A. Gould), rabbit anti-Phox2b (1∶200), mouse anti-Tuj1 (1∶1000) (Covance), rabbit anti-Tuj1 (1∶500) (Covance), rabbit anti-Olig2 (1∶500) (Chemicon), mouse anti-Sox2 (1∶200) (ab92494, Abcam), rabbit anti-Sox2 (1∶200) (Millipore), goat anti-Sox2 (1∶100) (R&D), goat anti-Tbx6 (1∶200) (R&D) or rabbit anti-Tbx6 (0.6 µg/ml) (ab38883, Abcam), goat anti-Brachyury (1∶500) (R&D), rabbit anti-RALDH2 (1∶500) (Sigma), rabbit anti-Desmin (1∶500) (Abcam), mouse anti-MyoD1 (1∶200) (DAKO), mouse anti-Islet1 (1∶2000) (gift of T. Jessell), mouse anti-Lim3 (1∶10) (DSHB), mouse anti-HB9 (1∶100) (DSHB), anti-Foxa2 (1 mg/ml) (Santa Cruz; sc-6554), anti-GFP (10 µg/ml) (Abcam; ab13970), anti-Pax3 (1∶20) (DSHB);. anti-Nanog (2.5 µg/ml) (14-5761-80, eBioscience); anti-Oct4 (1 µg/ml) (N-19, Santa Cruz), HoxC8 (5 µg/ml) (Abcam). Secondary antibodies were anti-mouse, anti-rabbit, anti-goat and anti-rat, Alexa's (488, 568, 647) from Molecular Probes.

### Reverse Transcription–Quantitative PCR analysis

Total RNA was isolated from cells using the RNeasy kit (Qiagen) according to the manufacturer's instructions and digested with DNase I (Qiagen) to remove genomic DNA. First strand cDNA synthesis was performed with Superscript III system (Invitrogen) using random primers and amplified using Platinum SYBR-Green (Invitrogen). For QPCR the Applied Biosystems 7900HT Fast Real time PCR or the Light Cycler 480 SYBR Green I Master Mix (Roche) systems were used. PCR primers were designed using Primer3 software. All experiments were performed in biological duplicates or triplicates for each time point analysed. Expression values were normalized against the β-actin or the TATA-binding protein (TBP) and standard deviations were calculated and plotted using Prism 6 software (GraphPad). Primer sequences are available upon request.

### RNA-Sequencing and Data Analysis

Total RNA was processed according to the TruSeq protocol (Illumina). Three separate RNA libraries (biological replicates) were barcoded and prepared for each time point. Library size, purity and concentration were determined using Agilent Technologies 2100 Bioanalyzer with a DNA specific chip (Agilent DNA-1000). For sequencing, four samples were loaded per lane of an Illumina Genome Analyzer Hiseq2500. The sequence files generated each contained approximately 30million reads per sample. Reads were aligned to the Ensembl transcriptome mm10 using Bowtie2 and TopHat2 [71]. Per gene counts were collated using HTseq-count [72] and normalized using the DESeq R package [73]. Data analysis, PCA and Biplots were performed using custom scripts in R and MATLAB (MathWorks). RNA-seq data are available in the Array express database (http://www.ebi.ac.uk/arrayexpress) under accession number E-MTAB-2268.

## Supporting Information

Figure S1 **mESC derived neural progenitor cells respond to ventralising and posteriorising signals.** (A) Changes in the expression of the indicated ventral progenitors markers over time in N_A_, N_H_, and N_P_ conditions (RNA-seq data) exposed to the Shh agonist SAG. Nkx2-1 is induced only in N_A_ cells as expected, whereas Nkx2-2 is induced in all conditions. (B) Expression profile of Sox genes from D1 to D5 of differentiation in N_A_, N_H_, and N_P_ conditions. Sox1, specific for neural identity, is induced in all three conditions at D5 (RNA-seq data). (Note, log_2+_ scale). (C) Expression of posterior Hoxb and Hoxc gene clusters analysed by qRT-PCR in N_A_, N_H_ and N_P_ conditions at D5. (Note, log_2_ scale). This validates the mRNA-seq data shown in Figure 1B. (D) Otx2 is strongly expressed only in N_A_ cells as shown by immunostaining at D5 of differentiation. By contrast Hoxb4 is strongly expressed in N_H_ but in not N_A_ or N_P_ cells (representative images shown). All data used to generate the plots in Figure S1 can be found in Data S7.
(TIFF)

Figure S2 **Wnt controls the timing of Hox gene induction.** (A) Schematic illustrating the two differentiation conditions used in this experiment. In condition I, CHIR is added from D2 to D3, whereas in condition II CHIR is added from D3 to D4. (B) qRT-PCR shows the rapid induction of *Hoxb1* after CHIR addition. (C–D) qRT-PCR analysis shows that the timing of induction of *Hoxb* and *Hoxc* genes depends on the timing of Wnt treatment. (Note, log_2_ scale). (E) Cells exposed to a short pulse of FGF/CHIR, but not RA, express Hoxc10 at D8 of differentiation. (F) Immunostaining for Brachyury/Sox2 at day 3 of differentiation after a short pulse with Wnt3a/Fgf instead of CHIR/Fgf. Recombinant Wnt3a substituted for CHIR and NMP cells co-expressing Brachyury^+^ and Sox2^+^ were generated to a similar extent. All data used to generate the plots in Figure S2 can be found in Data S8.
(TIF)

Figure S3 **Identification of neural and mesodermal specific genes.** (A) Venn diagram indicating the number of genes that are specifically induced in each neural condition compared to mesodermal cells. (B) Venn diagram of genes induced specifically in mesodermal conditions compared to all neural conditions. The tables summarize the significantly differentially expressed genes identified using DESeq with FDR<0.1 and fold change >2. (C–D) PCA Biplots of the (C) first and second (PC1∼PC2) or (D) second and third (PC3∼PC2) principal components of a PCA performed with the 43 transcription factors that showed the highest variance across the data set. Samples are labelled in black and transcription factors labelled with red arrows; the arrow length is proportional to the variance of the transcription factor levels. Primary axes reflect the eigenvalues of the transcription factors, secondary axes reflect the eigenvector components of the samples. All sample triplicates are shown unless the labels of the same sample overlapped. Note the Biplot of the PC3∼PC2 indicates the separation of the R5 (N_H_) and W5 (N_P_) conditions along PC3.
(TIF)

Figure S4 **Optimising the induction of T^+^SOX2^+^ cells from mEpiSCs.** (A) The proportion of cells expressing Brachyury and/or Sox2 after 72 h of culture in different concentrations of CHIR99021 (CHIR) and bFgf followed by immunostaining and image analysis. Error bars = s.d. (n = 2). At least eight different fields/experiment were scored for each condition. (B) Time-course scoring of Brachyury (T) and Sox2 (S) expression in mEpiSC and hES cells cultured in the presence of FGF/CHIR for the indicated amounts of time. (C) Immunocytochemistry for Brachyury, Sox2 and Nanog expression in EpiSC cultures treated with FGF/CHIR for 48 h. (D) Immunocytochemistry SOX2 and OCT4 expression in hES cells treated with FGF/CHIR for 72 h. (E) Immunocytochemistry showing coexpression of Brachyury and Sox2 in transverse sections of E9.5 mouse embryos. All data used to generate the plots in Figure S4 can be found in Data S9.
(TIF)

Figure S5 **Differentiation potential of EpiSC- and hES-derived NMPs.** (A) qPCR analysis for indicated differentiation markers in EpiSCs cultured in the presence of FGF/CHIR for the indicated time periods. Error bars = s.d. (n = 2). (B) TBX6/SOX2 immunocytochemistry in EpiSC (top) and hES cells (bottom) differentiated for 96 h and 120 h respectively in FGF/CHIR. (C) qPCR analysis for indicated differentiation markers in hES cells cultured in the presence of FGF/CHIR. Error bars = s.d. (n = 2). (D) Top: Scheme describing the culture conditions employed for differentiation of FGF/CHIR-induced NM progenitors. Bottom: qPCR analysis for indicated differentiation markers in hES cells treated for 72 h with FGF/CHIR and then cultured in either RA/SAG/purmorphamine (green bars) or CHIR (brown bars). Error bars = s.d. (n = 2). (E) Representative images of HOXC8/SOX2 immunocytochemistry in hES cells differentiated for 72 h using dual SMAD inhibition followed by 48 h with FGF/CHIR (top) or hES cells differentiated for 120 h in FGF/CHIR (bottom). In all cases qPCR results are represented as log_10_ ratio of expression versus untreated EpiSCs (mouse) or hES cells (human). Anterior, anterior neural plate; PXM/SOM, paraxial/somitic mesoderm; LPM, lateral plate mesoderm; END, endoderm; PNP, posterior neural plate; SP, spinal cord; RA, retinoic acid; Pur, purmorphamine n/d, not determined. All data used to generate the plots in Figure S5 can be found in Data S10.
(TIFF)

Table S1 **List of genes induced only in NMP cells.** NMP specific genes were identified by collating genes significantly upregulated in NMP cells compared to D1 ES cells and D3 N_A_ cells that were not upregulated in D5 neural or mesoderm cells. Genes are shown with their Ensembl gene id number, short gene name and in each of the comparisons the fold change and p adjusted value is calculated using DESeq.
(DOCX)

Table S2 **List of the neural specific genes.** Pair wised comparisons identifies the genes which are induced in all neural conditions compared with mesodermal conditions at day 5. Genes are shown with their Ensembl gene_id number, short gene name and in each of the comparisons the fold change and p adjusted value is calculated using DESeq.
(DOCX)

Table S3 **List of the mesodermal specific genes.** Pair wised comparisons identifies the genes upregulated in mesodermal conditions compared with all neuronal conditions at day 5. Genes are shown with their Ensembl gene_id number, short gene name and in each of the comparisons the fold change and p adjusted value is calculated using DESeq.
(DOC)

Data S1 **Data used to generate plots in** **Figure 1** **.**
(XLSX)

Data S2 **Data used to generate plots in** **Figure 2** **.**
(XLSX)

Data S3 **Data used to generate heatmap in** **Figure 3** **.**
(XLSX)

Data S4 **Data used to generate plot in** **Figure 4** **.**
(XLSX)

Data S5 **Data used to generate plots in** **Figure 5** **.**
(XLSX)

Data S6 **Data used to generate plots in** **Figure 6** **.**
(XLSX)

Data S7 **Data used to generate plots in Figure S1.**
(XLSX)

Data S8 **Data used to generate plots in Figure S2.**
(XLSX)

Data S9 **Data used to generate plots in Figure S4.**
(XLS)

Data S10 **Data used to generate plots in Figure S5.**
(XLSX)

## Abbreviations

CLE: caudal lateral epiblast
EpiSC: epiblast stem cell
ESC: embryonic stem cell
NMP: neuromesodermal progenitor
NPC: neural progenitor cell
NSB: node-streak border
RA: retinoic acid
