# Supplementary figures and images for: In Vitro Generation of Neuromesodermal Progenitors Reveals Distinct Roles for Wnt Signalling in the Specification of Spinal Cord and Paraxial Mesoderm Identity
Source: PLoS Biol. 2014 Aug 26;12(8):e1001937. doi: 10.1371/journal.pbio.1001937 (PMC4144800; doi:10.1371/journal.pbio.1001937)

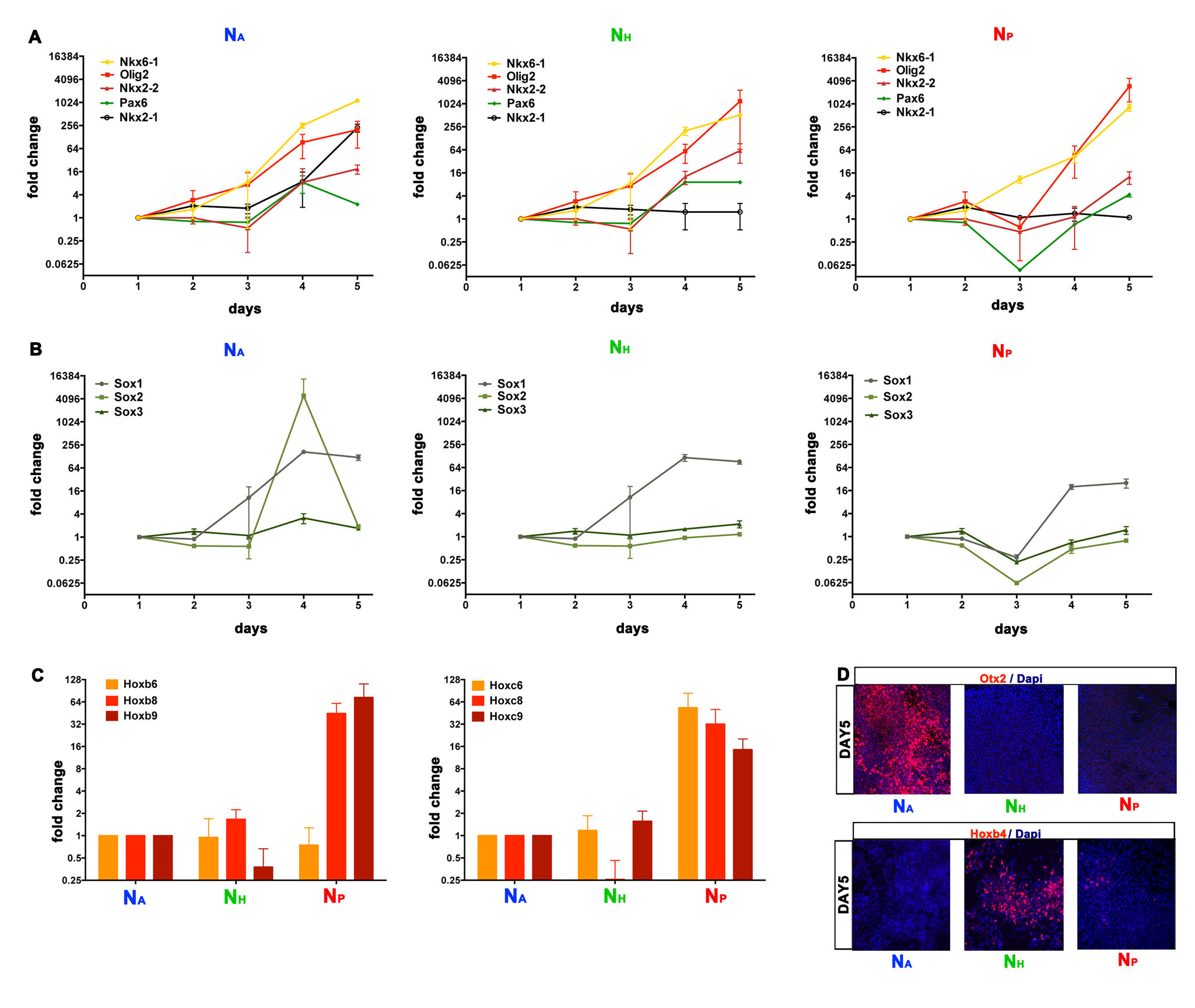

Supplement: Figure S1 — mESC derived neural progenitor cells respond to ventralising and posteriorising signals. (A) Changes in the expression of the indicated ventral progenitors markers over time in NA, NH, and NP conditions (RNA-seq data) exposed to the Shh agonist SAG. Nkx2-1 is induced only in NA cells as expected, whereas Nkx2-2 is induced in all conditions. (B) Expression profile of Sox genes from D1 to D5 of differentiation in NA, NH, and NP conditions. Sox1, specific for neural identity, is induced in all three conditions at D5 (RNA-seq data). (Note, log2+ scale). (C) Expression of posterior Hoxb and Hoxc gene clusters analysed by qRT-PCR in NA, NH and NP conditions at D5. (Note, log2 scale). This validates the mRNA-seq data shown in Figure 1B. (D) Otx2 is strongly expressed only in NA cells as shown by immunostaining at D5 of differentiation. By contrast Hoxb4 is strongly expressed in NH but in not NA or NP cells (representative images shown). All data used to generate the plots in Figure S1 can be found in Data S7. (TIFF) [file pbio.1001937.s001.tiff]

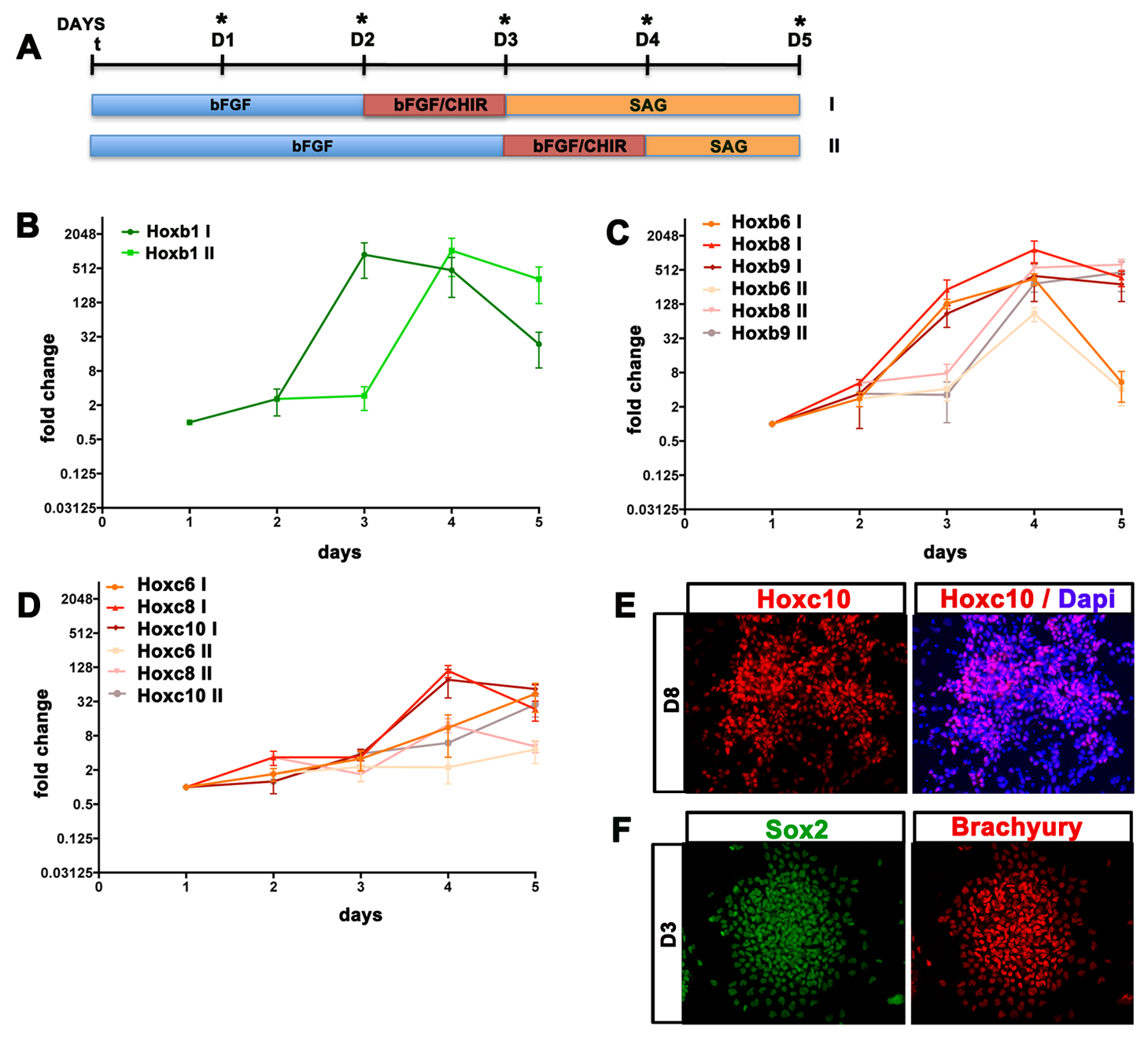

Supplement: Figure S2 — Wnt controls the timing of Hox gene induction. (A) Schematic illustrating the two differentiation conditions used in this experiment. In condition I, CHIR is added from D2 to D3, whereas in condition II CHIR is added from D3 to D4. (B) qRT-PCR shows the rapid induction of Hoxb1 after CHIR addition. (C–D) qRT-PCR analysis shows that the timing of induction of Hoxb and Hoxc genes depends on the timing of Wnt treatment. (Note, log2 scale). (E) Cells exposed to a short pulse of FGF/CHIR, but not RA, express Hoxc10 at D8 of differentiation. (F) Immunostaining for Brachyury/Sox2 at day 3 of differentiation after a short pulse with Wnt3a/Fgf instead of CHIR/Fgf. Recombinant Wnt3a substituted for CHIR and NMP cells co-expressing Brachyury+ and Sox2+ were generated to a similar extent. All data used to generate the plots in Figure S2 can be found in Data S8. (TIF) [file pbio.1001937.s002.tif]

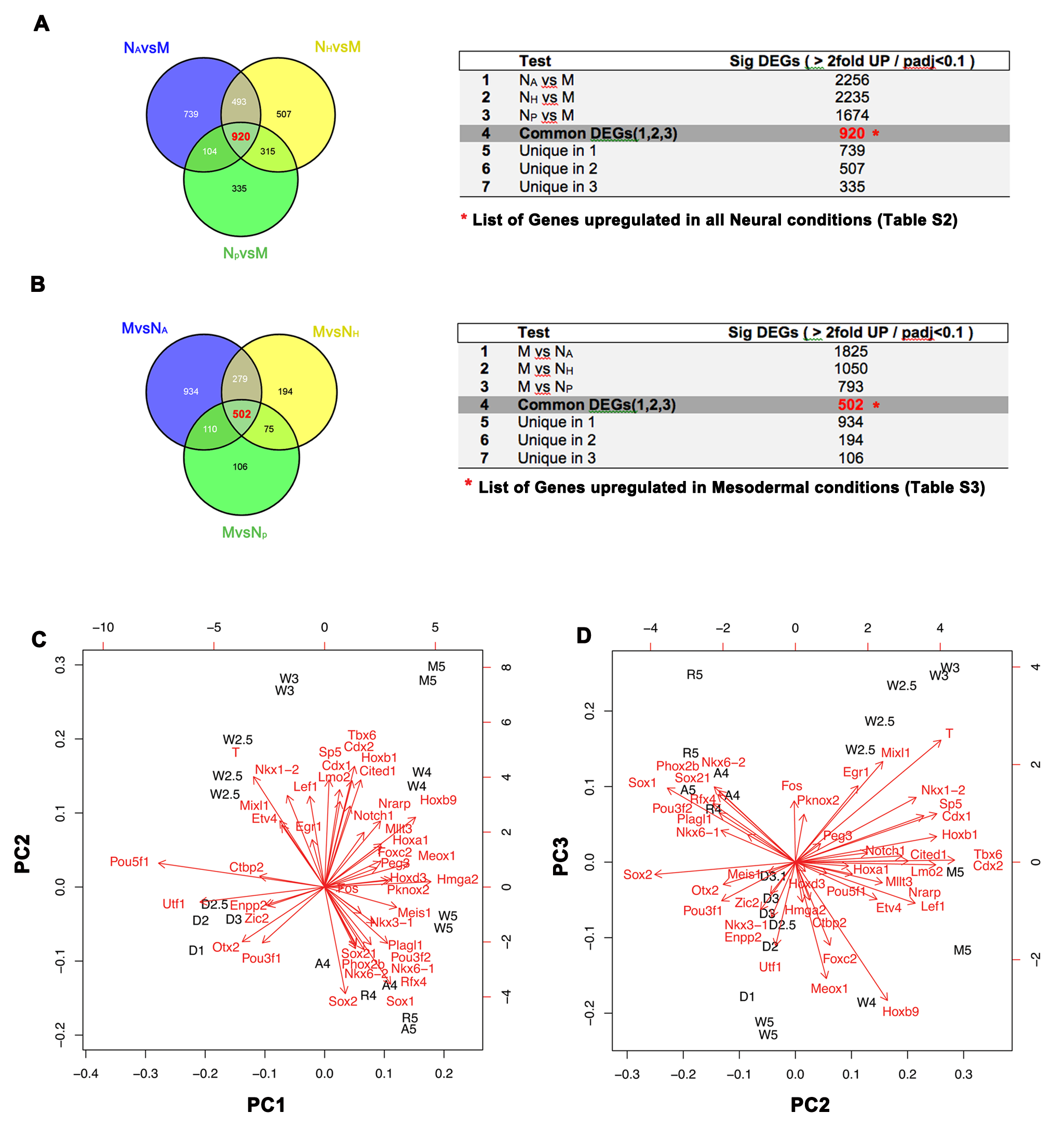

Supplement: Figure S3 — Identification of neural and mesodermal specific genes. (A) Venn diagram indicating the number of genes that are specifically induced in each neural condition compared to mesodermal cells. (B) Venn diagram of genes induced specifically in mesodermal conditions compared to all neural conditions. The tables summarize the significantly differentially expressed genes identified using DESeq with FDR<0.1 and fold change >2. (C–D) PCA Biplots of the (C) first and second (PC1∼PC2) or (D) second and third (PC3∼PC2) principal components of a PCA performed with the 43 transcription factors that showed the highest variance across the data set. Samples are labelled in black and transcription factors labelled with red arrows; the arrow length is proportional to the variance of the transcription factor levels. Primary axes reflect the eigenvalues of the transcription factors, secondary axes reflect the eigenvector components of the samples. All sample triplicates are shown unless the labels of the same sample overlapped. Note the Biplot of the PC3∼PC2 indicates the separation of the R5 (NH) and W5 (NP) conditions along PC3. (TIF) [file pbio.1001937.s003.tif]

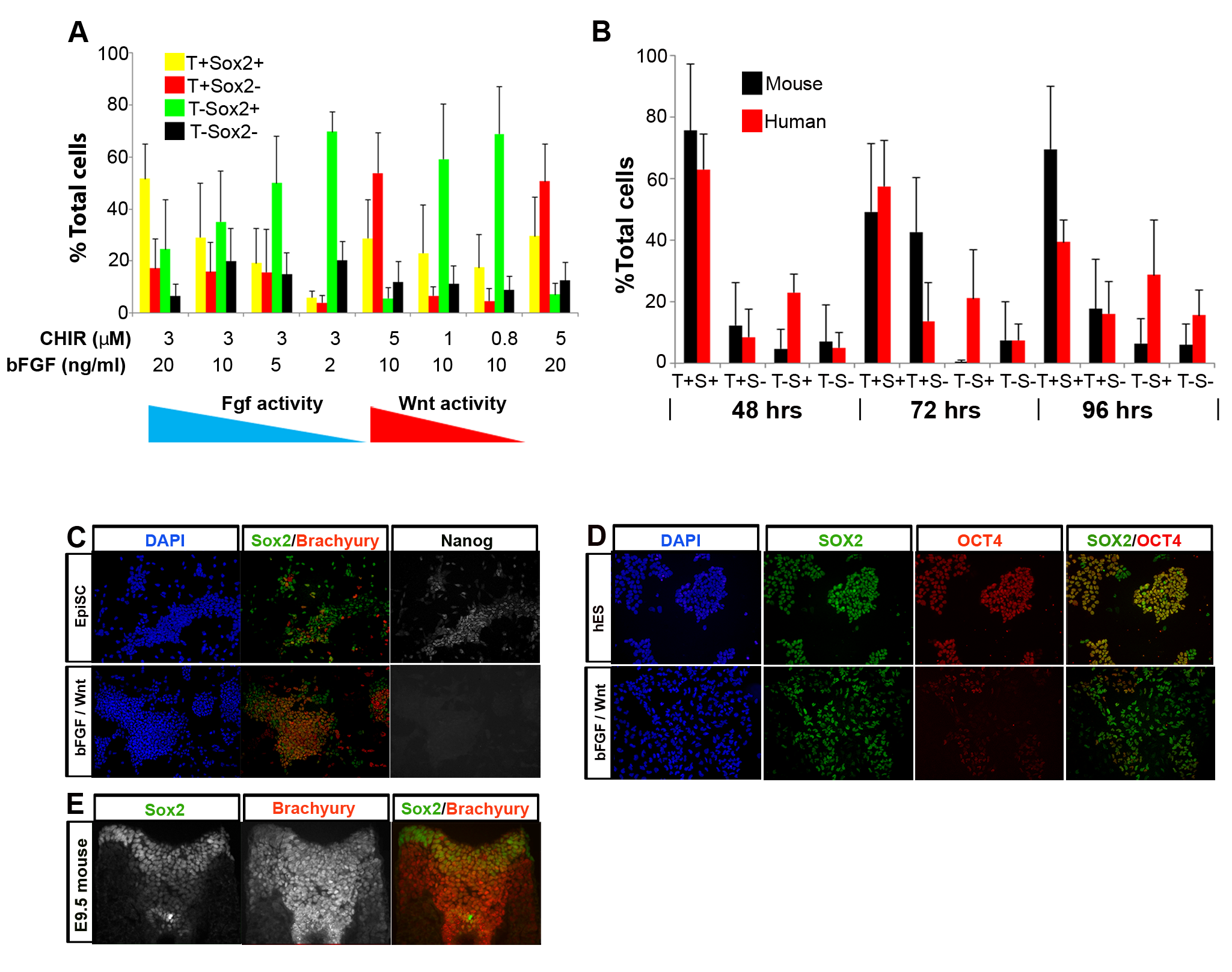

Supplement: Figure S4 — Optimising the induction of T+SOX2+ cells from mEpiSCs. (A) The proportion of cells expressing Brachyury and/or Sox2 after 72 h of culture in different concentrations of CHIR99021 (CHIR) and bFgf followed by immunostaining and image analysis. Error bars = s.d. (n = 2). At least eight different fields/experiment were scored for each condition. (B) Time-course scoring of Brachyury (T) and Sox2 (S) expression in mEpiSC and hES cells cultured in the presence of FGF/CHIR for the indicated amounts of time. (C) Immunocytochemistry for Brachyury, Sox2 and Nanog expression in EpiSC cultures treated with FGF/CHIR for 48 h. (D) Immunocytochemistry SOX2 and OCT4 expression in hES cells treated with FGF/CHIR for 72 h. (E) Immunocytochemistry showing coexpression of Brachyury and Sox2 in transverse sections of E9.5 mouse embryos. All data used to generate the plots in Figure S4 can be found in Data S9. (TIF) [file pbio.1001937.s004.tif]

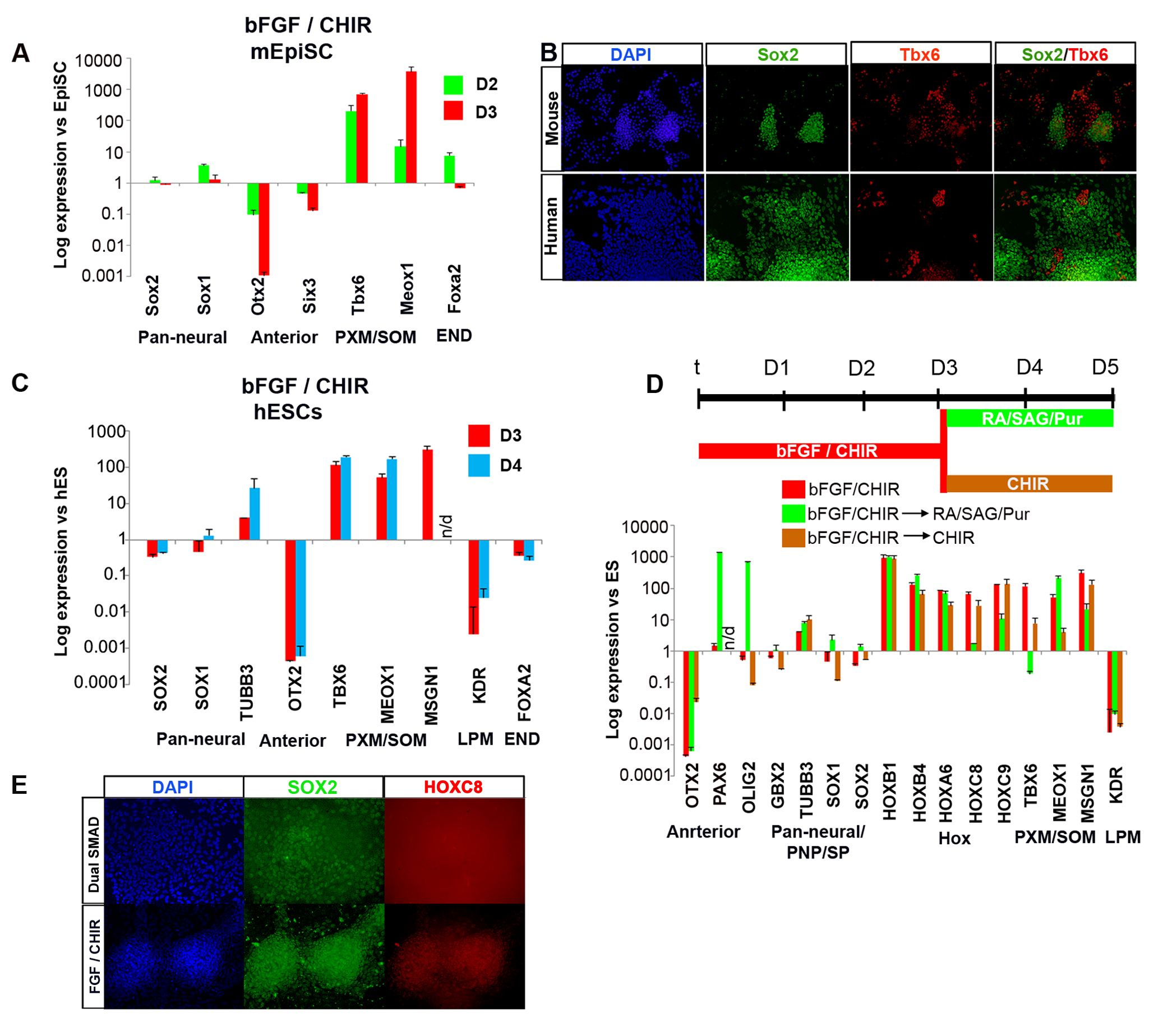

Supplement: Figure S5 — Differentiation potential of EpiSC- and hES-derived NMPs. (A) qPCR analysis for indicated differentiation markers in EpiSCs cultured in the presence of FGF/CHIR for the indicated time periods. Error bars = s.d. (n = 2). (B) TBX6/SOX2 immunocytochemistry in EpiSC (top) and hES cells (bottom) differentiated for 96 h and 120 h respectively in FGF/CHIR. (C) qPCR analysis for indicated differentiation markers in hES cells cultured in the presence of FGF/CHIR. Error bars = s.d. (n = 2). (D) Top: Scheme describing the culture conditions employed for differentiation of FGF/CHIR-induced NM progenitors. Bottom: qPCR analysis for indicated differentiation markers in hES cells treated for 72 h with FGF/CHIR and then cultured in either RA/SAG/purmorphamine (green bars) or CHIR (brown bars). Error bars = s.d. (n = 2). (E) Representative images of HOXC8/SOX2 immunocytochemistry in hES cells differentiated for 72 h using dual SMAD inhibition followed by 48 h with FGF/CHIR (top) or hES cells differentiated for 120 h in FGF/CHIR (bottom). In all cases qPCR results are represented as log10 ratio of expression versus untreated EpiSCs (mouse) or hES cells (human). Anterior, anterior neural plate; PXM/SOM, paraxial/somitic mesoderm; LPM, lateral plate mesoderm; END, endoderm; PNP, posterior neural plate; SP, spinal cord; RA, retinoic acid; Pur, purmorphamine n/d, not determined. All data used to generate the plots in Figure S5 can be found in Data S10. (TIFF) [file pbio.1001937.s005.tiff]
