# Supplementary material for: In Vitro Generation of Neuromesodermal Progenitors Reveals Distinct Roles for Wnt Signalling in the Specification of Spinal Cord and Paraxial Mesoderm Identity
Source: PLoS Biol. 2014 Aug 26;12(8):e1001937. doi: 10.1371/journal.pbio.1001937 (PMC4144800; doi:10.1371/journal.pbio.1001937)
Supplement: Table S1 — List of genes induced only in NMP cells. NMP specific genes were identified by collating genes significantly upregulated in NMP cells compared to D1 ES cells and D3 NA cells that were not upregulated in D5 neural or mesoderm cells. Genes are shown with their Ensembl gene id number, short gene name and in each of the comparisons the fold change and p adjusted value is calculated using DESeq. (DOCX) [file pbio.1001937.s006.docx]

| **ENSEMBL_Gene_ID** | **Gene_Name** | **Fold change**  **D5vsNMP** | **padj**  **D5vsNMP** | **Fold change**  **NMPvs D1** | **padj**  **NMPvsD1** | **Fold change.**  **NMPvsN_A_** | **padj**  **NMPvsN_A_** |
| --- | --- | --- | --- | --- | --- | --- | --- |
| ENSMUSG00000044062 | 0610009B14Rik | 0.35 | 0.005383234 | 4.01 | 6.08E-05 | 153.73 | 1.13E-16 |
| ENSMUSG00000045414 | 1190002N15Rik | 0.19 | 8.60E-20 | 4.76 | 1.58E-17 | 5.90 | 2.71E-21 |
| ENSMUSG00000058252 | 1700008I05Rik | 0.42 | 6.38E-05 | 4.62 | 5.63E-15 | 2.15 | 0.0002131 |
| ENSMUSG00000085549 | 1700047F07Rik | 0.03 | 3.30E-05 | 18.34 | 9.77E-05 | 13.50 | 0.000620639 |
| ENSMUSG00000087259 | 2610035D17Rik | 0.44 | 0.005713892 | 3.73 | 5.39E-07 | 2.01 | 0.012118739 |
| ENSMUSG00000085184 | 4933439K11Rik | 0.19 | 9.07E-06 | 9.69 | 9.50E-10 | 2.13 | 0.042330509 |
| ENSMUSG00000097844 | AC114995.1 | 0.29 | 0.005436391 | 14.61 | 5.82E-09 | Inf | 4.65E-14 |
| ENSMUSG00000097651 | AC139320.1 | 0.16 | 1.03E-16 | 2.19 | 0.000214384 | 23.71 | 1.73E-36 |
| ENSMUSG00000097503 | AC139673.1 | 0.45 | 0.038334296 | 2.55 | 0.00403844 | 3.56 | 0.000259143 |
| ENSMUSG00000097134 | AC153382.1 | 0.13 | 3.32E-11 | 11.99 | 6.66E-16 | 10.43 | 3.62E-14 |
| ENSMUSG00000096971 | AC158921.1 | 0.41 | 4.14E-05 | 3.36 | 3.66E-10 | 1.95 | 0.000984817 |
| ENSMUSG00000097040 | AC158956.1 | 0.20 | 1.54E-05 | 36.60 | 5.85E-15 | 5.19 | 9.01E-06 |
| ENSMUSG00000070985 | Acnat1 | 0.10 | 0.000729203 | 14.72 | 8.92E-06 | 21.12 | 1.77E-06 |
| ENSMUSG00000027605 | Acss2 | 0.36 | 7.69E-11 | 3.25 | 2.13E-15 | 2.20 | 2.81E-07 |
| ENSMUSG00000022220 | Adcy4 | 0.46 | 0.034240533 | 6.26 | 1.25E-09 | 2.40 | 0.003698876 |
| ENSMUSG00000058620 | Adra2b | 0.30 | 2.24E-08 | 14.10 | 1.98E-31 | 29.35 | 5.09E-41 |
| ENSMUSG00000011148 | Adssl1 | 0.31 | 3.39E-08 | 26.21 | 7.24E-47 | 3.11 | 5.79E-08 |
| ENSMUSG00000023353 | Agap3 | 0.46 | 5.02E-07 | 3.11 | 2.40E-15 | 1.60 | 0.00263511 |
| ENSMUSG00000030088 | Aldh1l1 | 0.50 | 0.002204174 | 5.57 | 8.23E-19 | 5.14 | 3.39E-17 |
| ENSMUSG00000018907 | Alox12e | 0.44 | 0.032056912 | 6.67 | 1.34E-08 | 65.11 | 2.99E-19 |
| ENSMUSG00000018924 | Alox15 | 0.39 | 0.0166361 | 3.66 | 5.53E-05 | 34.31 | 5.75E-23 |
| ENSMUSG00000014603 | Alx3 | 0.16 | 3.18E-20 | 43.64 | 9.10E-56 | 1.95 | 0.001005957 |
| ENSMUSG00000021314 | Amph | 0.44 | 6.83E-07 | 3.43 | 1.13E-16 | 2.27 | 8.66E-08 |
| ENSMUSG00000033420 | Antxr1 | 0.31 | 2.11E-14 | 4.76 | 5.12E-26 | 1.87 | 4.79E-05 |
| ENSMUSG00000062444 | Ap3b2 | 0.41 | 3.30E-05 | 4.51 | 1.34E-13 | 22.06 | 7.00E-42 |
| ENSMUSG00000037010 | Apln | 0.43 | 0.001720991 | 2.01 | 0.004096217 | 12.37 | 1.05E-23 |
| ENSMUSG00000044338 | Aplnr | 0.29 | 1.08E-05 | 17.05 | 1.38E-26 | 4.98 | 3.53E-10 |
| ENSMUSG00000020884 | Asgr1 | 0.22 | 7.37E-06 | 13.74 | 3.73E-14 | 3.53 | 7.29E-05 |
| ENSMUSG00000007097 | Atp1a2 | 0.04 | 8.02E-31 | 420.78 | 5.63E-73 | 2.86 | 6.44E-05 |
| ENSMUSG00000030730 | Atp2a1 | 0.37 | 0.00181253 | 7.83 | 2.33E-12 | 3.49 | 1.25E-05 |
| ENSMUSG00000028457 | Atp8b5 | 0.18 | 1.88E-07 | 4.98 | 1.60E-07 | 3.60 | 3.01E-05 |
| ENSMUSG00000026432 | Avpr1b | 0.10 | 5.44E-13 | 76.73 | 3.38E-32 | 9.13 | 1.04E-12 |
| ENSMUSG00000017929 | B4galt5 | 0.18 | 1.76E-31 | 5.15 | 2.31E-29 | 4.18 | 3.37E-22 |
| ENSMUSG00000032066 | Bco2 | 0.44 | 0.000539915 | 8.65 | 1.03E-19 | 3.35 | 4.96E-08 |
| ENSMUSG00000014329 | Bicc1 | 0.44 | 0.000749246 | 5.00 | 2.07E-14 | 2.20 | 0.000368227 |
| ENSMUSG00000008999 | Bmp7 | 0.33 | 6.36E-14 | 16.00 | 2.62E-67 | 2.89 | 1.53E-12 |
| ENSMUSG00000000706 | Btn1a1 | 0.05 | 2.19E-40 | 175.67 | 4.13E-72 | 12.73 | 2.39E-32 |
| ENSMUSG00000053216 | Btn2a2 | 0.16 | 2.18E-17 | 11.53 | 7.50E-27 | 7.59 | 1.10E-19 |
| ENSMUSG00000029544 | Cabp1 | 0.22 | 2.75E-19 | 5.44 | 2.28E-26 | 2.39 | 5.73E-08 |
| ENSMUSG00000040373 | Cacng5 | 0.36 | 0.006962116 | 26.23 | 8.89E-13 | 11.98 | 2.06E-09 |
| ENSMUSG00000054793 | Cadm4 | 0.36 | 3.78E-05 | 5.69 | 1.35E-13 | 1.83 | 0.023004689 |
| ENSMUSG00000038304 | Cd160 | 0.11 | 0.00466913 | 4.51 | 0.030980152 | 10.46 | 0.002292183 |
| ENSMUSG00000050840 | Cdh20 | 0.17 | 0.007068702 | 19.31 | 8.05E-07 | 49.47 | 2.72E-09 |
| ENSMUSG00000023067 | Cdkn1a | 0.32 | 1.61E-15 | 6.24 | 1.02E-36 | 2.27 | 2.20E-08 |
| ENSMUSG00000024268 | Celf4 | 0.27 | 1.09E-14 | 2.32 | 1.14E-07 | 2.59 | 1.03E-08 |
| ENSMUSG00000026450 | Chit1 | 0.08 | 1.32E-06 | 212.57 | 7.61E-13 | 13.90 | 6.07E-07 |
| ENSMUSG00000026251 | Chrnd | 0.27 | 0.002232756 | 3.78 | 0.000522344 | 2.72 | 0.014805439 |
| ENSMUSG00000037347 | Chst7 | 0.19 | 1.06E-17 | 9.65 | 1.26E-32 | 2.00 | 0.000453954 |
| ENSMUSG00000022715 | Cldn26 | 0.02 | 1.27E-13 | 41.40 | 1.12E-13 | 44.30 | 1.45E-13 |
| ENSMUSG00000039419 | Cntnap2 | 0.39 | 0.020286424 | 3.72 | 0.000122298 | 13.21 | 9.18E-13 |
| ENSMUSG00000030862 | Cpxm2 | 0.09 | 1.20E-16 | 35.03 | 2.40E-33 | 25.51 | 2.97E-28 |
| ENSMUSG00000042401 | Crtac1 | 0.23 | 2.32E-11 | 23.65 | 3.44E-36 | 2.35 | 0.0001624 |
| ENSMUSG00000032359 | Ctsh | 0.39 | 9.06E-06 | 2.69 | 3.36E-07 | 3.73 | 3.76E-11 |
| ENSMUSG00000067924 | Cxx1b | 0.32 | 5.30E-09 | 7.03 | 3.50E-23 | 2.50 | 5.05E-06 |
| ENSMUSG00000051851 | Cxx1c | 0.12 | 2.57E-23 | 21.46 | 2.05E-45 | 2.90 | 2.81E-07 |
| ENSMUSG00000090891 | D6Ertd527e | 0.30 | 0.023732931 | 8.39 | 6.37E-06 | 6.24 | 0.000106508 |
| ENSMUSG00000020108 | Ddit4 | 0.27 | 2.49E-09 | 12.68 | 9.73E-31 | 5.80 | 1.25E-16 |
| ENSMUSG00000036661 | Dennd3 | 0.30 | 1.39E-12 | 2.87 | 3.22E-11 | 8.86 | 2.18E-37 |
| ENSMUSG00000022861 | Dgkg | 0.27 | 1.24E-05 | 5.57 | 4.68E-10 | 2.03 | 0.014269527 |
| ENSMUSG00000031535 | Dkk4 | 0.00 | 1.52E-15 | 826.01 | 1.73E-14 | 11.70 | 0.000587613 |
| ENSMUSG00000043753 | Dmrta1 | 0.22 | 0.001210679 | 13.75 | 5.14E-09 | 3.22 | 0.012060786 |
| ENSMUSG00000055809 | Dnaaf3 | 0.47 | 2.10E-05 | 3.34 | 1.84E-14 | 4.55 | 5.06E-21 |
| ENSMUSG00000018581 | Dnahc11 | 0.14 | 0.001665013 | 5.46 | 0.001774807 | 4.44 | 0.008584069 |
| ENSMUSG00000036766 | Dner | 0.15 | 1.59E-09 | 20.03 | 2.26E-20 | 21.99 | 2.42E-20 |
| ENSMUSG00000031093 | Dock11 | 0.37 | 2.31E-11 | 5.84 | 2.80E-34 | 3.82 | 1.69E-20 |
| ENSMUSG00000044393 | Dsg2 | 0.37 | 1.27E-10 | 6.03 | 2.47E-34 | 7.19 | 7.13E-39 |
| ENSMUSG00000021791 | Dydc2 | 0.29 | 0.000964927 | 19.14 | 8.52E-13 | 12.34 | 4.89E-10 |
| ENSMUSG00000031168 | Ebp | 0.36 | 3.12E-11 | 2.88 | 6.14E-13 | 2.16 | 4.81E-07 |
| ENSMUSG00000027524 | Edn3 | 0.19 | 2.67E-10 | 22.00 | 3.20E-26 | 2.39 | 0.001140419 |
| ENSMUSG00000035105 | Egln3 | 0.26 | 8.05E-06 | 6.12 | 9.14E-11 | 7.99 | 4.61E-13 |
| ENSMUSG00000043460 | Elfn2 | 0.23 | 1.42E-06 | 5.92 | 2.83E-09 | 65.72 | 1.40E-21 |
| ENSMUSG00000044072 | Eml6 | 0.49 | 0.007457209 | 2.59 | 1.39E-05 | 3.87 | 4.69E-09 |
| ENSMUSG00000015085 | Entpd2 | 0.39 | 0.026064207 | 8.78 | 1.73E-09 | 7.40 | 5.59E-08 |
| ENSMUSG00000006311 | Etv2 | 0.35 | 0.000698812 | 2.50 | 0.00105118 | 6.53 | 8.79E-11 |
| ENSMUSG00000030630 | Fah | 0.41 | 1.56E-08 | 4.77 | 5.48E-27 | 2.82 | 9.80E-13 |
| ENSMUSG00000030956 | Fam53b | 0.44 | 2.26E-07 | 2.89 | 3.64E-13 | 1.96 | 9.62E-06 |
| ENSMUSG00000042444 | Fam63b | 0.41 | 3.72E-08 | 6.47 | 1.65E-34 | 2.42 | 9.60E-09 |
| ENSMUSG00000030019 | Fbxl14 | 0.35 | 4.11E-13 | 2.75 | 1.40E-12 | 1.81 | 0.000113187 |
| ENSMUSG00000055197 | Fev | 0.13 | 0.001520235 | 17.02 | 1.14E-05 | 11.07 | 0.000177473 |
| ENSMUSG00000031603 | Fgf20 | 0.03 | 2.92E-07 | 11.99 | 1.36E-05 | 8.55 | 0.000267226 |
| ENSMUSG00000045326 | Fndc7 | 0.03 | 5.35E-05 | 6.57 | 0.004930726 | 65.17 | 1.68E-05 |
| ENSMUSG00000019779 | Frk | 0.42 | 0.002293315 | 2.15 | 0.001996003 | 2.71 | 0.000145844 |
| ENSMUSG00000021765 | Fst | 0.07 | 1.67E-17 | 18.60 | 1.78E-21 | 33.86 | 3.26E-28 |
| ENSMUSG00000036264 | Fstl4 | 0.16 | 1.39E-14 | 102.10 | 9.67E-52 | 11.32 | 7.72E-24 |
| ENSMUSG00000046152 | Fut10 | 0.44 | 7.95E-07 | 3.70 | 5.24E-18 | 1.59 | 0.004718921 |
| ENSMUSG00000071335 | G630090E17Rik | 0.30 | 0.017624233 | 5.49 | 6.50E-05 | 13.18 | 2.35E-07 |
| ENSMUSG00000039809 | Gabbr2 | 0.32 | 4.93E-06 | 33.67 | 8.60E-33 | 78.80 | 6.06E-37 |
| ENSMUSG00000031343 | Gabra3 | 0.43 | 5.13E-07 | 2.56 | 1.40E-09 | 6.91 | 3.70E-33 |
| ENSMUSG00000029211 | Gabra4 | 0.27 | 2.89E-09 | 2.12 | 0.000531165 | 10.81 | 3.59E-23 |
| ENSMUSG00000031344 | Gabrq | 0.22 | 0.000780993 | 13.55 | 9.77E-09 | 58.98 | 8.54E-12 |
| ENSMUSG00000070880 | Gad1 | 0.08 | 3.07E-25 | 54.31 | 1.38E-48 | 1.97 | 0.005612904 |
| ENSMUSG00000091387 | Gcnt4 | 0.37 | 0.039752503 | 4.05 | 0.00040511 | 19.75 | 8.31E-12 |
| ENSMUSG00000022103 | Gfra2 | 0.24 | 5.90E-16 | 12.19 | 2.03E-42 | 9.79 | 1.55E-35 |
| ENSMUSG00000046352 | Gjb2 | 0.39 | 0.015879212 | 9.99 | 1.85E-12 | 2.84 | 0.002178468 |
| ENSMUSG00000085586 | Gm11613 | 0.34 | 1.63E-05 | 3.23 | 2.09E-07 | 1.70 | 0.044272943 |
| ENSMUSG00000087200 | Gm12908 | 0.33 | 0.000236889 | 6.43 | 3.23E-10 | 3.74 | 3.36E-06 |
| ENSMUSG00000086326 | Gm13200 | 0.00 | 0.00678992 | Inf | 0.002575045 | Inf | 0.005597503 |
| ENSMUSG00000086905 | Gm13716 | 0.19 | 0.002541313 | 7.13 | 0.000144272 | 4.48 | 0.005267622 |
| ENSMUSG00000087292 | Gm13832 | 0.12 | 0.008861279 | 6.05 | 0.007766555 | 5.83 | 0.019049458 |
| ENSMUSG00000086166 | Gm14342 | 0.26 | 4.10E-06 | 34.12 | 1.44E-18 | 4.70 | 2.10E-07 |
| ENSMUSG00000074444 | Gm15284 | 0.02 | 5.32E-11 | 72.75 | 2.93E-13 | 7.55 | 0.000164649 |
| ENSMUSG00000078117 | Gm16485 | 0.35 | 0.041301733 | 8.14 | 1.34E-06 | 4.68 | 0.000417088 |
| ENSMUSG00000053367 | Gm6792 | 0.13 | 2.83E-14 | 2.32 | 0.001003015 | 32.21 | 2.74E-31 |
| ENSMUSG00000024697 | Gna14 | 0.04 | 3.83E-16 | 13.55 | 2.70E-12 | 14.53 | 3.29E-12 |
| ENSMUSG00000021340 | Gpld1 | 0.35 | 1.23E-09 | 4.33 | 5.64E-20 | 2.14 | 4.30E-06 |
| ENSMUSG00000031210 | Gpr165 | 0.20 | 0.001564253 | 9.45 | 1.25E-05 | 4.40 | 0.003640151 |
| ENSMUSG00000045281 | Gpr20 | 0.40 | 0.018952308 | 7.96 | 6.01E-08 | 5.56 | 3.99E-06 |
| ENSMUSG00000040125 | Gpr26 | 0.20 | 5.86E-07 | 7.82 | 1.53E-11 | 218.10 | 1.59E-25 |
| ENSMUSG00000049649 | Gpr3 | 0.35 | 0.003629795 | 4.41 | 4.72E-06 | 2.06 | 0.035327455 |
| ENSMUSG00000056380 | Gpr50 | 0.27 | 0.014907322 | 5.72 | 0.000583342 | 11.44 | 1.20E-05 |
| ENSMUSG00000020734 | Grin2c | 0.49 | 0.03688655 | 13.20 | 7.06E-19 | 50.86 | 8.19E-30 |
| ENSMUSG00000044221 | Grsf1 | 0.09 | 3.46E-32 | 11.03 | 1.23E-33 | 3.81 | 9.21E-12 |
| ENSMUSG00000030606 | Hapln3 | 0.43 | 0.000287343 | 6.32 | 5.94E-16 | 3.70 | 7.13E-09 |
| ENSMUSG00000067438 | Hmx1 | 0.21 | 0.000276622 | 3.07 | 0.004462644 | 3.92 | 0.001406862 |
| ENSMUSG00000026322 | Htr4 | 0.27 | 0.029757225 | 4.66 | 0.002970276 | 26.81 | 9.66E-07 |
| ENSMUSG00000050534 | Htr5b | 0.28 | 0.007832024 | 2.35 | 0.049958103 | 7.16 | 6.84E-05 |
| ENSMUSG00000024798 | Htr7 | 0.37 | 0.000817563 | 4.41 | 1.67E-07 | 5.57 | 9.66E-09 |
| ENSMUSG00000031549 | Ido2 | 0.33 | 8.18E-05 | 6.46 | 7.05E-12 | 3.59 | 1.92E-06 |
| ENSMUSG00000076617 | Ighm | 0.35 | 0.009898163 | 4.14 | 0.000170638 | 22.04 | 2.04E-11 |
| ENSMUSG00000027895 | Kcnc4 | 0.46 | 0.031755405 | 7.29 | 8.22E-11 | 2.28 | 0.014321496 |
| ENSMUSG00000022342 | Kcnv1 | 0.14 | 0.000106189 | 15.04 | 1.04E-07 | 7.99 | 2.19E-05 |
| ENSMUSG00000028758 | Kif17 | 0.41 | 0.008458344 | 3.44 | 1.51E-05 | 2.79 | 0.000710128 |
| ENSMUSG00000040387 | Klhl32 | 0.11 | 2.02E-26 | 24.41 | 1.32E-48 | 4.13 | 3.69E-13 |
| ENSMUSG00000035606 | Ky | 0.41 | 0.001230459 | 5.52 | 5.69E-11 | 2.57 | 0.000323936 |
| ENSMUSG00000042363 | Lgalsl | 0.29 | 1.37E-16 | 4.57 | 4.89E-26 | 2.38 | 4.75E-09 |
| ENSMUSG00000042793 | Lgr6 | 0.39 | 0.013049742 | 6.99 | 5.62E-09 | 3.51 | 0.000275215 |
| ENSMUSG00000030946 | Lhpp | 0.28 | 3.21E-18 | 2.37 | 2.30E-09 | 3.54 | 2.19E-18 |
| ENSMUSG00000053846 | Lipg | 0.48 | 7.48E-06 | 3.76 | 9.11E-19 | 2.62 | 1.66E-10 |
| ENSMUSG00000025185 | Loxl4 | 0.38 | 5.70E-05 | 8.65 | 4.07E-21 | 24.66 | 1.23E-36 |
| ENSMUSG00000028613 | Lrp8 | 0.24 | 1.30E-13 | 6.26 | 5.85E-23 | 2.86 | 2.47E-08 |
| ENSMUSG00000075224 | Lrrc55 | 0.30 | 0.000105875 | 9.00 | 4.78E-12 | 11.01 | 1.77E-12 |
| ENSMUSG00000022375 | Lrrc6 | 0.33 | 0.009261768 | 31.07 | 4.24E-15 | 7.00 | 5.37E-07 |
| ENSMUSG00000067206 | Lrrc66 | 0.00 | 0.000341139 | 11.38 | 0.008121738 | 7.12 | 0.045692101 |
| ENSMUSG00000036306 | Lzts1 | 0.18 | 1.77E-22 | 12.10 | 4.98E-42 | 72.61 | 4.23E-77 |
| ENSMUSG00000030041 | M1ap | 0.16 | 8.85E-30 | 6.73 | 6.03E-33 | 10.45 | 3.33E-45 |
| ENSMUSG00000037306 | Man1c1 | 0.30 | 1.63E-14 | 6.98 | 1.13E-36 | 2.32 | 4.27E-08 |
| ENSMUSG00000024085 | Man2a1 | 0.34 | 4.01E-13 | 3.50 | 1.45E-18 | 2.46 | 7.96E-10 |
| ENSMUSG00000024235 | Map3k8 | 0.45 | 0.000730815 | 4.19 | 5.75E-12 | 3.41 | 7.07E-09 |
| ENSMUSG00000071856 | Mcc | 0.11 | 2.02E-17 | 8.10 | 1.78E-17 | 3.21 | 2.77E-06 |
| ENSMUSG00000026497 | Mixl1 | 0.03 | 0.000676744 | 35.37 | 9.20E-05 | 45.80 | 6.92E-05 |
| ENSMUSG00000061013 | Mkx | 0.45 | 5.46E-06 | 2.93 | 5.14E-11 | 5.46 | 7.85E-23 |
| ENSMUSG00000064036 | Mro | 0.25 | 0.002738377 | 3.82 | 0.001007657 | 3.98 | 0.001294088 |
| ENSMUSG00000072720 | Myo18b | 0.48 | 0.005947022 | 5.86 | 1.82E-12 | 8.97 | 2.33E-16 |
| ENSMUSG00000034057 | Myrfl | 0.04 | 9.31E-22 | 22.17 | 4.35E-21 | 2.75 | 0.000857866 |
| ENSMUSG00000071203 | Naip5 | 0.31 | 0.000189517 | 8.13 | 9.57E-13 | 26.28 | 5.60E-22 |
| ENSMUSG00000078942 | Naip6 | 0.22 | 3.85E-06 | 10.51 | 1.53E-14 | 29.08 | 1.49E-23 |
| ENSMUSG00000020181 | Nav3 | 0.13 | 2.92E-07 | 17.11 | 3.74E-13 | 8.94 | 9.02E-09 |
| ENSMUSG00000054850 | Ncrna00086 | 0.28 | 7.04E-17 | 3.80 | 3.65E-19 | 6.86 | 1.28E-35 |
| ENSMUSG00000029832 | Nfe2l3 | 0.26 | 6.66E-12 | 7.56 | 2.00E-24 | 6.35 | 7.40E-20 |
| ENSMUSG00000048528 | Nkx1-2 | 0.07 | 5.28E-07 | 16.00 | 8.68E-08 | 23.26 | 4.04E-09 |
| ENSMUSG00000035984 | Nme5 | 0.41 | 3.36E-05 | 8.47 | 4.83E-24 | 1.72 | 0.013397305 |
| ENSMUSG00000032456 | Nmnat3 | 0.35 | 0.00920363 | 3.29 | 0.000530198 | 4.66 | 2.25E-05 |
| ENSMUSG00000055994 | Nod2 | 0.21 | 2.21E-16 | 17.57 | 4.50E-43 | 3.22 | 1.70E-10 |
| ENSMUSG00000035528 | Npffr2 | 0.14 | 0.000260045 | 24.30 | 4.76E-08 | 57.83 | 4.57E-09 |
| ENSMUSG00000026602 | Nphs2 | 0.23 | 1.32E-06 | 21.19 | 7.33E-19 | 26.46 | 1.65E-19 |
| ENSMUSG00000042684 | Npl | 0.11 | 6.04E-43 | 7.94 | 5.64E-39 | 1.69 | 0.001596474 |
| ENSMUSG00000020598 | Nrcam | 0.45 | 0.005110082 | 11.09 | 1.05E-21 | 22.24 | 1.22E-31 |
| ENSMUSG00000024109 | Nrxn1 | 0.39 | 7.16E-05 | 4.44 | 3.27E-12 | 26.09 | 2.64E-36 |
| ENSMUSG00000032420 | Nt5e | 0.21 | 5.69E-09 | 22.88 | 5.13E-30 | 24.22 | 6.29E-30 |
| ENSMUSG00000040258 | Nxph4 | 0.49 | 0.023394944 | 2.84 | 0.000241082 | 8.95 | 1.38E-11 |
| ENSMUSG00000034755 | Pcdh11x | 0.14 | 1.85E-11 | 30.70 | 1.30E-27 | 3.96 | 5.78E-07 |
| ENSMUSG00000031595 | Pdgfrl | 0.21 | 1.51E-22 | 11.40 | 3.84E-50 | 8.39 | 1.95E-39 |
| ENSMUSG00000067825 | Pex26 | 0.49 | 2.44E-05 | 2.15 | 4.68E-07 | 2.03 | 8.04E-06 |
| ENSMUSG00000030413 | Pglyrp1 | 0.08 | 3.59E-05 | 6.27 | 0.000791486 | 26.74 | 1.20E-07 |
| ENSMUSG00000031870 | Pgr | 0.37 | 9.05E-09 | 8.01 | 1.64E-34 | 6.14 | 1.88E-26 |
| ENSMUSG00000045658 | Pid1 | 0.45 | 1.70E-07 | 4.15 | 3.17E-22 | 10.08 | 2.76E-49 |
| ENSMUSG00000038167 | Plekhg6 | 0.22 | 1.24E-06 | 11.79 | 4.52E-16 | 4.28 | 1.25E-06 |
| ENSMUSG00000041653 | Pnpla3 | 0.26 | 6.19E-18 | 5.86 | 5.02E-30 | 11.33 | 1.17E-49 |
| ENSMUSG00000070366 | Ppapdc1a | 0.09 | 3.90E-09 | 8.06 | 4.12E-08 | 6.78 | 1.04E-06 |
| ENSMUSG00000050558 | Prokr2 | 0.34 | 3.30E-05 | 5.07 | 1.85E-10 | 5.94 | 1.26E-11 |
| ENSMUSG00000048752 | Prss50 | 0.44 | 0.000141786 | 2.11 | 0.000206485 | 3.12 | 5.13E-08 |
| ENSMUSG00000015090 | Ptgds | 0.11 | 2.95E-06 | 20.46 | 7.22E-11 | 3.13 | 0.015779272 |
| ENSMUSG00000047250 | Ptgs1 | 0.10 | 1.84E-24 | 6.79 | 2.27E-19 | 34.39 | 6.32E-48 |
| ENSMUSG00000025314 | Ptprj | 0.50 | 2.24E-05 | 2.62 | 8.59E-11 | 1.90 | 5.73E-05 |
| ENSMUSG00000055069 | Rab39 | 0.48 | 0.010720789 | 3.05 | 2.83E-06 | 12.12 | 2.22E-19 |
| ENSMUSG00000034472 | Rasd2 | 0.46 | 0.003989769 | 2.42 | 0.00023393 | 6.37 | 5.09E-14 |
| ENSMUSG00000041696 | Rasl12 | 0.33 | 0.000126444 | 2.09 | 0.005459558 | 2.24 | 0.004079653 |
| ENSMUSG00000022236 | Ropn1l | 0.48 | 0.000763628 | 8.28 | 7.28E-21 | 2.35 | 2.55E-05 |
| ENSMUSG00000070691 | Runx3 | 0.21 | 5.16E-13 | 2.61 | 4.49E-06 | 3.53 | 2.72E-09 |
| ENSMUSG00000034463 | Scara3 | 0.32 | 3.24E-05 | 7.35 | 1.88E-15 | 2.22 | 0.002440032 |
| ENSMUSG00000000037 | Scml2 | 0.22 | 3.12E-10 | 6.69 | 3.94E-17 | 2.06 | 0.0025964 |
| ENSMUSG00000070304 | Scn2b | 0.06 | 1.54E-32 | 19.12 | 9.25E-39 | 20.34 | 2.84E-39 |
| ENSMUSG00000046480 | Scn4b | 0.29 | 4.77E-07 | 10.65 | 2.08E-18 | 1.76 | 0.032071653 |
| ENSMUSG00000030340 | Scnn1a | 0.41 | 4.14E-05 | 12.50 | 7.41E-29 | 1.58 | 0.040133935 |
| ENSMUSG00000000627 | Sema4f | 0.18 | 1.84E-24 | 8.95 | 5.04E-39 | 8.76 | 6.70E-37 |
| ENSMUSG00000030638 | Sh3gl3 | 0.49 | 6.11E-06 | 3.78 | 2.30E-19 | 2.84 | 4.95E-12 |
| ENSMUSG00000018387 | Shroom1 | 0.44 | 0.007304329 | 3.21 | 5.70E-06 | 2.44 | 0.000956628 |
| ENSMUSG00000022696 | Sidt1 | 0.15 | 0.035988525 | 17.70 | 0.000248103 | 13.26 | 0.001266306 |
| ENSMUSG00000057058 | Skap1 | 0.04 | 2.55E-52 | 86.49 | 1.37E-72 | 7.94 | 5.99E-26 |
| ENSMUSG00000020805 | Slc13a5 | 0.37 | 0.003873148 | 11.04 | 3.02E-13 | 4.25 | 2.43E-06 |
| ENSMUSG00000037762 | Slc16a9 | 0.44 | 0.002988975 | 2.73 | 2.32E-05 | 3.01 | 1.70E-05 |
| ENSMUSG00000038496 | Slc19a3 | 0.42 | 6.84E-05 | 5.37 | 6.09E-17 | 3.59 | 3.07E-10 |
| ENSMUSG00000031170 | Slc38a5 | 0.32 | 4.51E-07 | 12.50 | 1.30E-25 | 14.44 | 1.25E-26 |
| ENSMUSG00000034224 | Slc38a8 | 0.03 | 2.91E-07 | 13.64 | 8.43E-06 | 9.33 | 0.00014984 |
| ENSMUSG00000027075 | Slc43a1 | 0.45 | 6.93E-07 | 2.78 | 2.58E-12 | 1.66 | 0.001205709 |
| ENSMUSG00000021733 | Slc4a7 | 0.42 | 1.13E-08 | 3.87 | 1.73E-21 | 1.52 | 0.011731403 |
| ENSMUSG00000052026 | Slc6a7 | 0.06 | 1.52E-09 | 2.41 | 0.027229941 | 2.66 | 0.02651762 |
| ENSMUSG00000031129 | Slc9a9 | 0.05 | 4.35E-46 | 26.65 | 6.43E-51 | 14.25 | 2.67E-37 |
| ENSMUSG00000025938 | Slco5a1 | 0.38 | 0.023286007 | 7.41 | 1.05E-08 | 4.54 | 2.22E-05 |
| ENSMUSG00000036790 | Slitrk2 | 0.47 | 0.019057912 | 3.32 | 3.40E-05 | 3.76 | 1.48E-05 |
| ENSMUSG00000036169 | Sostdc1 | 0.22 | 9.87E-05 | 8.14 | 2.06E-08 | 2.30 | 0.04165641 |
| ENSMUSG00000005883 | Spo11 | 0.21 | 0.011668311 | 16.43 | 5.38E-07 | 18.22 | 6.18E-07 |
| ENSMUSG00000074445 | Sprr2a3 | 0.05 | 4.15E-07 | 36.43 | 4.65E-10 | 387.15 | 4.50E-18 |
| ENSMUSG00000031216 | Stard8 | 0.43 | 4.42E-06 | 5.60 | 2.39E-23 | 1.75 | 0.002701313 |
| ENSMUSG00000004043 | Stat5a | 0.37 | 4.67E-09 | 9.82 | 3.46E-42 | 2.46 | 3.02E-08 |
| ENSMUSG00000039215 | Svs1 | 0.03 | 0.048419115 | Inf | 0.006738506 | 25.87 | 0.03913927 |
| ENSMUSG00000048277 | Syngr2 | 0.28 | 1.51E-17 | 4.33 | 3.88E-24 | 2.57 | 1.96E-10 |
| ENSMUSG00000031255 | Sytl4 | 0.37 | 3.43E-05 | 5.86 | 4.40E-14 | 3.06 | 1.11E-06 |
| ENSMUSG00000062327 | T | 0.15 | 1.84E-08 | 16.06 | 9.50E-18 | 9.50 | 2.24E-12 |
| ENSMUSG00000061762 | Tac1 | 0.15 | 0.0006272 | 7.14 | 0.000100521 | 31.86 | 9.55E-08 |
| ENSMUSG00000060985 | Tdrd5 | 0.47 | 9.20E-06 | 2.67 | 6.62E-11 | 31.31 | 2.08E-82 |
| ENSMUSG00000054003 | Tdrd9 | 0.24 | 6.51E-05 | 7.26 | 2.25E-08 | 3.73 | 0.000107717 |
| ENSMUSG00000016150 | Tenm1 | 0.11 | 1.14E-16 | 11.96 | 1.44E-20 | 35.72 | 2.16E-33 |
| ENSMUSG00000009628 | Tex15 | 0.40 | 0.001933691 | 4.28 | 3.80E-08 | 2.53 | 0.000923447 |
| ENSMUSG00000053469 | Tg | 0.04 | 0.00107256 | 47.43 | 6.35E-05 | 7.38 | 0.024930724 |
| ENSMUSG00000025013 | Tll2 | 0.22 | 1.30E-05 | 14.06 | 1.63E-12 | 2.87 | 0.002387777 |
| ENSMUSG00000024799 | Tm7sf2 | 0.41 | 1.08E-08 | 2.06 | 9.22E-07 | 2.16 | 2.87E-07 |
| ENSMUSG00000050106 | Tmc8 | 0.42 | 0.011014347 | 2.73 | 0.000276553 | 5.43 | 2.56E-09 |
| ENSMUSG00000046593 | Tmem215 | 0.15 | 8.43E-08 | 14.95 | 5.33E-13 | 3.26 | 0.000248283 |
| ENSMUSG00000030341 | Tnfrsf1a | 0.34 | 7.16E-13 | 3.75 | 4.01E-19 | 1.71 | 0.000646659 |
| ENSMUSG00000035678 | Tnfsf9 | 0.35 | 1.25E-09 | 5.95 | 7.37E-25 | 3.27 | 4.84E-12 |
| ENSMUSG00000055322 | Tns1 | 0.20 | 1.18E-06 | 2.89 | 0.000729026 | 2.43 | 0.012343105 |
| ENSMUSG00000017607 | Tns4 | 0.17 | 3.77E-07 | 6.30 | 1.39E-08 | 2.08 | 0.04451453 |
| ENSMUSG00000027716 | Trpc3 | 0.34 | 0.005477607 | 5.34 | 6.88E-07 | 3.48 | 0.000356622 |
| ENSMUSG00000030523 | Trpm1 | 0.01 | 3.02E-32 | 42.33 | 1.70E-29 | 8.17 | 4.97E-14 |
| ENSMUSG00000030137 | Tuba8 | 0.37 | 0.000623189 | 11.73 | 1.27E-15 | 6.59 | 2.62E-10 |
| ENSMUSG00000057948 | Unc13d | 0.47 | 0.001022287 | 5.28 | 2.10E-16 | 2.65 | 2.38E-06 |
| ENSMUSG00000050830 | Vwc2 | 0.10 | 1.46E-07 | 8.78 | 1.30E-07 | 11.61 | 1.35E-08 |
| ENSMUSG00000026167 | Wnt10a | 0.03 | 2.32E-14 | 100.30 | 2.34E-20 | 19.99 | 1.83E-11 |
| ENSMUSG00000029671 | Wnt16 | 0.48 | 0.041469261 | 3.31 | 0.000163479 | 3.25 | 0.000413306 |
| ENSMUSG00000010797 | Wnt2 | 0.03 | 8.15E-07 | 20.19 | 2.11E-06 | 28.47 | 6.14E-07 |
| ENSMUSG00000027840 | Wnt2b | 0.26 | 7.46E-06 | 8.70 | 1.05E-12 | 11.48 | 1.81E-14 |
| ENSMUSG00000009900 | Wnt3a | 0.01 | 3.21E-18 | 113.92 | 1.25E-21 | 14.90 | 1.22E-09 |
| ENSMUSG00000030170 | Wnt5b | 0.06 | 4.43E-27 | 15.82 | 7.84E-29 | 1.93 | 0.012695625 |
| ENSMUSG00000033227 | Wnt6 | 0.02 | 6.51E-30 | 243.95 | 4.13E-45 | 3.87 | 3.17E-05 |
| ENSMUSG00000012282 | Wnt8a | 0.28 | 0.029803784 | 9.49 | 6.60E-06 | 18.59 | 2.82E-08 |
| ENSMUSG00000037005 | Xpnpep2 | 0.32 | 0.000447761 | 8.18 | 1.58E-11 | 4.97 | 1.77E-07 |
| ENSMUSG00000044646 | Zbtb7c | 0.49 | 0.00381417 | 5.00 | 3.64E-11 | 2.55 | 7.48E-05 |
| ENSMUSG00000043456 | Zfp536 | 0.30 | 0.015880593 | 3.15 | 0.006496575 | 3.05 | 0.013249733 |
| ENSMUSG00000062743 | Zfp677 | 0.40 | 2.02E-08 | 7.60 | 4.28E-37 | 4.30 | 1.83E-20 |
| ENSMUSG00000041961 | Znrf3 | 0.34 | 8.15E-13 | 2.62 | 1.18E-10 | 1.59 | 0.008102525 |
