# Supplementary material for: In Vitro Generation of Neuromesodermal Progenitors Reveals Distinct Roles for Wnt Signalling in the Specification of Spinal Cord and Paraxial Mesoderm Identity
Source: PLoS Biol. 2014 Aug 26;12(8):e1001937. doi: 10.1371/journal.pbio.1001937 (PMC4144800; doi:10.1371/journal.pbio.1001937)
Supplement: Table S2 — List of the neural specific genes. Pair wised comparisons identifies the genes which are induced in all neural conditions compared with mesodermal conditions at day 5. Genes are shown with their Ensembl gene_id number, short gene name and in each of the comparisons the fold change and p adjusted value is calculated using DESeq. (DOCX) [file pbio.1001937.s007.docx]

| **ENSEMBL GENE_ID** | **GENE_NAME** | **Fold change** | **padj** | **Fold change** | **padj** | **Fold change** | **padj** |
| --- | --- | --- | --- | --- | --- | --- | --- |
|  |  | **NAvsM** | **NAvsM** | **NHvsM** | **NHvsM** | **NPvsM** | **NPvsM** |
| ENSMUSG00000022997 | Wnt1 | 3601.64 | 1.95E-11 | 121.05 | 8.63E-05 | 365.09 | 1.06E-06 |
| ENSMUSG00000048015 | Neurod4 | 1648.07 | 0.000416735 | 940.72 | 0.001143062 | 1059.46 | 0.001245526 |
| ENSMUSG00000024076 | Vit | 1529.9 | 1.99E-13 | 3945.65 | 3.64E-16 | 131.77 | 2.53E-06 |
| ENSMUSG00000027434 | Nkx2-2 | 750.47 | 0.000165032 | 215.27 | 0.001895322 | 46.92 | 0.033806624 |
| ENSMUSG00000047935 | Gm5607 | 677.16 | 2.31E-20 | 1239.85 | 1.41E-23 | 247.92 | 7.08E-15 |
| ENSMUSG00000044361 | BC024139 | 396.6 | 3.40E-14 | 398.12 | 3.03E-14 | 30.92 | 0.000113442 |
| ENSMUSG00000039830 | Olig2 | 360.17 | 2.91E-09 | 2082.53 | 2.00E-13 | 5595.94 | 9.93E-16 |
| ENSMUSG00000096753 | Fam181a | 352.12 | 9.22E-07 | 978.21 | 2.25E-08 | 440.92 | 7.00E-07 |
| ENSMUSG00000058669 | Nkx2-9 | 343.05 | 1.94E-19 | 156.72 | 5.77E-16 | 41.34 | 4.38E-10 |
| ENSMUSG00000068154 | Insm1 | 341.57 | 1.92E-07 | 141.44 | 5.35E-06 | 26 | 0.003109941 |
| ENSMUSG00000041309 | Nkx6-2 | 330.08 | 2.36E-38 | 301.89 | 7.81E-38 | 20.82 | 6.37E-14 |
| ENSMUSG00000024227 | 2610034M16Rik | 318.45 | 3.62E-17 | 275.25 | 1.68E-16 | 25.95 | 0.001032632 |
| ENSMUSG00000022494 | Shisa9 | 265.84 | 1.68E-21 | 140.1 | 1.54E-15 | 12.6 | 0.039341778 |
| ENSMUSG00000061517 | Sox21 | 216.81 | 1.15E-15 | 84.82 | 3.47E-12 | 15.51 | 7.20E-06 |
| ENSMUSG00000097767 | Miat | 187.56 | 3.59E-17 | 203.23 | 1.09E-17 | 48.78 | 3.46E-11 |
| ENSMUSG00000022122 | Ednrb | 187.15 | 9.60E-24 | 415.88 | 6.01E-29 | 115.02 | 1.22E-20 |
| ENSMUSG00000000202 | Btbd17 | 181.04 | 1.45E-32 | 154.28 | 3.59E-31 | 217.57 | 2.35E-34 |
| ENSMUSG00000052848 | C130026L21Rik | 180.57 | 8.51E-11 | 174.74 | 1.38E-10 | 73.35 | 1.71E-07 |
| ENSMUSG00000026185 | Igfbp5 | 173.31 | 1.50E-15 | 164.81 | 1.75E-15 | 152.97 | 6.17E-15 |
| ENSMUSG00000037025 | Foxa2 | 159.98 | 1.11E-48 | 76.9 | 2.86E-39 | 29.08 | 3.94E-26 |
| ENSMUSG00000046160 | Olig1 | 135.71 | 2.27E-09 | 315.95 | 1.90E-13 | 606.17 | 2.18E-16 |
| ENSMUSG00000090828 | Sox2ot | 134.28 | 1.59E-05 | 122.41 | 2.20E-05 | 33.28 | 0.001745449 |
| ENSMUSG00000003410 | Elavl3 | 130.95 | 6.57E-25 | 111.17 | 7.39E-24 | 37.14 | 1.83E-15 |
| ENSMUSG00000020037 | Rfx4 | 123.57 | 1.87E-18 | 164.6 | 3.29E-20 | 24.56 | 4.88E-10 |
| ENSMUSG00000042269 | Fam92b | 120.23 | 5.69E-10 | 69.9 | 2.91E-07 | 42.49 | 3.25E-05 |
| ENSMUSG00000000632 | Sez6 | 118.6 | 6.66E-34 | 384.91 | 4.49E-52 | 14.64 | 8.28E-09 |
| ENSMUSG00000089760 | D030046N08Rik | 116.01 | 9.40E-34 | 103.2 | 2.57E-31 | 71.02 | 5.53E-25 |
| ENSMUSG00000090534 | Gm4675 | 114.58 | 1.87E-13 | 22.88 | 3.17E-06 | 14.36 | 0.000241082 |
| ENSMUSG00000030310 | Slc6a1 | 108.16 | 2.58E-10 | 23.74 | 9.44E-05 | 10.51 | 0.020330617 |
| ENSMUSG00000010021 | Kif19a | 104.98 | 5.61E-39 | 122.03 | 1.54E-41 | 66.56 | 3.76E-29 |
| ENSMUSG00000074607 | Tox2 | 103.62 | 1.21E-06 | 32.03 | 0.000142104 | 12.1 | 0.007391773 |
| ENSMUSG00000096014 | Sox1 | 98.52 | 2.53E-31 | 74.42 | 2.98E-29 | 20.26 | 1.03E-16 |
| ENSMUSG00000090125 | Pou3f1 | 92.95 | 5.60E-79 | 15.02 | 4.51E-33 | 4.24 | 4.06E-09 |
| ENSMUSG00000031965 | Tbx20 | 92.77 | 4.28E-09 | 29.05 | 6.39E-06 | 6.15 | 0.036790664 |
| ENSMUSG00000086141 | 9030622O22Rik | 90.05 | 5.90E-22 | 32.78 | 1.76E-14 | 9.56 | 2.02E-06 |
| ENSMUSG00000085338 | 2410004I01Rik | 89.69 | 8.36E-14 | 41.04 | 2.15E-07 | 44.83 | 1.66E-07 |
| ENSMUSG00000025876 | Unc5a | 84.64 | 4.19E-06 | 393.9 | 8.64E-09 | 55.27 | 3.61E-05 |
| ENSMUSG00000028946 | Hes3 | 84.44 | 1.38E-05 | 102.17 | 3.63E-06 | 110 | 3.69E-06 |
| ENSMUSG00000022577 | Ly6h | 80.11 | 7.79E-06 | 58.11 | 3.08E-05 | 19.87 | 0.002485457 |
| ENSMUSG00000062209 | Erbb4 | 78.99 | 7.06E-17 | 16.55 | 4.25E-08 | 6.01 | 0.001622489 |
| ENSMUSG00000027581 | Stmn3 | 78.96 | 1.04E-63 | 40.21 | 7.81E-48 | 19.86 | 9.78E-32 |
| ENSMUSG00000095139 | Pou3f2 | 72.09 | 8.52E-11 | 37.08 | 1.30E-08 | 17.23 | 5.68E-06 |
| ENSMUSG00000038156 | Spon1 | 70.18 | 7.22E-68 | 28 | 5.58E-47 | 14.77 | 5.93E-33 |
| ENSMUSG00000030621 | Me3 | 67.9 | 0.000169847 | 63.18 | 0.000252807 | 13.03 | 0.04055549 |
| ENSMUSG00000047904 | Sstr2 | 66.27 | 5.63E-11 | 123.74 | 4.59E-14 | 69.88 | 4.34E-11 |
| ENSMUSG00000017453 | Pipox | 63.75 | 2.43E-117 | 82.54 | 3.58E-130 | 11.55 | 7.95E-46 |
| ENSMUSG00000025469 | Msx3 | 62.49 | 8.37E-16 | 4.14 | 0.040773131 | 11.52 | 1.35E-05 |
| ENSMUSG00000028341 | Nr4a3 | 62.37 | 5.38E-23 | 13.45 | 4.64E-10 | 12.63 | 2.88E-09 |
| ENSMUSG00000079598 | Clec2l | 62.02 | 1.48E-14 | 80.25 | 8.01E-16 | 9.79 | 1.45E-05 |
| ENSMUSG00000051515 | Fam181b | 60.68 | 2.44E-14 | 8.96 | 1.71E-05 | 32.08 | 5.31E-11 |
| ENSMUSG00000051251 | Nhlh1 | 59.21 | 8.67E-05 | 72.38 | 5.20E-05 | 81.15 | 4.50E-05 |
| ENSMUSG00000075020 | E530001K10Rik | 57.59 | 1.62E-30 | 49.96 | 1.68E-28 | 22.67 | 2.33E-17 |
| ENSMUSG00000045871 | Slitrk6 | 56.96 | 4.20E-51 | 5.83 | 1.87E-08 | 3.07 | 0.005328638 |
| ENSMUSG00000087150 | BC064078 | 56.43 | 4.83E-11 | 28.38 | 1.84E-07 | 21.28 | 4.70E-06 |
| ENSMUSG00000072945 | Ripply1 | 52.66 | 2.98E-08 | 41.59 | 3.41E-07 | 131.78 | 4.23E-12 |
| ENSMUSG00000042607 | Asb4 | 49.83 | 1.24E-21 | 7.06 | 1.39E-06 | 15.67 | 3.27E-12 |
| ENSMUSG00000040181 | Fmo1 | 49.61 | 0.000190954 | 205.95 | 1.80E-08 | 22.95 | 0.008470508 |
| ENSMUSG00000052551 | Adarb2 | 49.48 | 6.65E-08 | 13.37 | 0.0019877 | 14.36 | 0.001883762 |
| ENSMUSG00000025576 | Rbfox3 | 49.15 | 4.40E-56 | 11.77 | 1.79E-24 | 9.16 | 1.86E-19 |
| ENSMUSG00000085323 | 9130410C08Rik | 48.18 | 2.58E-09 | 24.9 | 4.01E-05 | 563.72 | 3.55E-39 |
| ENSMUSG00000018822 | Sfrp5 | 47.82 | 9.77E-13 | 759.48 | 1.10E-36 | 248.41 | 2.86E-26 |
| ENSMUSG00000028785 | Hpca | 47.53 | 4.20E-26 | 198.6 | 4.90E-64 | 75.82 | 1.74E-36 |
| ENSMUSG00000087307 | Gm12925 | 47.46 | 4.32E-16 | 12.78 | 3.29E-06 | 5.18 | 0.04545437 |
| ENSMUSG00000027070 | Lrp2 | 47.28 | 4.31E-45 | 68.92 | 8.30E-53 | 8.59 | 2.73E-17 |
| ENSMUSG00000055254 | Ntrk2 | 45.88 | 1.31E-24 | 105.27 | 2.10E-36 | 27.14 | 9.70E-19 |
| ENSMUSG00000043531 | Sorcs1 | 45.85 | 4.12E-71 | 33.51 | 4.41E-61 | 6.48 | 2.14E-15 |
| ENSMUSG00000055210 | Foxd2 | 45.37 | 2.43E-22 | 20.22 | 2.29E-14 | 244.35 | 1.67E-42 |
| ENSMUSG00000022053 | Ebf2 | 44.81 | 2.85E-08 | 12.25 | 0.000126357 | 21.79 | 4.48E-06 |
| ENSMUSG00000049154 | Fam183b | 44.44 | 1.65E-10 | 93.77 | 7.34E-15 | 79.2 | 9.04E-14 |
| ENSMUSG00000069170 | Gpr98 | 44.06 | 4.24E-33 | 52.19 | 1.61E-35 | 8.39 | 6.12E-13 |
| ENSMUSG00000025551 | Fgf14 | 43.89 | 2.31E-22 | 13.78 | 1.77E-11 | 5.97 | 4.50E-05 |
| ENSMUSG00000027584 | Oprl1 | 43.6 | 1.79E-47 | 42.93 | 8.66E-49 | 10.46 | 4.01E-15 |
| ENSMUSG00000051435 | Fhad1 | 41.39 | 2.83E-25 | 34.02 | 5.85E-23 | 12.17 | 1.89E-10 |
| ENSMUSG00000032181 | Scg3 | 40.85 | 8.95E-06 | 33.39 | 2.19E-05 | 28.52 | 6.53E-05 |
| ENSMUSG00000033316 | Galnt9 | 40.83 | 2.94E-20 | 31.05 | 2.04E-16 | 19.68 | 1.44E-10 |
| ENSMUSG00000039639 | Kcne1 | 40.41 | 0.000469178 | 136.51 | 8.61E-07 | 20.63 | 0.01474314 |
| ENSMUSG00000051359 | Ncald | 40.27 | 2.40E-36 | 16.41 | 2.92E-23 | 8.05 | 2.12E-13 |
| ENSMUSG00000009075 | Cabp7 | 40.02 | 4.80E-20 | 38.85 | 5.97E-20 | 63.83 | 1.76E-24 |
| ENSMUSG00000031891 | Hsd11b2 | 39.5 | 1.53E-06 | 14.4 | 0.000370904 | 74.27 | 6.09E-08 |
| ENSMUSG00000059857 | Ntng1 | 39.25 | 8.39E-16 | 19.94 | 8.03E-11 | 24.28 | 5.47E-12 |
| ENSMUSG00000047992 | Fam69c | 38.44 | 4.38E-47 | 51.91 | 2.77E-55 | 39.15 | 9.44E-48 |
| ENSMUSG00000030411 | Nova2 | 37.21 | 3.73E-51 | 15.27 | 9.04E-31 | 4.2 | 3.69E-08 |
| ENSMUSG00000027419 | Pcsk2 | 37.01 | 6.15E-05 | 43.26 | 1.44E-05 | 17.35 | 0.011140462 |
| ENSMUSG00000047085 | Lrrc4b | 35.63 | 1.26E-54 | 19.45 | 3.04E-39 | 5.99 | 3.95E-14 |
| ENSMUSG00000034958 | Atcay | 35.15 | 1.13E-35 | 8.82 | 1.29E-10 | 4.72 | 0.0001936 |
| ENSMUSG00000063239 | Grm4 | 34.72 | 4.52E-54 | 31.31 | 2.00E-51 | 13.64 | 5.69E-29 |
| ENSMUSG00000055003 | Lrtm2 | 33.98 | 3.26E-05 | 17.97 | 0.003860595 | 19.07 | 0.002032553 |
| ENSMUSG00000074637 | Sox2 | 33.83 | 1.19E-82 | 20.67 | 2.09E-67 | 14 | 2.68E-53 |
| ENSMUSG00000029875 | AI836003 | 33.26 | 4.44E-14 | 14.4 | 1.25E-07 | 8.59 | 0.000307988 |
| ENSMUSG00000004366 | Sst | 33.24 | 0.002070858 | 62.34 | 0.000197647 | 34.36 | 0.002428574 |
| ENSMUSG00000039257 | Vstm2b | 33.17 | 1.36E-35 | 13.97 | 5.93E-23 | 3.75 | 1.00E-06 |
| ENSMUSG00000060257 | Scrt2 | 33.07 | 0.013724862 | 25.32 | 0.031552129 | 24.67 | 0.037375336 |
| ENSMUSG00000041633 | Kctd12b | 33.04 | 1.16E-45 | 5.3 | 2.32E-12 | 12.94 | 5.14E-27 |
| ENSMUSG00000059173 | Pde1a | 32.74 | 2.94E-06 | 34.37 | 4.29E-07 | 12.73 | 0.014680838 |
| ENSMUSG00000001260 | Gabrg1 | 32.66 | 2.57E-08 | 9.23 | 0.001043221 | 28.8 | 8.10E-08 |
| ENSMUSG00000057123 | Gja5 | 32.53 | 9.25E-07 | 16.06 | 0.002361304 | 17.06 | 0.002405864 |
| ENSMUSG00000037946 | Fgd3 | 31.84 | 3.53E-26 | 45.98 | 4.23E-31 | 7.48 | 5.67E-10 |
| ENSMUSG00000078640 | Gm11627 | 31.49 | 2.85E-45 | 7.57 | 7.80E-16 | 7.94 | 4.16E-16 |
| ENSMUSG00000053093 | Myh7 | 31.25 | 5.21E-10 | 25.17 | 8.74E-09 | 7.87 | 0.000454406 |
| ENSMUSG00000097621 | AC163355.1 | 31.1 | 8.93E-10 | 32.58 | 1.03E-10 | 33.05 | 2.06E-10 |
| ENSMUSG00000021700 | Rab3c | 30.77 | 0.001968646 | 14.7 | 0.024443253 | 19.52 | 0.012381413 |
| ENSMUSG00000022197 | Pdzd2 | 30.7 | 1.87E-89 | 49.2 | 1.32E-111 | 15.78 | 2.47E-62 |
| ENSMUSG00000085389 | 1700003M07Rik | 29.92 | 8.63E-28 | 8.75 | 7.60E-10 | 5.15 | 5.53E-05 |
| ENSMUSG00000056812 | St8sia3 | 29.86 | 1.01E-27 | 14.28 | 1.03E-18 | 7.41 | 1.87E-10 |
| ENSMUSG00000015222 | Map2 | 29.75 | 2.54E-86 | 8.89 | 7.99E-41 | 4.52 | 5.69E-20 |
| ENSMUSG00000037034 | Pax1 | 29.63 | 0.029108777 | 30.04 | 0.040407011 | 895.46 | 0.000154608 |
| ENSMUSG00000026686 | Lmx1a | 29.55 | 6.30E-18 | 7.7 | 2.27E-07 | 10.46 | 3.04E-09 |
| ENSMUSG00000097794 | AC154412.1 | 28.94 | 6.77E-05 | 18.67 | 0.000398946 | 9.96 | 0.018816677 |
| ENSMUSG00000066392 | Nrxn3 | 28.88 | 1.37E-09 | 35.92 | 1.23E-11 | 13.81 | 2.73E-05 |
| ENSMUSG00000035451 | Foxa1 | 28.86 | 6.04E-18 | 7.96 | 3.08E-08 | 4.63 | 6.89E-05 |
| ENSMUSG00000087620 | 5330434G04Rik | 28.54 | 3.93E-07 | 18.74 | 1.31E-05 | 8.5 | 0.005387034 |
| ENSMUSG00000035472 | Slc25a21 | 27.95 | 3.25E-08 | 42.43 | 4.48E-10 | 27.24 | 4.01E-08 |
| ENSMUSG00000035187 | Nkx6-1 | 27.81 | 5.59E-12 | 12.71 | 5.29E-08 | 20.21 | 3.77E-10 |
| ENSMUSG00000045174 | Amer3 | 27.39 | 7.82E-05 | 19.5 | 0.001469232 | 13.66 | 0.020862679 |
| ENSMUSG00000009772 | Nuak2 | 27.33 | 1.68E-87 | 18.83 | 4.48E-73 | 12.79 | 1.14E-56 |
| ENSMUSG00000001270 | Ckb | 26.89 | 2.65E-20 | 41.48 | 2.39E-24 | 8.12 | 7.75E-10 |
| ENSMUSG00000047773 | Ankfn1 | 26.71 | 5.27E-06 | 17.36 | 0.000288579 | 10.27 | 0.010179446 |
| ENSMUSG00000045954 | Sdpr | 26.15 | 3.27E-26 | 23.61 | 2.06E-25 | 13.38 | 7.08E-15 |
| ENSMUSG00000043110 | Lrrn4 | 25.98 | 1.68E-32 | 38.71 | 1.20E-40 | 11.88 | 1.32E-19 |
| ENSMUSG00000043168 | 4930426D05Rik | 25.81 | 8.62E-05 | 22.82 | 0.000130884 | 25.82 | 9.05E-05 |
| ENSMUSG00000022454 | Nell2 | 25.46 | 6.17E-17 | 17.75 | 3.60E-14 | 11.73 | 7.71E-11 |
| ENSMUSG00000039899 | Fgl2 | 24.95 | 2.10E-35 | 9.86 | 1.34E-17 | 2.39 | 0.031916856 |
| ENSMUSG00000053046 | Brsk2 | 24.76 | 2.19E-16 | 17.56 | 7.99E-14 | 9.43 | 4.12E-09 |
| ENSMUSG00000028137 | Celf3 | 24.55 | 3.34E-05 | 16.64 | 0.000276459 | 5.94 | 0.028870587 |
| ENSMUSG00000022269 | March1 | 24.53 | 4.13E-08 | 16.15 | 2.51E-06 | 9.51 | 0.002475983 |
| ENSMUSG00000050100 | Hmx2 | 23.83 | 0.005885715 | 20.78 | 0.015822979 | 30.14 | 0.003799127 |
| ENSMUSG00000068735 | Trp53i11 | 23.83 | 1.71E-65 | 17.47 | 6.09E-56 | 15.13 | 3.76E-51 |
| ENSMUSG00000025422 | Agap2 | 23.78 | 9.57E-24 | 5.2 | 9.03E-07 | 4.25 | 2.83E-05 |
| ENSMUSG00000066224 | Arid3c | 23.49 | 5.52E-10 | 372.61 | 1.80E-63 | 32.53 | 2.36E-14 |
| ENSMUSG00000061718 | Ppp1r1b | 23.14 | 1.50E-10 | 14.49 | 3.34E-08 | 3.85 | 0.011208948 |
| ENSMUSG00000089829 | Gm16565 | 23.04 | 9.86E-15 | 6.69 | 4.21E-05 | 4.61 | 0.004075454 |
| ENSMUSG00000038370 | Pcp4l1 | 22.97 | 1.60E-29 | 3.07 | 0.001231964 | 2.92 | 0.004037926 |
| ENSMUSG00000021604 | Irx4 | 22.91 | 2.53E-09 | 27.74 | 1.05E-11 | 13.8 | 3.46E-06 |
| ENSMUSG00000062859 | Tcp11 | 22.81 | 5.03E-20 | 30 | 1.16E-25 | 13.28 | 1.38E-12 |
| ENSMUSG00000056174 | Col8a2 | 22.52 | 2.27E-23 | 82.53 | 5.36E-54 | 4.87 | 0.000167528 |
| ENSMUSG00000000805 | Car4 | 22.44 | 7.21E-10 | 26.24 | 1.32E-10 | 11.07 | 2.03E-06 |
| ENSMUSG00000069227 | Gprin1 | 22.36 | 1.07E-08 | 22.91 | 6.24E-09 | 11.49 | 7.26E-06 |
| ENSMUSG00000027168 | Pax6 | 21.84 | 1.13E-16 | 156.03 | 2.83E-35 | 42.26 | 1.41E-22 |
| ENSMUSG00000075478 | Slitrk1 | 21.83 | 7.44E-06 | 38.95 | 7.32E-09 | 11.35 | 0.004220841 |
| ENSMUSG00000016346 | Kcnq2 | 21.81 | 1.33E-21 | 6.35 | 6.35E-08 | 2.6 | 0.031112024 |
| ENSMUSG00000054252 | Fgfr3 | 21.79 | 2.74E-45 | 44.28 | 1.40E-63 | 10.94 | 1.27E-29 |
| ENSMUSG00000091002 | Tcerg1l | 21.67 | 1.85E-24 | 95.16 | 3.54E-62 | 29.19 | 3.88E-32 |
| ENSMUSG00000054728 | Phactr1 | 20.97 | 1.30E-41 | 11.9 | 3.14E-27 | 8.25 | 1.32E-18 |
| ENSMUSG00000055368 | Slc6a2 | 20.73 | 0.003255905 | 15.55 | 0.009006273 | 14.35 | 0.02460384 |
| ENSMUSG00000029778 | Adcyap1r1 | 20.62 | 3.08E-30 | 23.94 | 3.53E-33 | 4.28 | 3.66E-08 |
| ENSMUSG00000020701 | Tmem132e | 20.54 | 0.014658169 | 112.83 | 0.000368552 | 64.59 | 0.001650593 |
| ENSMUSG00000015981 | Stk32c | 20.5 | 2.84E-29 | 18.11 | 1.47E-27 | 13.22 | 1.32E-21 |
| ENSMUSG00000004892 | Bcan | 20.38 | 2.48E-22 | 19.12 | 1.93E-21 | 6.37 | 2.25E-06 |
| ENSMUSG00000020044 | Timp3 | 20.14 | 5.79E-30 | 19.58 | 4.89E-30 | 6.59 | 5.95E-14 |
| ENSMUSG00000039385 | Cdh6 | 20.04 | 5.62E-25 | 43.37 | 2.88E-36 | 18.75 | 7.92E-24 |
| ENSMUSG00000021365 | Nedd9 | 19.88 | 2.97E-11 | 13.47 | 4.40E-09 | 8.13 | 2.43E-06 |
| ENSMUSG00000020598 | Nrcam | 19.75 | 1.71E-29 | 61.35 | 9.06E-49 | 14.22 | 2.43E-24 |
| ENSMUSG00000022061 | Nkx3-1 | 19.57 | 2.23E-06 | 9.81 | 0.000342634 | 219.29 | 2.83E-14 |
| ENSMUSG00000070576 | Mn1 | 19.36 | 9.15E-21 | 9.79 | 7.49E-14 | 5.82 | 8.41E-09 |
| ENSMUSG00000042129 | Rassf4 | 19.16 | 1.26E-24 | 23 | 1.22E-27 | 5.63 | 1.33E-09 |
| ENSMUSG00000053475 | Tnfaip6 | 19.14 | 1.18E-07 | 6.07 | 0.002623153 | 11.48 | 1.50E-05 |
| ENSMUSG00000063063 | Ctnna2 | 19.03 | 2.34E-23 | 4.45 | 2.71E-07 | 2.64 | 0.002366869 |
| ENSMUSG00000020866 | Cacna1g | 18.63 | 5.20E-72 | 20.54 | 2.65E-76 | 3.84 | 3.68E-18 |
| ENSMUSG00000032564 | Cpne4 | 18.57 | 2.59E-05 | 22.49 | 1.42E-07 | 18.94 | 4.18E-06 |
| ENSMUSG00000030257 | Srgap3 | 18.54 | 7.19E-12 | 20.58 | 1.67E-12 | 7.33 | 2.36E-06 |
| ENSMUSG00000044365 | Cxxc4 | 18.54 | 6.65E-17 | 5.68 | 1.40E-07 | 7.54 | 1.80E-09 |
| ENSMUSG00000083679 | Gm12892 | 18.29 | 5.29E-17 | 28.46 | 5.82E-21 | 5.8 | 2.50E-07 |
| ENSMUSG00000042671 | Rgs8 | 18.27 | 7.60E-20 | 18.01 | 2.06E-19 | 16.16 | 3.77E-18 |
| ENSMUSG00000020732 | Rab37 | 18.17 | 1.95E-24 | 27.2 | 1.55E-30 | 61.5 | 9.25E-44 |
| ENSMUSG00000031760 | Mt3 | 18.04 | 6.77E-19 | 25.65 | 9.51E-26 | 4.37 | 0.002142948 |
| ENSMUSG00000041708 | Mpped1 | 17.93 | 5.15E-60 | 7.5 | 2.22E-31 | 5.82 | 5.79E-24 |
| ENSMUSG00000054320 | Lrrc36 | 17.93 | 4.72E-07 | 29.75 | 4.64E-10 | 5.44 | 0.014449204 |
| ENSMUSG00000034891 | Sncb | 17.54 | 5.40E-09 | 25.87 | 4.86E-11 | 19.93 | 1.52E-09 |
| ENSMUSG00000026866 | Kynu | 17.37 | 0.000337282 | 35.28 | 1.11E-05 | 8.97 | 0.008285739 |
| ENSMUSG00000028373 | Astn2 | 17.2 | 1.63E-20 | 18.81 | 9.32E-23 | 10.25 | 5.71E-12 |
| ENSMUSG00000020297 | Nsg2 | 17.08 | 2.72E-45 | 70.27 | 8.10E-87 | 16.3 | 2.57E-44 |
| ENSMUSG00000044708 | Kcnj10 | 17 | 2.83E-16 | 18.6 | 3.46E-17 | 6.96 | 3.94E-07 |
| ENSMUSG00000024302 | Dtna | 16.68 | 2.78E-68 | 13.11 | 2.48E-59 | 3.54 | 3.46E-16 |
| ENSMUSG00000086291 | Gm15513 | 16.55 | 2.66E-09 | 27 | 2.59E-14 | 12.54 | 5.26E-07 |
| ENSMUSG00000020122 | Egfr | 16.45 | 4.35E-64 | 17.7 | 3.53E-67 | 3.96 | 4.86E-17 |
| ENSMUSG00000031284 | Pak3 | 16.44 | 7.97E-32 | 2.45 | 0.0002048 | 2.44 | 0.000331241 |
| ENSMUSG00000032118 | Fez1 | 16.07 | 3.30E-11 | 13.88 | 3.81E-10 | 7.3 | 1.92E-06 |
| ENSMUSG00000085603 | Gm11346 | 15.93 | 2.69E-09 | 7.2 | 9.57E-05 | 12.15 | 8.04E-08 |
| ENSMUSG00000034730 | Bai1 | 15.84 | 4.76E-22 | 28.63 | 1.62E-30 | 36.34 | 8.98E-34 |
| ENSMUSG00000039405 | Prss23 | 15.81 | 2.28E-14 | 15.28 | 2.02E-14 | 11.36 | 1.46E-11 |
| ENSMUSG00000061815 | Rufy4 | 15.74 | 0.037304328 | 195.44 | 1.15E-09 | 69.53 | 7.66E-06 |
| ENSMUSG00000063919 | Srrm4 | 15.71 | 7.36E-07 | 12.74 | 3.34E-06 | 12.09 | 6.49E-06 |
| ENSMUSG00000013367 | Iglon5 | 15.69 | 9.40E-50 | 6.43 | 3.28E-23 | 4.95 | 1.33E-16 |
| ENSMUSG00000021388 | Aspn | 15.63 | 3.15E-06 | 13.35 | 2.88E-06 | 4.6 | 0.029891147 |
| ENSMUSG00000002341 | Ncan | 15.6 | 3.10E-62 | 5.03 | 1.15E-23 | 2.78 | 6.03E-10 |
| ENSMUSG00000064325 | Hhip | 15.51 | 3.79E-11 | 20.18 | 4.23E-13 | 48.04 | 1.71E-19 |
| ENSMUSG00000046634 | Pkd1l1 | 15.19 | 4.43E-06 | 42.81 | 2.47E-10 | 16.34 | 2.03E-06 |
| ENSMUSG00000042514 | Klhl14 | 15.17 | 0.000326312 | 10.98 | 0.001676536 | 10.84 | 0.002340272 |
| ENSMUSG00000005338 | Cadm3 | 15.08 | 3.22E-21 | 6.08 | 3.51E-10 | 8.55 | 1.10E-13 |
| ENSMUSG00000036006 | Fam65b | 14.88 | 1.01E-05 | 52.53 | 7.65E-12 | 27.9 | 2.01E-08 |
| ENSMUSG00000018427 | Ypel2 | 14.7 | 2.48E-32 | 10.39 | 3.84E-26 | 9.71 | 1.91E-24 |
| ENSMUSG00000047798 | Cd300lf | 14.63 | 5.92E-05 | 6.09 | 0.047731897 | 15.98 | 9.51E-05 |
| ENSMUSG00000089916 | Gm16160 | 14.55 | 0.044687997 | 24.23 | 0.007321729 | 25.34 | 0.006776872 |
| ENSMUSG00000024502 | Jakmip2 | 14.53 | 7.46E-54 | 6.15 | 2.98E-27 | 2.23 | 1.05E-05 |
| ENSMUSG00000049939 | Lrrc4 | 14.36 | 3.36E-09 | 51.46 | 1.19E-17 | 20.22 | 2.54E-11 |
| ENSMUSG00000087228 | Gm12827 | 14.33 | 2.72E-06 | 8.26 | 0.000633595 | 7.25 | 0.002892413 |
| ENSMUSG00000097756 | AC169129.1 | 14.28 | 4.34E-10 | 9.44 | 3.12E-07 | 4.6 | 0.005839669 |
| ENSMUSG00000041423 | Paqr6 | 14.25 | 3.89E-15 | 13.98 | 9.80E-15 | 4.03 | 0.000427256 |
| ENSMUSG00000045179 | Sox3 | 14.17 | 5.15E-26 | 18.05 | 2.24E-30 | 12.42 | 5.64E-24 |
| ENSMUSG00000034818 | Celf5 | 14.07 | 4.20E-51 | 2.71 | 4.47E-08 | 2.12 | 0.000109712 |
| ENSMUSG00000017897 | Eya2 | 14.06 | 4.26E-35 | 4.93 | 4.40E-13 | 6.31 | 1.36E-17 |
| ENSMUSG00000053137 | Mapk11 | 13.96 | 6.97E-57 | 11.17 | 2.62E-49 | 4.72 | 2.73E-21 |
| ENSMUSG00000030350 | Prmt8 | 13.9 | 4.07E-11 | 14.27 | 2.18E-11 | 12.8 | 1.77E-10 |
| ENSMUSG00000041468 | Gpr12 | 13.9 | 0.004542928 | 103.21 | 1.30E-07 | 55.13 | 6.41E-06 |
| ENSMUSG00000031548 | Sfrp1 | 13.89 | 5.95E-26 | 15.67 | 1.89E-28 | 3.13 | 2.45E-06 |
| ENSMUSG00000050132 | Sarm1 | 13.82 | 5.70E-17 | 3.37 | 0.000132918 | 3.12 | 0.000449062 |
| ENSMUSG00000031075 | Ano1 | 13.8 | 1.54E-07 | 18.85 | 4.68E-09 | 22.98 | 9.78E-10 |
| ENSMUSG00000045589 | Frrs1l | 13.74 | 2.59E-05 | 5.71 | 0.006135926 | 7.03 | 0.002494034 |
| ENSMUSG00000074303 | Gm10664 | 13.61 | 4.40E-21 | 9.32 | 4.99E-17 | 10.57 | 2.56E-18 |
| ENSMUSG00000028546 | Elavl4 | 13.55 | 3.67E-08 | 5.83 | 0.000132512 | 2.89 | 0.038436417 |
| ENSMUSG00000086596 | Susd5 | 13.53 | 2.02E-29 | 14.84 | 4.81E-33 | 4.38 | 2.71E-08 |
| ENSMUSG00000030075 | Cntn3 | 13.49 | 9.31E-21 | 7.15 | 1.73E-12 | 3.29 | 0.000133028 |
| ENSMUSG00000022512 | Cldn1 | 13.26 | 2.24E-08 | 6.44 | 8.26E-05 | 77.46 | 1.03E-20 |
| ENSMUSG00000020264 | Slc36a2 | 13.15 | 0.000991294 | 43.07 | 4.45E-08 | 60.09 | 1.35E-09 |
| ENSMUSG00000024109 | Nrxn1 | 13.06 | 3.29E-21 | 6.08 | 5.04E-11 | 3.79 | 1.13E-05 |
| ENSMUSG00000029120 | Ppp2r2c | 13.04 | 4.26E-35 | 7.37 | 7.62E-23 | 4.36 | 1.14E-12 |
| ENSMUSG00000039913 | Pak7 | 13.03 | 2.52E-15 | 2.89 | 0.010705622 | 3.58 | 0.000881349 |
| ENSMUSG00000042631 | Xkr7 | 12.77 | 1.29E-05 | 33.93 | 5.69E-10 | 19.35 | 1.96E-07 |
| ENSMUSG00000074785 | Plxnc1 | 12.75 | 4.92E-11 | 12.33 | 4.99E-11 | 3.94 | 0.000550826 |
| ENSMUSG00000025582 | Nptx1 | 12.69 | 3.53E-13 | 36.81 | 1.14E-23 | 18.7 | 9.01E-17 |
| ENSMUSG00000059049 | Frem1 | 12.55 | 3.72E-09 | 11.93 | 7.57E-09 | 9.43 | 1.82E-07 |
| ENSMUSG00000036586 | Grifin | 12.21 | 0.048744924 | 855.79 | 6.59E-09 | 57.39 | 0.000582132 |
| ENSMUSG00000023473 | Celsr3 | 12.06 | 1.76E-43 | 11.3 | 3.18E-42 | 4.41 | 1.61E-15 |
| ENSMUSG00000046834 | Krt1 | 12 | 0.000186166 | 11.86 | 0.000106996 | 8.46 | 0.004092297 |
| ENSMUSG00000020173 | Cobl | 11.96 | 6.08E-13 | 11.43 | 1.20E-12 | 6.43 | 4.30E-08 |
| ENSMUSG00000087498 | 2210416O15Rik | 11.88 | 1.48E-16 | 4.46 | 9.69E-07 | 8.85 | 5.94E-13 |
| ENSMUSG00000043668 | Tox3 | 11.87 | 1.66E-24 | 9.09 | 3.55E-21 | 3.58 | 5.76E-08 |
| ENSMUSG00000034701 | Neurod1 | 11.85 | 1.30E-12 | 6.15 | 1.37E-07 | 5.68 | 8.51E-07 |
| ENSMUSG00000055069 | Rab39 | 11.83 | 2.10E-17 | 8.26 | 1.01E-13 | 5.78 | 2.83E-09 |
| ENSMUSG00000003411 | Rab3b | 11.8 | 5.63E-26 | 7.29 | 8.91E-17 | 3.94 | 1.88E-07 |
| ENSMUSG00000028370 | Pappa | 11.76 | 1.39E-14 | 29.43 | 2.35E-24 | 14.8 | 5.43E-17 |
| ENSMUSG00000002190 | Clgn | 11.68 | 2.93E-25 | 23.59 | 3.17E-47 | 7.58 | 2.33E-17 |
| ENSMUSG00000039628 | Hs3st6 | 11.6 | 1.51E-05 | 7.69 | 0.001873823 | 15.13 | 1.10E-06 |
| ENSMUSG00000022206 | Npr3 | 11.55 | 8.22E-39 | 2.7 | 1.27E-06 | 5.27 | 2.96E-18 |
| ENSMUSG00000075334 | Rprm | 11.54 | 2.54E-16 | 8.84 | 2.24E-13 | 4.01 | 4.82E-06 |
| ENSMUSG00000031785 | Gpr56 | 11.26 | 8.10E-27 | 16.26 | 6.89E-34 | 2.72 | 1.29E-05 |
| ENSMUSG00000086166 | Gm14342 | 11.23 | 7.66E-19 | 6.91 | 1.21E-11 | 5.53 | 1.24E-08 |
| ENSMUSG00000020101 | 4632428N05Rik | 11.22 | 1.10E-23 | 25.9 | 5.70E-38 | 16.48 | 1.13E-29 |
| ENSMUSG00000032303 | Chrna3 | 11.18 | 9.71E-05 | 42.21 | 7.78E-10 | 10.01 | 0.000209747 |
| ENSMUSG00000031160 | Eras | 11.12 | 3.22E-07 | 11.58 | 1.45E-07 | 6.33 | 0.000213372 |
| ENSMUSG00000025757 | Hspa4l | 11.05 | 1.06E-53 | 10.54 | 5.24E-53 | 3.54 | 6.68E-17 |
| ENSMUSG00000027849 | Syt6 | 11.05 | 2.27E-25 | 10.4 | 3.18E-24 | 8.07 | 1.05E-18 |
| ENSMUSG00000054855 | Rnd1 | 10.87 | 3.58E-30 | 7.86 | 8.55E-23 | 5.47 | 9.29E-15 |
| ENSMUSG00000047013 | Fbxo41 | 10.84 | 1.67E-20 | 6.15 | 2.14E-11 | 3.59 | 5.36E-05 |
| ENSMUSG00000067199 | Frat1 | 10.71 | 4.81E-11 | 5.38 | 1.70E-05 | 2.93 | 0.031151053 |
| ENSMUSG00000041559 | Fmod | 10.68 | 0.026213903 | 8.59 | 0.036524032 | 23.05 | 0.000109627 |
| ENSMUSG00000000567 | Sox9 | 10.64 | 9.77E-14 | 4.07 | 7.16E-06 | 12.94 | 3.59E-16 |
| ENSMUSG00000029660 | Tex26 | 10.62 | 6.03E-33 | 5.7 | 1.41E-18 | 3.55 | 1.67E-09 |
| ENSMUSG00000074657 | Kif5a | 10.58 | 5.09E-34 | 20.53 | 2.04E-54 | 2.96 | 1.03E-07 |
| ENSMUSG00000079157 | Fam155a | 10.48 | 2.76E-19 | 3.03 | 0.000186122 | 3.61 | 1.84E-05 |
| ENSMUSG00000078234 | Klhdc7a | 10.45 | 1.60E-14 | 13.83 | 5.42E-17 | 4.99 | 2.55E-07 |
| ENSMUSG00000090291 | Lrrc10b | 10.31 | 2.15E-07 | 20.58 | 7.42E-13 | 17.88 | 1.71E-11 |
| ENSMUSG00000054074 | Skida1 | 10.24 | 5.61E-13 | 5.72 | 2.85E-08 | 2.7 | 0.00358276 |
| ENSMUSG00000047604 | Frat2 | 10.07 | 4.34E-29 | 7.22 | 1.34E-22 | 5.76 | 1.61E-17 |
| ENSMUSG00000068859 | Sp9 | 10.07 | 0.000203451 | 5.83 | 0.007722699 | 4.33 | 0.048492 |
| ENSMUSG00000024500 | Ppp2r2b | 10.02 | 3.17E-10 | 37.71 | 4.29E-26 | 6.92 | 2.52E-07 |
| ENSMUSG00000095105 | Edaradd | 9.89 | 0.00825597 | 9.1 | 0.014314179 | 11.48 | 0.007010038 |
| ENSMUSG00000036098 | Myrf | 9.88 | 3.75E-21 | 20.07 | 6.46E-33 | 8.21 | 2.96E-18 |
| ENSMUSG00000034336 | Ina | 9.86 | 1.91E-27 | 6.42 | 4.40E-20 | 2.03 | 0.001879182 |
| ENSMUSG00000038065 | 2410066E13Rik | 9.68 | 1.84E-21 | 7.23 | 5.22E-17 | 4.5 | 1.27E-09 |
| ENSMUSG00000010086 | Rnf112 | 9.64 | 7.61E-09 | 6.4 | 1.43E-05 | 5.4 | 0.000357946 |
| ENSMUSG00000043518 | Rai2 | 9.6 | 2.11E-09 | 15.1 | 7.15E-13 | 3.66 | 0.002428574 |
| ENSMUSG00000022738 | Gsc2 | 9.54 | 7.99E-06 | 4.26 | 0.027357702 | 4.92 | 0.012014262 |
| ENSMUSG00000049999 | Ppp1r3d | 9.54 | 3.95E-12 | 6.26 | 3.52E-08 | 7.78 | 2.96E-10 |
| ENSMUSG00000057897 | Camk2b | 9.54 | 1.11E-14 | 6.71 | 3.92E-11 | 3.69 | 1.22E-05 |
| ENSMUSG00000025407 | Gli1 | 9.52 | 1.30E-29 | 17.86 | 3.83E-45 | 18.63 | 2.50E-46 |
| ENSMUSG00000024743 | Syt7 | 9.47 | 9.20E-46 | 12.02 | 7.46E-56 | 5.56 | 7.63E-29 |
| ENSMUSG00000026188 | Tmem169 | 9.47 | 3.27E-16 | 4.35 | 1.23E-06 | 8.07 | 5.30E-14 |
| ENSMUSG00000027330 | Cdc25b | 9.47 | 1.09E-49 | 10.67 | 6.96E-54 | 5.56 | 1.54E-30 |
| ENSMUSG00000021750 | Fam107a | 9.46 | 0.004815419 | 14.77 | 0.000213907 | 9.49 | 0.005768506 |
| ENSMUSG00000042078 | Svop | 9.44 | 6.45E-20 | 2.51 | 0.000399719 | 2.15 | 0.005325649 |
| ENSMUSG00000005958 | Ephb3 | 9.41 | 1.37E-48 | 6.32 | 2.69E-34 | 7.38 | 1.68E-39 |
| ENSMUSG00000043670 | Diras1 | 9.37 | 1.21E-14 | 8.18 | 6.34E-14 | 5.43 | 3.08E-08 |
| ENSMUSG00000037754 | Ppp1r16b | 9.34 | 1.40E-44 | 8.74 | 2.26E-42 | 2.17 | 5.42E-06 |
| ENSMUSG00000035551 | Igfbpl1 | 9.32 | 9.54E-40 | 15.45 | 2.35E-59 | 9.34 | 9.17E-41 |
| ENSMUSG00000010066 | Cacna2d2 | 9.31 | 1.98E-32 | 7.75 | 3.22E-28 | 3.72 | 9.68E-12 |
| ENSMUSG00000056553 | Ptprn2 | 9.3 | 1.46E-06 | 12.06 | 9.10E-08 | 3.52 | 0.010584467 |
| ENSMUSG00000026873 | Phf19 | 9.17 | 8.38E-33 | 11.84 | 2.09E-41 | 7.37 | 5.14E-27 |
| ENSMUSG00000036545 | Adamts2 | 9.16 | 1.87E-32 | 3.89 | 1.38E-12 | 2.71 | 8.57E-07 |
| ENSMUSG00000049775 | Tmsb4x | 9.15 | 4.42E-13 | 2.87 | 0.000608557 | 3.08 | 0.000364766 |
| ENSMUSG00000025128 | Bhlhe22 | 9.08 | 1.07E-10 | 4.11 | 3.57E-05 | 3.55 | 0.000426945 |
| ENSMUSG00000020135 | Apc2 | 9.07 | 2.32E-30 | 7.92 | 1.75E-27 | 2.79 | 3.15E-07 |
| ENSMUSG00000022790 | Igsf11 | 9.07 | 1.39E-16 | 137.14 | 4.56E-69 | 4.64 | 4.89E-08 |
| ENSMUSG00000027848 | Olfml3 | 9.05 | 8.58E-12 | 15.53 | 2.02E-16 | 7.95 | 3.25E-10 |
| ENSMUSG00000039057 | Myo16 | 9.02 | 2.02E-07 | 8.37 | 9.46E-07 | 4.09 | 0.001991286 |
| ENSMUSG00000043463 | Rab9b | 8.99 | 0.010759335 | 7.52 | 0.017039664 | 14.64 | 0.000234923 |
| ENSMUSG00000019989 | Enpp3 | 8.98 | 8.71E-09 | 15.99 | 8.34E-13 | 7.13 | 2.87E-07 |
| ENSMUSG00000050967 | Creg2 | 8.89 | 1.64E-11 | 6.24 | 1.38E-07 | 5.6 | 1.14E-06 |
| ENSMUSG00000027579 | Srms | 8.88 | 0.008465345 | 14.92 | 3.02E-05 | 6.31 | 0.047856144 |
| ENSMUSG00000046618 | Olfml2a | 8.88 | 1.91E-27 | 16.72 | 9.54E-43 | 10.85 | 4.91E-32 |
| ENSMUSG00000001827 | Folr1 | 8.87 | 1.54E-11 | 21.23 | 1.37E-22 | 5.53 | 3.82E-07 |
| ENSMUSG00000042784 | Muc1 | 8.82 | 2.24E-10 | 12.67 | 1.51E-13 | 5.74 | 6.10E-07 |
| ENSMUSG00000000732 | Icosl | 8.7 | 6.34E-32 | 11.24 | 1.70E-40 | 6.44 | 6.44E-24 |
| ENSMUSG00000049690 | Nckap5 | 8.7 | 1.31E-14 | 8 | 9.40E-14 | 4.88 | 7.90E-08 |
| ENSMUSG00000000305 | Cdh4 | 8.67 | 1.52E-21 | 14.64 | 1.73E-31 | 6.12 | 1.32E-15 |
| ENSMUSG00000038602 | Slc35f1 | 8.64 | 1.00E-16 | 7.37 | 3.65E-15 | 2.53 | 0.000715506 |
| ENSMUSG00000042002 | Foxn4 | 8.64 | 4.88E-08 | 21.69 | 1.64E-19 | 10.16 | 2.61E-09 |
| ENSMUSG00000004347 | Pde1c | 8.63 | 1.94E-24 | 2.62 | 2.24E-05 | 2.97 | 1.87E-06 |
| ENSMUSG00000017692 | Rhbdl3 | 8.62 | 4.48E-06 | 23.56 | 5.72E-11 | 29.07 | 4.35E-12 |
| ENSMUSG00000025375 | Aatk | 8.59 | 6.37E-26 | 9.82 | 1.30E-28 | 5.65 | 1.03E-16 |
| ENSMUSG00000029861 | Fam131b | 8.57 | 6.66E-34 | 4.44 | 7.53E-17 | 3.8 | 1.75E-13 |
| ENSMUSG00000039037 | St6galnac5 | 8.57 | 4.06E-12 | 6.78 | 4.92E-10 | 11.69 | 8.68E-16 |
| ENSMUSG00000015850 | Adamtsl4 | 8.56 | 1.02E-19 | 13.42 | 2.69E-28 | 6.84 | 7.08E-15 |
| ENSMUSG00000033578 | Tmem35 | 8.53 | 6.73E-22 | 8.71 | 3.38E-23 | 3.17 | 4.61E-06 |
| ENSMUSG00000053279 | Aldh1a1 | 8.52 | 0.000554593 | 8.76 | 0.000626745 | 7.25 | 0.002692982 |
| ENSMUSG00000064293 | Cntn4 | 8.49 | 0.015694569 | 8.85 | 0.018803908 | 15.06 | 0.001045566 |
| ENSMUSG00000070942 | Il1rl2 | 8.47 | 0.017702869 | 7.79 | 0.043368698 | 10.74 | 0.010602093 |
| ENSMUSG00000045287 | Rtn4rl1 | 8.43 | 3.28E-08 | 16.33 | 3.15E-13 | 4.08 | 0.000634614 |
| ENSMUSG00000068617 | Efcab1 | 8.43 | 1.09E-23 | 2.86 | 8.71E-07 | 4.04 | 5.69E-11 |
| ENSMUSG00000024935 | Slc1a1 | 8.41 | 2.35E-08 | 6.75 | 3.17E-07 | 3.03 | 0.024863115 |
| ENSMUSG00000025588 | Nat1 | 8.34 | 5.86E-08 | 4.21 | 0.000608289 | 4.51 | 0.000382673 |
| ENSMUSG00000049555 | Tmie | 8.31 | 0.005877666 | 12.66 | 0.000672232 | 9.37 | 0.004732279 |
| ENSMUSG00000026587 | Astn1 | 8.29 | 2.76E-32 | 7.53 | 3.86E-30 | 3.99 | 5.61E-14 |
| ENSMUSG00000027560 | Dok5 | 8.21 | 2.07E-13 | 5.51 | 2.78E-09 | 9.18 | 3.86E-15 |
| ENSMUSG00000055235 | Wdr86 | 8.08 | 3.92E-10 | 19.41 | 6.78E-18 | 8.29 | 4.78E-10 |
| ENSMUSG00000027938 | Creb3l4 | 8.03 | 3.90E-22 | 4.46 | 8.23E-12 | 3.58 | 2.94E-08 |
| ENSMUSG00000066170 | E230001N04Rik | 8.03 | 2.48E-05 | 5.73 | 0.000379885 | 5.93 | 0.000391603 |
| ENSMUSG00000021850 | 1700011H14Rik | 8.02 | 0.00181007 | 747.5 | 3.74E-23 | 13.72 | 6.69E-05 |
| ENSMUSG00000019124 | Scrn1 | 7.97 | 1.77E-17 | 5.32 | 3.49E-12 | 2.16 | 0.004335472 |
| ENSMUSG00000005640 | Insrr | 7.95 | 0.003640173 | 7.16 | 0.008399888 | 42.91 | 1.22E-12 |
| ENSMUSG00000020331 | Hcn2 | 7.94 | 6.41E-23 | 5.76 | 6.47E-17 | 3.15 | 2.30E-07 |
| ENSMUSG00000058620 | Adra2b | 7.94 | 8.38E-15 | 11.48 | 1.70E-21 | 8.54 | 4.64E-16 |
| ENSMUSG00000040812 | Agbl2 | 7.88 | 1.33E-22 | 4.03 | 9.35E-11 | 4.06 | 2.14E-10 |
| ENSMUSG00000037224 | Zfyve28 | 7.85 | 2.00E-09 | 20.05 | 6.45E-19 | 5.78 | 4.01E-07 |
| ENSMUSG00000045532 | C1ql1 | 7.82 | 5.56E-25 | 4.64 | 1.04E-14 | 2.77 | 1.30E-06 |
| ENSMUSG00000033453 | Adamts15 | 7.78 | 5.42E-25 | 10.88 | 6.07E-35 | 14.05 | 4.69E-43 |
| ENSMUSG00000037625 | Cldn11 | 7.76 | 8.67E-16 | 3.57 | 2.09E-06 | 18.74 | 2.67E-29 |
| ENSMUSG00000085399 | 9130206I24Rik | 7.76 | 2.06E-12 | 6.04 | 1.09E-09 | 57.97 | 1.67E-42 |
| ENSMUSG00000037379 | Spon2 | 7.75 | 1.42E-13 | 9.33 | 6.84E-16 | 6.38 | 1.51E-10 |
| ENSMUSG00000058420 | Syt17 | 7.72 | 1.74E-10 | 5.98 | 6.13E-08 | 3.04 | 0.002817593 |
| ENSMUSG00000060969 | Irx1 | 7.67 | 9.06E-16 | 8.57 | 2.07E-17 | 3.81 | 1.80E-07 |
| ENSMUSG00000038473 | Nos1ap | 7.65 | 1.48E-14 | 18.7 | 5.91E-31 | 7.93 | 3.79E-15 |
| ENSMUSG00000068740 | Celsr2 | 7.62 | 2.45E-10 | 9.89 | 1.03E-12 | 2.31 | 0.018085481 |
| ENSMUSG00000062380 | Tubb3 | 7.57 | 1.24E-20 | 5.51 | 1.34E-15 | 2.84 | 1.69E-06 |
| ENSMUSG00000022894 | Adamts5 | 7.56 | 3.93E-08 | 6.37 | 2.35E-07 | 4.83 | 2.44E-05 |
| ENSMUSG00000026442 | Nfasc | 7.55 | 6.79E-37 | 6.78 | 5.60E-34 | 2.15 | 4.87E-06 |
| ENSMUSG00000001240 | Ramp2 | 7.53 | 2.22E-18 | 5.25 | 6.42E-13 | 7.2 | 3.14E-17 |
| ENSMUSG00000037843 | Vstm2l | 7.48 | 0.001549803 | 5.12 | 0.037447399 | 99.32 | 3.00E-29 |
| ENSMUSG00000023232 | Serinc2 | 7.45 | 4.40E-24 | 6.03 | 6.69E-20 | 6.8 | 3.99E-22 |
| ENSMUSG00000017639 | Rab11fip4 | 7.41 | 5.04E-41 | 5.32 | 6.92E-31 | 2.74 | 3.52E-12 |
| ENSMUSG00000038677 | Scube3 | 7.41 | 2.55E-19 | 10.17 | 6.86E-25 | 7.49 | 2.00E-19 |
| ENSMUSG00000027359 | Slc27a2 | 7.36 | 1.25E-11 | 15.3 | 2.50E-23 | 2.26 | 0.043407412 |
| ENSMUSG00000041046 | Ramp3 | 7.36 | 8.51E-13 | 6.84 | 7.66E-12 | 4.04 | 1.36E-06 |
| ENSMUSG00000045377 | Tmem88 | 7.35 | 3.07E-06 | 5.85 | 3.35E-05 | 3.05 | 0.018161222 |
| ENSMUSG00000087565 | Gm14662 | 7.34 | 2.52E-08 | 7.91 | 1.03E-08 | 8.5 | 3.11E-09 |
| ENSMUSG00000024534 | Sncaip | 7.32 | 1.50E-06 | 6.09 | 1.22E-05 | 20.69 | 4.92E-13 |
| ENSMUSG00000035775 | Krt20 | 7.32 | 0.000419894 | 95.22 | 1.98E-33 | 55.18 | 6.15E-25 |
| ENSMUSG00000039831 | Arhgap29 | 7.32 | 3.78E-36 | 4.31 | 1.12E-21 | 4.04 | 1.39E-19 |
| ENSMUSG00000021215 | Net1 | 7.27 | 1.67E-40 | 7.9 | 5.77E-45 | 4.74 | 8.53E-27 |
| ENSMUSG00000027238 | Frmd5 | 7.23 | 1.57E-37 | 4.84 | 1.12E-25 | 2.59 | 9.10E-10 |
| ENSMUSG00000044024 | Rell2 | 7.22 | 9.25E-12 | 6.57 | 1.50E-10 | 3.15 | 0.0013076 |
| ENSMUSG00000053368 | Rxfp2 | 7.22 | 0.00015314 | 8 | 6.98E-05 | 8.56 | 4.54E-05 |
| ENSMUSG00000057716 | Tmem178b | 7.2 | 4.85E-09 | 15.32 | 1.47E-16 | 4.11 | 7.05E-05 |
| ENSMUSG00000030849 | Fgfr2 | 7.16 | 1.59E-11 | 15.24 | 3.13E-20 | 6.07 | 4.50E-10 |
| ENSMUSG00000035131 | Fam5c | 7.15 | 0.016133389 | 22.69 | 5.26E-07 | 11.73 | 0.000728402 |
| ENSMUSG00000042873 | Lhfpl4 | 7.15 | 3.64E-34 | 5.78 | 1.39E-28 | 3.5 | 4.51E-15 |
| ENSMUSG00000079022 | Col22a1 | 7.13 | 1.86E-36 | 7.45 | 1.03E-37 | 2.93 | 1.09E-11 |
| ENSMUSG00000040972 | Igsf21 | 7.12 | 1.69E-12 | 4.61 | 3.54E-07 | 5.74 | 1.57E-09 |
| ENSMUSG00000028128 | F3 | 7.06 | 7.14E-24 | 4.38 | 4.04E-14 | 2.46 | 1.87E-05 |
| ENSMUSG00000020811 | Wscd1 | 7 | 1.86E-36 | 3.52 | 1.97E-16 | 2.42 | 1.81E-08 |
| ENSMUSG00000036907 | C1ql2 | 7 | 0.028829638 | 66.29 | 1.70E-08 | 53.14 | 1.11E-07 |
| ENSMUSG00000021466 | Ptch1 | 6.99 | 3.92E-15 | 8.12 | 4.11E-17 | 7 | 3.39E-15 |
| ENSMUSG00000022758 | P2rx6 | 6.99 | 9.58E-14 | 7.42 | 1.87E-15 | 3.29 | 4.40E-05 |
| ENSMUSG00000063142 | Kcnma1 | 6.95 | 1.13E-11 | 3.4 | 4.87E-05 | 4.95 | 4.91E-08 |
| ENSMUSG00000020032 | Nuak1 | 6.93 | 5.63E-19 | 5.59 | 6.48E-16 | 2.51 | 3.71E-05 |
| ENSMUSG00000047878 | A4galt | 6.91 | 4.04E-16 | 5.4 | 4.06E-13 | 2.07 | 0.007262561 |
| ENSMUSG00000026679 | Enkur | 6.87 | 0.002689946 | 5.14 | 0.006810944 | 5.79 | 0.004289751 |
| ENSMUSG00000034586 | Hid1 | 6.87 | 4.53E-31 | 4.07 | 1.46E-16 | 5.42 | 2.05E-23 |
| ENSMUSG00000054200 | Ffar4 | 6.86 | 0.004353774 | 18.9 | 2.58E-08 | 12.65 | 1.26E-05 |
| ENSMUSG00000027967 | Neurog2 | 6.82 | 0.015518714 | 11.77 | 0.001895322 | 71.29 | 2.32E-07 |
| ENSMUSG00000034652 | Cd300a | 6.8 | 4.57E-08 | 8.06 | 4.80E-10 | 9.97 | 3.30E-12 |
| ENSMUSG00000052783 | Grk4 | 6.79 | 3.25E-05 | 7.79 | 6.57E-07 | 4.73 | 0.001187741 |
| ENSMUSG00000001507 | Itga3 | 6.78 | 2.18E-37 | 25.58 | 5.83E-91 | 7.31 | 2.04E-39 |
| ENSMUSG00000025170 | Rab40b | 6.77 | 2.14E-10 | 3.13 | 0.000670799 | 4.05 | 1.41E-05 |
| ENSMUSG00000051855 | Mest | 6.77 | 2.33E-25 | 3.31 | 3.98E-11 | 8.25 | 2.52E-30 |
| ENSMUSG00000055027 | Smyd1 | 6.76 | 9.83E-16 | 15.78 | 6.83E-34 | 9.31 | 1.18E-21 |
| ENSMUSG00000031748 | Gnao1 | 6.73 | 2.68E-33 | 4.22 | 2.34E-20 | 3.39 | 6.49E-15 |
| ENSMUSG00000042388 | Dlgap3 | 6.69 | 5.81E-08 | 6.38 | 8.06E-08 | 2.65 | 0.010998768 |
| ENSMUSG00000031465 | Angpt2 | 6.68 | 1.14E-11 | 3.89 | 9.42E-06 | 3.22 | 0.000396186 |
| ENSMUSG00000043013 | Onecut1 | 6.67 | 0.003428711 | 14.3 | 8.77E-06 | 5.43 | 0.010397174 |
| ENSMUSG00000040037 | Negr1 | 6.64 | 7.48E-18 | 3.77 | 2.30E-09 | 2.53 | 0.000212903 |
| ENSMUSG00000054720 | Lrrc8c | 6.64 | 6.87E-25 | 5.04 | 2.77E-19 | 3.14 | 5.36E-10 |
| ENSMUSG00000023236 | Scg5 | 6.56 | 6.86E-22 | 9.59 | 7.72E-32 | 5.29 | 7.64E-17 |
| ENSMUSG00000071636 | Rimbp3 | 6.54 | 2.57E-14 | 6.62 | 2.13E-15 | 4.71 | 8.70E-10 |
| ENSMUSG00000051043 | Gprc5c | 6.52 | 1.80E-21 | 8.88 | 2.59E-28 | 8.49 | 3.77E-27 |
| ENSMUSG00000063531 | Sema3e | 6.52 | 3.17E-33 | 4.77 | 1.32E-25 | 3.87 | 3.75E-19 |
| ENSMUSG00000022208 | Jph4 | 6.49 | 4.63E-21 | 2.4 | 1.16E-05 | 2.19 | 0.000233739 |
| ENSMUSG00000075256 | Cerkl | 6.48 | 0.001095964 | 13.03 | 1.84E-07 | 5.14 | 0.011866767 |
| ENSMUSG00000015094 | Npdc1 | 6.46 | 2.03E-33 | 7.47 | 2.84E-38 | 3.56 | 6.78E-16 |
| ENSMUSG00000046413 | D230002A01Rik | 6.46 | 9.83E-15 | 9.25 | 4.78E-22 | 5.22 | 1.43E-11 |
| ENSMUSG00000053395 | Cacng8 | 6.46 | 2.12E-08 | 3.73 | 0.00033167 | 3.21 | 0.004800435 |
| ENSMUSG00000073207 | Ccdc160 | 6.43 | 5.41E-29 | 8.82 | 5.55E-40 | 4.57 | 7.84E-20 |
| ENSMUSG00000097749 | AC119880.1 | 6.42 | 0.000205948 | 7.76 | 2.45E-05 | 10.13 | 5.63E-07 |
| ENSMUSG00000086742 | Gm16201 | 6.41 | 7.45E-05 | 8.97 | 5.37E-07 | 5.74 | 0.000427165 |
| ENSMUSG00000033615 | Cplx1 | 6.4 | 1.43E-16 | 4.92 | 2.26E-12 | 3.41 | 6.10E-07 |
| ENSMUSG00000047496 | Rnf152 | 6.39 | 5.03E-19 | 4.95 | 9.63E-15 | 3.15 | 7.60E-08 |
| ENSMUSG00000041669 | Prima1 | 6.38 | 0.004490166 | 21.91 | 7.76E-10 | 14.71 | 6.21E-07 |
| ENSMUSG00000032060 | Cryab | 6.36 | 3.86E-09 | 6.49 | 2.09E-09 | 3.45 | 0.000213509 |
| ENSMUSG00000027220 | Syt13 | 6.28 | 1.31E-23 | 8.78 | 1.19E-33 | 3.82 | 5.82E-13 |
| ENSMUSG00000029121 | Crmp1 | 6.26 | 5.63E-35 | 5.06 | 1.30E-28 | 3.81 | 1.11E-19 |
| ENSMUSG00000031539 | Ap3m2 | 6.25 | 1.63E-16 | 6.76 | 3.94E-18 | 2.7 | 1.66E-05 |
| ENSMUSG00000003934 | Efnb3 | 6.2 | 1.14E-11 | 8.39 | 2.77E-15 | 2.88 | 0.000111177 |
| ENSMUSG00000063568 | Jazf1 | 6.2 | 1.44E-14 | 7.13 | 9.30E-19 | 2.73 | 0.000176358 |
| ENSMUSG00000035456 | Prdm8 | 6.14 | 0.017953513 | 5.62 | 0.034500997 | 7.72 | 0.011853049 |
| ENSMUSG00000035517 | Tdrd7 | 6.12 | 3.29E-17 | 5.97 | 5.62E-17 | 4.62 | 8.69E-13 |
| ENSMUSG00000027612 | Mmp24 | 6.11 | 0.000115253 | 7.34 | 8.09E-06 | 4.15 | 0.008096769 |
| ENSMUSG00000044551 | 9930012K11Rik | 6.08 | 8.83E-25 | 7.78 | 3.13E-32 | 4.3 | 2.50E-16 |
| ENSMUSG00000055675 | Kbtbd11 | 6.08 | 7.50E-16 | 9.61 | 3.74E-23 | 3.24 | 2.11E-07 |
| ENSMUSG00000029335 | Bmp3 | 6.01 | 0.006582289 | 9.85 | 0.000390035 | 31.13 | 1.53E-07 |
| ENSMUSG00000072944 | Nup62cl | 5.97 | 1.39E-15 | 2.74 | 9.97E-06 | 2.37 | 0.000449062 |
| ENSMUSG00000047182 | Irs3 | 5.93 | 0.011611765 | 5.69 | 0.015851941 | 6.42 | 0.009811321 |
| ENSMUSG00000057265 | Ccdc176 | 5.93 | 8.83E-16 | 4.2 | 9.57E-11 | 3.6 | 2.02E-08 |
| ENSMUSG00000034771 | Tle2 | 5.92 | 0.0007612 | 4.37 | 0.007153125 | 6.89 | 0.000300202 |
| ENSMUSG00000047497 | Adamts12 | 5.91 | 9.55E-05 | 5.46 | 0.000269581 | 4.89 | 0.000803515 |
| ENSMUSG00000035211 | Xrra1 | 5.89 | 4.68E-11 | 5.06 | 2.13E-09 | 3.47 | 1.85E-05 |
| ENSMUSG00000049551 | Fzd9 | 5.88 | 4.18E-08 | 7.71 | 2.99E-10 | 4.19 | 1.62E-05 |
| ENSMUSG00000028121 | Bcar3 | 5.84 | 1.55E-20 | 5.28 | 2.16E-18 | 3.24 | 1.15E-09 |
| ENSMUSG00000055022 | Cntn1 | 5.84 | 4.40E-12 | 2.81 | 8.67E-05 | 3.17 | 1.21E-05 |
| ENSMUSG00000034981 | Parm1 | 5.82 | 1.48E-15 | 8.51 | 2.20E-22 | 4.54 | 8.24E-12 |
| ENSMUSG00000032224 | Fam81a | 5.81 | 4.00E-12 | 7.49 | 2.64E-16 | 3.11 | 4.53E-05 |
| ENSMUSG00000086851 | A430108G06Rik | 5.78 | 6.29E-06 | 2.58 | 0.042555593 | 2.69 | 0.036555339 |
| ENSMUSG00000001494 | Sost | 5.77 | 0.006606176 | 6.23 | 0.004850556 | 20.82 | 2.06E-06 |
| ENSMUSG00000066113 | Adamtsl1 | 5.74 | 1.10E-10 | 20.99 | 2.12E-29 | 14.88 | 7.60E-24 |
| ENSMUSG00000032878 | Ccdc85a | 5.73 | 1.21E-06 | 7.18 | 2.94E-08 | 4.31 | 7.31E-05 |
| ENSMUSG00000042115 | Klhdc8a | 5.71 | 3.77E-07 | 4.18 | 3.94E-05 | 4.52 | 2.29E-05 |
| ENSMUSG00000022840 | Adcy5 | 5.68 | 1.21E-24 | 4.04 | 3.91E-16 | 5.93 | 3.39E-25 |
| ENSMUSG00000051920 | Rspo2 | 5.66 | 0.000492472 | 6.11 | 0.00046157 | 3.79 | 0.035992246 |
| ENSMUSG00000020601 | Trib2 | 5.63 | 7.98E-16 | 3.9 | 6.54E-11 | 2.02 | 0.001935641 |
| ENSMUSG00000050272 | Dscam | 5.62 | 2.93E-05 | 3.1 | 0.013279657 | 3.67 | 0.003920787 |
| ENSMUSG00000069733 | Ube2u | 5.62 | 0.021063302 | 15.86 | 2.40E-05 | 7.8 | 0.005846659 |
| ENSMUSG00000087143 | A830082K12Rik | 5.62 | 5.87E-05 | 3.17 | 0.009363871 | 6.25 | 1.91E-05 |
| ENSMUSG00000050677 | Ccdc96 | 5.6 | 1.57E-08 | 3.73 | 2.87E-05 | 2.4 | 0.013586647 |
| ENSMUSG00000090257 | Gm4524 | 5.56 | 6.67E-06 | 8.36 | 9.94E-09 | 11.77 | 2.81E-11 |
| ENSMUSG00000027793 | Ccna1 | 5.55 | 4.66E-06 | 11.42 | 2.97E-12 | 8.74 | 1.82E-09 |
| ENSMUSG00000042751 | Nmnat2 | 5.55 | 8.24E-11 | 3 | 4.61E-05 | 2.39 | 0.003079078 |
| ENSMUSG00000051279 | Gdf6 | 5.54 | 0.000131732 | 8.33 | 1.19E-06 | 3.32 | 0.01931132 |
| ENSMUSG00000015599 | Ttbk1 | 5.51 | 2.00E-12 | 3.11 | 3.17E-05 | 2.05 | 0.036719932 |
| ENSMUSG00000044197 | Gpr146 | 5.5 | 3.15E-08 | 17.07 | 1.39E-26 | 5.81 | 1.30E-09 |
| ENSMUSG00000000126 | Wnt9a | 5.49 | 3.87E-11 | 2.69 | 0.000285776 | 2.61 | 0.000561943 |
| ENSMUSG00000021130 | Galnt16 | 5.49 | 8.65E-15 | 7.18 | 3.75E-19 | 3.5 | 1.55E-08 |
| ENSMUSG00000024013 | Fgd2 | 5.49 | 5.20E-12 | 4.89 | 6.31E-10 | 3.35 | 1.10E-05 |
| ENSMUSG00000024873 | Cnih2 | 5.49 | 1.10E-21 | 5.45 | 1.00E-21 | 3.26 | 1.41E-10 |
| ENSMUSG00000040009 | Gnaz | 5.49 | 3.67E-21 | 4.35 | 7.28E-17 | 2.66 | 1.10E-07 |
| ENSMUSG00000026072 | Il1r1 | 5.48 | 0.001279458 | 11.39 | 1.13E-06 | 41.65 | 6.13E-14 |
| ENSMUSG00000046442 | Ppm1e | 5.47 | 1.72E-26 | 3.41 | 4.59E-15 | 2.05 | 1.62E-05 |
| ENSMUSG00000030402 | Ppm1n | 5.46 | 2.29E-10 | 3.6 | 2.73E-06 | 2.36 | 0.008216801 |
| ENSMUSG00000030701 | Plekhb1 | 5.41 | 6.58E-14 | 7.79 | 1.21E-21 | 2.63 | 0.000153444 |
| ENSMUSG00000027577 | Chrna4 | 5.38 | 3.45E-16 | 9.06 | 1.73E-26 | 2.64 | 6.51E-06 |
| ENSMUSG00000071550 | Wdr52 | 5.38 | 4.70E-06 | 6.02 | 2.09E-06 | 3.32 | 0.007379708 |
| ENSMUSG00000031778 | Cx3cl1 | 5.37 | 2.53E-12 | 4.72 | 9.68E-11 | 2.22 | 0.003614771 |
| ENSMUSG00000050199 | Lgr4 | 5.36 | 1.48E-13 | 4.53 | 8.67E-12 | 3.34 | 8.29E-08 |
| ENSMUSG00000050556 | Kcnb1 | 5.34 | 4.04E-16 | 3.4 | 7.15E-09 | 2.93 | 1.26E-06 |
| ENSMUSG00000022105 | Rb1 | 5.33 | 1.35E-28 | 2.24 | 7.58E-08 | 2.07 | 3.39E-06 |
| ENSMUSG00000040434 | Gyltl1b | 5.32 | 4.88E-14 | 10.41 | 5.25E-25 | 2.44 | 0.000187498 |
| ENSMUSG00000021070 | Bdkrb2 | 5.31 | 0.000297697 | 10.73 | 7.22E-08 | 3.36 | 0.022568205 |
| ENSMUSG00000019796 | Lrp11 | 5.29 | 6.92E-19 | 3.96 | 1.32E-13 | 4.11 | 4.04E-14 |
| ENSMUSG00000073422 | H2-Ke6 | 5.28 | 2.58E-08 | 6.24 | 1.86E-10 | 3.03 | 0.001092028 |
| ENSMUSG00000079042 | Trim61 | 5.28 | 0.020269307 | 8.37 | 0.000275387 | 7.51 | 0.001155677 |
| ENSMUSG00000031073 | Fgf15 | 5.27 | 1.06E-05 | 3.59 | 0.000745751 | 5.19 | 1.76E-05 |
| ENSMUSG00000025608 | Podxl | 5.26 | 6.29E-26 | 4.63 | 6.92E-23 | 2.99 | 5.10E-12 |
| ENSMUSG00000021411 | Pxdc1 | 5.25 | 0.000157149 | 3.87 | 0.003074216 | 3.95 | 0.003143028 |
| ENSMUSG00000038168 | Leprel1 | 5.25 | 9.84E-14 | 10.75 | 1.31E-25 | 14.13 | 1.62E-30 |
| ENSMUSG00000024247 | Pkdcc | 5.24 | 1.04E-28 | 7.03 | 3.22E-38 | 9.39 | 3.37E-49 |
| ENSMUSG00000069763 | Tmem100 | 5.23 | 1.62E-10 | 2.94 | 0.000144847 | 6.2 | 4.81E-13 |
| ENSMUSG00000024063 | Lbh | 5.22 | 5.64E-07 | 4.91 | 1.85E-06 | 8.69 | 8.42E-11 |
| ENSMUSG00000024155 | Meiob | 5.22 | 5.08E-06 | 6.76 | 8.92E-09 | 10.04 | 1.36E-12 |
| ENSMUSG00000026934 | Lhx3 | 5.22 | 0.014685015 | 12.79 | 4.38E-06 | 36.35 | 8.05E-13 |
| ENSMUSG00000057880 | Abat | 5.21 | 1.23E-14 | 3.17 | 8.99E-08 | 2.17 | 0.000730875 |
| ENSMUSG00000093910 | Zfp853 | 5.21 | 5.86E-05 | 9.93 | 7.44E-12 | 8.92 | 2.12E-10 |
| ENSMUSG00000058589 | Anks1b | 5.19 | 0.000207585 | 3.52 | 0.006978836 | 3.32 | 0.01393523 |
| ENSMUSG00000025997 | Ikzf2 | 5.17 | 4.76E-21 | 4.27 | 2.30E-18 | 2.6 | 3.30E-08 |
| ENSMUSG00000035735 | Dagla | 5.16 | 7.03E-16 | 7.73 | 9.17E-25 | 7.76 | 8.87E-25 |
| ENSMUSG00000039208 | Metrnl | 5.16 | 5.46E-12 | 14.73 | 1.80E-31 | 3.33 | 3.01E-06 |
| ENSMUSG00000027254 | Map1a | 5.14 | 6.09E-08 | 6.48 | 7.08E-10 | 3.3 | 0.000119747 |
| ENSMUSG00000078444 | Gm10941 | 5.14 | 0.001873752 | 3.61 | 0.033912945 | 6.1 | 0.000602994 |
| ENSMUSG00000033597 | Caskin1 | 5.11 | 4.17E-22 | 6.14 | 2.25E-27 | 2.76 | 4.62E-09 |
| ENSMUSG00000027339 | Rassf2 | 5.09 | 3.02E-10 | 3.83 | 2.13E-07 | 2.79 | 0.000134514 |
| ENSMUSG00000031538 | Plat | 5.09 | 1.66E-05 | 12.22 | 1.24E-10 | 6.11 | 3.16E-06 |
| ENSMUSG00000033768 | Nrxn2 | 5.09 | 1.26E-24 | 3.11 | 4.02E-13 | 2.26 | 6.77E-07 |
| ENSMUSG00000000693 | Loxl3 | 5.08 | 6.05E-21 | 7.79 | 1.79E-32 | 4.05 | 3.03E-15 |
| ENSMUSG00000079017 | Ifi27l2a | 5.08 | 2.61E-06 | 4.55 | 3.16E-05 | 4.26 | 0.000125547 |
| ENSMUSG00000031734 | Irx3 | 5.07 | 0.000624949 | 7.58 | 1.32E-05 | 3.61 | 0.010273273 |
| ENSMUSG00000042763 | Maneal | 5.07 | 2.56E-22 | 4.3 | 8.72E-19 | 2.23 | 6.60E-06 |
| ENSMUSG00000006930 | Hap1 | 5.06 | 1.04E-19 | 5.29 | 1.05E-20 | 2.7 | 6.18E-08 |
| ENSMUSG00000019996 | Map7 | 5.05 | 3.76E-16 | 10.17 | 5.57E-31 | 2.21 | 0.000174096 |
| ENSMUSG00000001300 | Efnb2 | 5.04 | 2.62E-15 | 5.32 | 4.62E-17 | 3.42 | 8.70E-10 |
| ENSMUSG00000092035 | Peg10 | 5.04 | 2.02E-07 | 3.98 | 6.31E-06 | 5.15 | 7.22E-08 |
| ENSMUSG00000029178 | Klf3 | 5.03 | 3.06E-26 | 3.16 | 1.01E-14 | 3.44 | 8.03E-17 |
| ENSMUSG00000035407 | Kank4 | 5 | 6.59E-07 | 8.52 | 2.48E-13 | 7.37 | 3.31E-11 |
| ENSMUSG00000024503 | Spink3 | 4.99 | 1.74E-06 | 14.76 | 1.26E-19 | 6.2 | 8.29E-09 |
| ENSMUSG00000006651 | Aplp1 | 4.97 | 1.12E-19 | 7.23 | 1.86E-28 | 3.22 | 8.58E-11 |
| ENSMUSG00000020679 | Hnf1b | 4.95 | 0.02760906 | 32.34 | 1.18E-06 | 9.33 | 0.001876276 |
| ENSMUSG00000074461 | Gm10699 | 4.95 | 1.91E-15 | 3.14 | 4.83E-08 | 3.12 | 9.44E-08 |
| ENSMUSG00000018169 | Mfng | 4.93 | 7.45E-10 | 4.94 | 1.09E-09 | 3.81 | 4.35E-07 |
| ENSMUSG00000019929 | Dcn | 4.92 | 0.002677733 | 13.9 | 1.96E-07 | 4.96 | 0.002688554 |
| ENSMUSG00000019832 | Rab32 | 4.91 | 5.22E-12 | 5.14 | 1.04E-13 | 2.49 | 0.00041463 |
| ENSMUSG00000037989 | Wnk2 | 4.89 | 4.83E-25 | 5.54 | 6.91E-29 | 2.32 | 1.10E-07 |
| ENSMUSG00000041515 | Irf8 | 4.89 | 2.30E-14 | 3.76 | 7.22E-10 | 3.14 | 1.47E-07 |
| ENSMUSG00000086119 | Gm2415 | 4.88 | 0.038569337 | 7.09 | 0.004562248 | 8.56 | 0.002031353 |
| ENSMUSG00000024033 | Rsph1 | 4.87 | 6.33E-13 | 5.67 | 9.53E-16 | 3.75 | 1.10E-08 |
| ENSMUSG00000028444 | Cntfr | 4.87 | 2.41E-24 | 6.05 | 1.10E-30 | 4.07 | 1.64E-19 |
| ENSMUSG00000010660 | Plcd1 | 4.85 | 9.20E-23 | 7.21 | 1.55E-34 | 12.47 | 7.78E-54 |
| ENSMUSG00000032940 | Rbm11 | 4.85 | 3.02E-05 | 3.27 | 0.001998583 | 2.55 | 0.030176995 |
| ENSMUSG00000048485 | Zbtb8b | 4.82 | 7.96E-18 | 3.16 | 1.17E-09 | 2.24 | 8.81E-05 |
| ENSMUSG00000021390 | Ogn | 4.81 | 6.71E-05 | 10.92 | 9.22E-12 | 9.72 | 2.02E-10 |
| ENSMUSG00000026259 | Ngef | 4.8 | 5.11E-17 | 4.58 | 5.78E-16 | 2.19 | 0.000284158 |
| ENSMUSG00000039982 | Dtx4 | 4.8 | 1.23E-09 | 7.84 | 2.01E-15 | 3.32 | 4.83E-06 |
| ENSMUSG00000029788 | Cpa5 | 4.75 | 0.004336794 | 6.32 | 0.000199923 | 43.26 | 1.64E-19 |
| ENSMUSG00000019066 | Rab3d | 4.74 | 9.11E-20 | 6.02 | 1.41E-26 | 4.33 | 3.68E-18 |
| ENSMUSG00000096603 | Gm7694 | 4.72 | 4.17E-15 | 12.11 | 2.50E-38 | 6.25 | 1.10E-20 |
| ENSMUSG00000030772 | Dkk3 | 4.7 | 8.71E-22 | 5.41 | 1.59E-25 | 5.18 | 2.57E-24 |
| ENSMUSG00000047730 | Fcgbp | 4.69 | 0.001017895 | 6.96 | 8.72E-06 | 6.3 | 7.19E-05 |
| ENSMUSG00000021194 | Chga | 4.68 | 1.30E-10 | 4.27 | 8.01E-10 | 2.54 | 0.00085945 |
| ENSMUSG00000031642 | Sh3rf1 | 4.68 | 3.08E-25 | 3.56 | 3.54E-18 | 2.64 | 6.18E-11 |
| ENSMUSG00000086587 | Gm11837 | 4.67 | 3.38E-06 | 3.46 | 0.000190709 | 3.72 | 9.83E-05 |
| ENSMUSG00000034765 | Dusp5 | 4.66 | 4.44E-10 | 5.81 | 1.29E-13 | 6.83 | 2.23E-15 |
| ENSMUSG00000070476 | Fam217b | 4.66 | 4.97E-15 | 3.41 | 4.56E-10 | 4 | 8.18E-13 |
| ENSMUSG00000020878 | Lrrc46 | 4.65 | 1.07E-15 | 3.87 | 1.81E-12 | 3.07 | 1.57E-08 |
| ENSMUSG00000027978 | Prss12 | 4.65 | 0.005586991 | 12.63 | 5.43E-07 | 5.81 | 0.001057592 |
| ENSMUSG00000028626 | Col9a2 | 4.63 | 2.38E-08 | 4.5 | 5.02E-08 | 2.63 | 0.001097813 |
| ENSMUSG00000039860 | Srrm3 | 4.62 | 1.99E-08 | 3.77 | 4.95E-07 | 2.37 | 0.005674095 |
| ENSMUSG00000022887 | Masp1 | 4.61 | 4.47E-07 | 7.09 | 9.66E-11 | 2.56 | 0.00607984 |
| ENSMUSG00000097666 | AC102195.1 | 4.61 | 4.70E-06 | 3.75 | 5.17E-05 | 4.28 | 8.70E-06 |
| ENSMUSG00000028664 | Ephb2 | 4.6 | 1.07E-25 | 4.51 | 3.90E-25 | 2.17 | 1.56E-07 |
| ENSMUSG00000030283 | St8sia1 | 4.6 | 0.000230117 | 9.29 | 8.65E-08 | 4.14 | 0.000915119 |
| ENSMUSG00000037747 | Phyhipl | 4.6 | 3.01E-16 | 2.78 | 2.37E-08 | 2.97 | 6.26E-09 |
| ENSMUSG00000059493 | Nhs | 4.58 | 8.88E-20 | 3 | 1.69E-11 | 2.65 | 7.10E-09 |
| ENSMUSG00000010154 | Spire2 | 4.56 | 1.96E-08 | 7.56 | 3.35E-14 | 3.25 | 3.18E-05 |
| ENSMUSG00000023805 | Synj2 | 4.55 | 1.69E-13 | 5.56 | 1.62E-18 | 3.14 | 5.06E-08 |
| ENSMUSG00000031351 | Zfp185 | 4.55 | 2.10E-09 | 5.05 | 3.58E-11 | 3.69 | 2.32E-07 |
| ENSMUSG00000041112 | Elmo1 | 4.55 | 7.67E-23 | 3.53 | 5.72E-17 | 2.45 | 6.48E-09 |
| ENSMUSG00000018893 | Mb | 4.54 | 0.02727254 | 11.45 | 2.33E-06 | 4.96 | 0.01997638 |
| ENSMUSG00000001497 | Pax9 | 4.52 | 0.00931564 | 7.69 | 0.000383009 | 39.73 | 1.07E-10 |
| ENSMUSG00000047150 | 1700001C19Rik | 4.52 | 0.006982981 | 5.47 | 0.00064229 | 5.26 | 0.001419761 |
| ENSMUSG00000037031 | Tspan15 | 4.51 | 2.51E-18 | 4.51 | 5.77E-19 | 6.28 | 1.17E-27 |
| ENSMUSG00000020407 | Upp1 | 4.5 | 6.48E-22 | 6.75 | 4.33E-35 | 7.53 | 2.10E-38 |
| ENSMUSG00000002944 | Cd36 | 4.47 | 2.58E-12 | 19.27 | 3.93E-46 | 66.47 | 3.32E-82 |
| ENSMUSG00000024754 | Tmem2 | 4.47 | 2.30E-17 | 4.23 | 1.46E-16 | 2.84 | 4.30E-09 |
| ENSMUSG00000030519 | Apba2 | 4.45 | 2.52E-11 | 9.01 | 2.98E-22 | 3.43 | 7.43E-08 |
| ENSMUSG00000030125 | Lrrc23 | 4.44 | 0.000399558 | 3.01 | 0.012074155 | 3.62 | 0.002363717 |
| ENSMUSG00000038296 | Galnt18 | 4.44 | 2.49E-14 | 8.35 | 6.17E-28 | 14.25 | 3.88E-43 |
| ENSMUSG00000078607 | 1810010H24Rik | 4.44 | 5.35E-12 | 4.82 | 1.10E-13 | 4.62 | 1.73E-12 |
| ENSMUSG00000034574 | Daam1 | 4.43 | 1.31E-09 | 3.6 | 1.10E-07 | 2.15 | 0.003820783 |
| ENSMUSG00000042564 | Fam227a | 4.43 | 6.90E-11 | 3.57 | 1.44E-08 | 3.05 | 1.85E-06 |
| ENSMUSG00000097757 | AC160759.1 | 4.43 | 1.54E-08 | 3.32 | 1.45E-05 | 4.13 | 1.37E-07 |
| ENSMUSG00000051146 | Camk2n2 | 4.42 | 2.98E-15 | 4.36 | 7.33E-15 | 2.4 | 3.17E-05 |
| ENSMUSG00000028064 | Sema4a | 4.41 | 1.63E-10 | 8.01 | 6.56E-20 | 4.23 | 2.59E-09 |
| ENSMUSG00000032482 | Cspg5 | 4.39 | 0.020395489 | 30.74 | 1.42E-07 | 6.74 | 0.004320296 |
| ENSMUSG00000018672 | Copz2 | 4.37 | 7.54E-20 | 3.55 | 4.67E-15 | 4.73 | 1.22E-21 |
| ENSMUSG00000037664 | Cdkn1c | 4.37 | 7.97E-05 | 2.27 | 0.042106369 | 2.38 | 0.038132041 |
| ENSMUSG00000042109 | Csdc2 | 4.36 | 6.68E-15 | 2.45 | 5.15E-06 | 2.67 | 4.52E-07 |
| ENSMUSG00000020023 | Tmcc3 | 4.35 | 6.28E-19 | 4.32 | 1.62E-19 | 2.16 | 1.22E-05 |
| ENSMUSG00000045211 | Nudt18 | 4.34 | 4.19E-16 | 2.85 | 1.87E-08 | 2.75 | 7.59E-08 |
| ENSMUSG00000005360 | Slc1a3 | 4.33 | 1.80E-21 | 6.41 | 2.41E-34 | 6.9 | 5.78E-37 |
| ENSMUSG00000057069 | Ero1lb | 4.32 | 5.61E-07 | 8.35 | 7.42E-14 | 8.43 | 6.37E-14 |
| ENSMUSG00000039632 | Ccdc151 | 4.31 | 8.35E-14 | 2.62 | 5.78E-06 | 2.16 | 0.000488124 |
| ENSMUSG00000074577 | Fam65c | 4.31 | 0.001237319 | 12.12 | 1.73E-11 | 11.02 | 2.53E-10 |
| ENSMUSG00000021662 | Arhgef28 | 4.29 | 3.13E-22 | 5.41 | 4.69E-29 | 3 | 3.01E-13 |
| ENSMUSG00000022759 | 4930451C15Rik | 4.28 | 6.99E-06 | 3.82 | 3.86E-05 | 2.62 | 0.00872735 |
| ENSMUSG00000031442 | Mcf2l | 4.27 | 1.73E-19 | 3.91 | 1.21E-17 | 2.12 | 9.15E-06 |
| ENSMUSG00000041729 | Coro2b | 4.27 | 2.17E-20 | 2.98 | 3.15E-12 | 3.24 | 6.97E-14 |
| ENSMUSG00000020961 | Ston2 | 4.26 | 1.15E-21 | 8 | 8.12E-42 | 2.01 | 1.20E-05 |
| ENSMUSG00000037490 | Slc2a12 | 4.26 | 2.10E-10 | 3.11 | 1.09E-06 | 3.05 | 2.24E-06 |
| ENSMUSG00000026564 | Dusp27 | 4.23 | 3.25E-07 | 13.71 | 7.99E-25 | 6.28 | 3.31E-11 |
| ENSMUSG00000001313 | Rnd2 | 4.22 | 4.03E-22 | 5.46 | 4.56E-30 | 2.02 | 8.25E-06 |
| ENSMUSG00000023909 | Paqr4 | 4.22 | 1.39E-16 | 5.5 | 1.37E-23 | 3.62 | 1.22E-13 |
| ENSMUSG00000032172 | Olfm2 | 4.22 | 0.000708785 | 9.4 | 1.65E-07 | 4.6 | 0.000554128 |
| ENSMUSG00000029093 | Sorcs2 | 4.21 | 2.50E-10 | 20.35 | 7.91E-36 | 11.3 | 5.98E-25 |
| ENSMUSG00000051439 | Cd14 | 4.19 | 0.000572282 | 10.73 | 3.99E-10 | 2.98 | 0.018868592 |
| ENSMUSG00000067786 | Nnat | 4.16 | 0.002160181 | 3.06 | 0.022615626 | 4.92 | 0.000892975 |
| ENSMUSG00000052942 | Glis3 | 4.15 | 1.32E-06 | 11.9 | 5.72E-18 | 2.23 | 0.012566502 |
| ENSMUSG00000054702 | Ap1s3 | 4.15 | 1.01E-06 | 2.84 | 0.000175042 | 2.46 | 0.00302824 |
| ENSMUSG00000022048 | Dpysl2 | 4.14 | 2.78E-12 | 2.11 | 0.000180349 | 3.34 | 1.53E-09 |
| ENSMUSG00000035189 | Ano4 | 4.13 | 0.025767727 | 5.24 | 0.008579219 | 15.72 | 2.61E-08 |
| ENSMUSG00000037846 | Rtkn2 | 4.13 | 1.43E-20 | 2.86 | 4.41E-12 | 2.21 | 4.61E-07 |
| ENSMUSG00000033805 | Ephx4 | 4.12 | 0.000114226 | 4.83 | 7.48E-06 | 2.53 | 0.047181212 |
| ENSMUSG00000070570 | Slc17a7 | 4.12 | 0.000816713 | 7.31 | 1.06E-06 | 2.9 | 0.042038369 |
| ENSMUSG00000048281 | Dleu7 | 4.11 | 0.000653358 | 11.25 | 2.21E-10 | 7.59 | 1.68E-07 |
| ENSMUSG00000054459 | Vsnl1 | 4.11 | 6.29E-06 | 3.64 | 3.09E-05 | 2.77 | 0.002990877 |
| ENSMUSG00000022178 | Ajuba | 4.08 | 2.66E-22 | 4.4 | 8.35E-25 | 2.08 | 9.40E-07 |
| ENSMUSG00000051041 | Olfml1 | 4.08 | 0.04268081 | 6.97 | 0.003427238 | 12.49 | 6.66E-05 |
| ENSMUSG00000025780 | Itih5 | 4.06 | 7.98E-08 | 6.17 | 9.54E-13 | 6.52 | 2.39E-13 |
| ENSMUSG00000032380 | Dapk2 | 4.06 | 2.02E-05 | 4.15 | 1.28E-05 | 5.63 | 1.10E-07 |
| ENSMUSG00000039323 | Igfbp2 | 4.06 | 0.000123903 | 13.8 | 1.41E-12 | 4.24 | 0.000116432 |
| ENSMUSG00000020908 | Myh3 | 4.04 | 0.038529038 | 16.08 | 2.36E-05 | 4.37 | 0.037173773 |
| ENSMUSG00000031373 | Car5b | 4.04 | 5.96E-10 | 2.79 | 1.18E-05 | 3.56 | 2.65E-08 |
| ENSMUSG00000021986 | Amer2 | 4.02 | 9.53E-08 | 2.96 | 3.91E-05 | 2.13 | 0.008555447 |
| ENSMUSG00000024529 | Lox | 4.02 | 2.40E-05 | 5.54 | 4.38E-08 | 3.27 | 0.000342779 |
| ENSMUSG00000000794 | Kcnn3 | 4 | 1.79E-09 | 2.94 | 5.17E-06 | 2.91 | 6.42E-06 |
| ENSMUSG00000033192 | Lpcat2 | 3.99 | 1.20E-10 | 2.62 | 8.59E-06 | 2.12 | 0.001763823 |
| ENSMUSG00000020707 | Rnf135 | 3.98 | 9.88E-07 | 5.56 | 3.66E-10 | 3.94 | 1.29E-06 |
| ENSMUSG00000022987 | Zfp641 | 3.97 | 3.23E-12 | 4.72 | 1.38E-15 | 3.02 | 8.76E-08 |
| ENSMUSG00000049588 | Ccdc69 | 3.97 | 0.011813333 | 6.94 | 1.33E-05 | 5.95 | 0.000195902 |
| ENSMUSG00000041957 | Pkp2 | 3.96 | 4.82E-19 | 5.7 | 2.21E-30 | 2.53 | 2.30E-09 |
| ENSMUSG00000035840 | Lysmd3 | 3.95 | 3.29E-18 | 2.78 | 1.82E-11 | 2.25 | 2.78E-07 |
| ENSMUSG00000022769 | Sdf2l1 | 3.94 | 1.03E-18 | 2.04 | 9.14E-06 | 2.33 | 1.19E-07 |
| ENSMUSG00000006575 | Rundc3a | 3.92 | 3.15E-11 | 2.91 | 2.24E-07 | 2.12 | 0.000656649 |
| ENSMUSG00000030110 | Ret | 3.92 | 0.02314893 | 5.29 | 0.006649198 | 34.28 | 6.00E-09 |
| ENSMUSG00000040653 | Ppp1r14c | 3.9 | 1.23E-10 | 3.43 | 8.33E-10 | 2.39 | 8.84E-05 |
| ENSMUSG00000025854 | Fam20c | 3.89 | 2.16E-09 | 9.53 | 2.02E-23 | 3 | 3.15E-06 |
| ENSMUSG00000062444 | Ap3b2 | 3.89 | 6.36E-09 | 6.81 | 7.65E-18 | 4.26 | 1.47E-10 |
| ENSMUSG00000006586 | Runx1t1 | 3.88 | 0.001654676 | 7.17 | 1.20E-08 | 15.56 | 8.55E-17 |
| ENSMUSG00000027223 | Mapk8ip1 | 3.86 | 2.05E-20 | 4.59 | 1.26E-25 | 2.38 | 3.81E-09 |
| ENSMUSG00000030401 | Rtn2 | 3.84 | 5.11E-18 | 2.52 | 2.71E-09 | 2.12 | 2.53E-06 |
| ENSMUSG00000030606 | Hapln3 | 3.84 | 3.85E-09 | 5.47 | 2.83E-14 | 19.81 | 3.75E-42 |
| ENSMUSG00000095789 | Nupr1l | 3.84 | 0.000820166 | 2.81 | 0.019444098 | 2.54 | 0.047160704 |
| ENSMUSG00000097055 | AC140457.1 | 3.83 | 1.38E-08 | 3.24 | 4.76E-07 | 3.17 | 1.14E-06 |
| ENSMUSG00000039954 | Stk32a | 3.82 | 0.03949067 | 14.8 | 2.31E-08 | 4.54 | 0.006297017 |
| ENSMUSG00000040624 | Plekhg1 | 3.82 | 6.10E-11 | 3.5 | 7.15E-10 | 2.56 | 9.28E-06 |
| ENSMUSG00000073795 | 6430531B16Rik | 3.82 | 0.001184217 | 3.74 | 0.002894018 | 3.85 | 0.002667227 |
| ENSMUSG00000078653 | Cntd1 | 3.81 | 6.13E-05 | 4.57 | 8.40E-06 | 4.18 | 4.68E-05 |
| ENSMUSG00000041842 | Fhdc1 | 3.8 | 7.10E-05 | 18.97 | 1.77E-18 | 5.84 | 1.17E-07 |
| ENSMUSG00000041889 | Shisa4 | 3.8 | 2.54E-17 | 2.59 | 3.24E-09 | 2.62 | 2.58E-09 |
| ENSMUSG00000048600 | Gm5763 | 3.8 | 5.01E-12 | 3.84 | 5.09E-12 | 6.42 | 1.93E-22 |
| ENSMUSG00000042428 | Mgat3 | 3.73 | 1.48E-18 | 4.16 | 2.00E-21 | 4.8 | 5.99E-26 |
| ENSMUSG00000035773 | Kiss1r | 3.72 | 0.031805346 | 8.86 | 3.41E-05 | 5.43 | 0.003497794 |
| ENSMUSG00000018593 | Sparc | 3.71 | 1.34E-19 | 4.16 | 2.54E-23 | 2.58 | 1.45E-10 |
| ENSMUSG00000027452 | Acss1 | 3.71 | 4.19E-06 | 4.85 | 9.64E-09 | 2.35 | 0.005926615 |
| ENSMUSG00000032265 | Fam46a | 3.71 | 3.58E-05 | 3.76 | 1.73E-05 | 3.08 | 0.000539199 |
| ENSMUSG00000059810 | Rgs3 | 3.71 | 2.19E-15 | 5.15 | 1.42E-22 | 5.2 | 1.40E-22 |
| ENSMUSG00000070802 | Pnmal2 | 3.71 | 1.21E-14 | 3.16 | 1.75E-11 | 3.28 | 2.53E-12 |
| ENSMUSG00000075318 | Scn2a1 | 3.71 | 0.00011116 | 2.62 | 0.002955969 | 2.48 | 0.009147823 |
| ENSMUSG00000038453 | Srcin1 | 3.68 | 5.85E-17 | 4.4 | 6.53E-22 | 2.46 | 1.12E-08 |
| ENSMUSG00000097561 | AC171500.1 | 3.68 | 0.046527136 | 4.49 | 0.011375609 | 4.12 | 0.020300715 |
| ENSMUSG00000036718 | Micall2 | 3.66 | 8.20E-16 | 3.32 | 1.25E-12 | 22.78 | 7.64E-76 |
| ENSMUSG00000017009 | Sdc4 | 3.65 | 0.000244328 | 4.03 | 6.40E-05 | 2.92 | 0.003679806 |
| ENSMUSG00000029287 | Tgfbr3 | 3.63 | 1.32E-13 | 3.84 | 3.65E-15 | 5.19 | 3.95E-22 |
| ENSMUSG00000048616 | Nog | 3.61 | 1.10E-05 | 4.36 | 8.92E-08 | 3.05 | 0.00011223 |
| ENSMUSG00000055202 | Zfp811 | 3.6 | 1.82E-16 | 5.03 | 1.80E-25 | 2.43 | 2.04E-08 |
| ENSMUSG00000070106 | Mir363 | 3.58 | 0.001745428 | 3.44 | 0.003928521 | 6.17 | 8.45E-07 |
| ENSMUSG00000028005 | Gucy1b3 | 3.57 | 7.53E-06 | 3.05 | 0.000110034 | 2.67 | 0.001261991 |
| ENSMUSG00000045103 | Dmd | 3.57 | 2.03E-07 | 8.57 | 1.36E-19 | 5.66 | 2.57E-13 |
| ENSMUSG00000024803 | Ankrd1 | 3.56 | 0.000426519 | 4.23 | 5.11E-05 | 2.43 | 0.032411991 |
| ENSMUSG00000034413 | Neurl1b | 3.55 | 2.02E-06 | 5.22 | 2.66E-10 | 4.58 | 1.49E-08 |
| ENSMUSG00000048481 | Mypop | 3.54 | 9.84E-09 | 2.64 | 2.60E-05 | 2.2 | 0.001406006 |
| ENSMUSG00000057751 | Megf6 | 3.52 | 3.14E-16 | 3.61 | 1.79E-16 | 2.44 | 1.67E-08 |
| ENSMUSG00000045102 | Poln | 3.51 | 0.00061177 | 4.08 | 3.09E-05 | 2.61 | 0.013196398 |
| ENSMUSG00000048915 | Efna5 | 3.51 | 6.73E-14 | 3.49 | 2.73E-14 | 2.2 | 5.21E-06 |
| ENSMUSG00000032515 | Csrnp1 | 3.5 | 2.98E-14 | 4.23 | 1.63E-19 | 2.6 | 8.09E-09 |
| ENSMUSG00000056313 | 1810011O10Rik | 3.5 | 4.98E-10 | 5.38 | 1.22E-19 | 3.55 | 5.95E-11 |
| ENSMUSG00000001665 | Gstt3 | 3.48 | 0.000784536 | 3.88 | 0.000293145 | 3.68 | 0.000586723 |
| ENSMUSG00000021186 | Fbln5 | 3.48 | 2.82E-05 | 6.14 | 5.82E-12 | 2.78 | 0.000849598 |
| ENSMUSG00000032419 | Tbx18 | 3.48 | 0.009985903 | 3.51 | 0.009909892 | 55.68 | 2.05E-19 |
| ENSMUSG00000093930 | Hmgcs1 | 3.48 | 3.66E-17 | 2.87 | 2.90E-13 | 2.99 | 4.10E-14 |
| ENSMUSG00000020182 | Ddc | 3.47 | 1.21E-10 | 3.25 | 7.11E-10 | 3.29 | 9.23E-10 |
| ENSMUSG00000028072 | Ntrk1 | 3.46 | 0.019116893 | 9.11 | 6.77E-06 | 8.83 | 1.04E-05 |
| ENSMUSG00000054885 | 4930578G10Rik | 3.44 | 0.013226565 | 5.05 | 0.000723187 | 3.46 | 0.024643344 |
| ENSMUSG00000026463 | Atp2b4 | 3.42 | 1.04E-09 | 7.06 | 1.55E-24 | 2.33 | 3.27E-05 |
| ENSMUSG00000027221 | Chst1 | 3.41 | 1.67E-05 | 3.64 | 8.49E-06 | 3.83 | 3.66E-06 |
| ENSMUSG00000039270 | Megf9 | 3.41 | 0.000685648 | 2.42 | 0.019509712 | 2.41 | 0.023296847 |
| ENSMUSG00000039934 | Gsap | 3.41 | 4.85E-07 | 7.43 | 2.35E-18 | 2.93 | 1.76E-05 |
| ENSMUSG00000036446 | Lum | 3.4 | 0.027192906 | 10.91 | 2.18E-05 | 41.37 | 1.17E-10 |
| ENSMUSG00000001566 | Mnx1 | 3.38 | 6.23E-05 | 3.05 | 0.000283451 | 3.45 | 3.83E-05 |
| ENSMUSG00000030342 | Cd9 | 3.38 | 4.45E-11 | 3.27 | 2.54E-11 | 2.2 | 2.68E-05 |
| ENSMUSG00000040852 | Plekhh2 | 3.38 | 2.90E-16 | 2.35 | 1.25E-08 | 2.3 | 3.07E-08 |
| ENSMUSG00000048096 | Lmod1 | 3.37 | 0.000106388 | 3.52 | 5.20E-05 | 2.71 | 0.002955994 |
| ENSMUSG00000042312 | S100a13 | 3.36 | 1.06E-08 | 4.32 | 5.17E-12 | 4.93 | 9.91E-14 |
| ENSMUSG00000058740 | Kcnt1 | 3.36 | 0.003579223 | 9.15 | 5.38E-11 | 2.92 | 0.02414032 |
| ENSMUSG00000092193 | Cd9-ps | 3.34 | 0.022880309 | 5.06 | 0.000490872 | 3.4 | 0.02421701 |
| ENSMUSG00000001053 | N4bp3 | 3.33 | 8.83E-16 | 3.68 | 3.74E-18 | 2.33 | 4.32E-08 |
| ENSMUSG00000020583 | Matn3 | 3.33 | 6.71E-07 | 3.67 | 3.64E-08 | 2.9 | 1.49E-05 |
| ENSMUSG00000075224 | Lrrc55 | 3.32 | 0.015480244 | 3.28 | 0.008142591 | 10.92 | 6.02E-12 |
| ENSMUSG00000054871 | Tmem158 | 3.31 | 9.35E-11 | 2.47 | 6.28E-07 | 2.41 | 2.76E-06 |
| ENSMUSG00000031736 | 4933436C20Rik | 3.29 | 0.001028158 | 6.15 | 1.57E-07 | 2.45 | 0.024607205 |
| ENSMUSG00000047181 | Samd14 | 3.29 | 1.06E-10 | 2.84 | 2.33E-08 | 2.52 | 8.06E-07 |
| ENSMUSG00000035578 | Iqcg | 3.27 | 8.45E-12 | 2.46 | 2.66E-07 | 2.25 | 9.92E-06 |
| ENSMUSG00000046157 | Tmem229b | 3.27 | 3.81E-06 | 3.19 | 1.12E-05 | 2.4 | 0.001668028 |
| ENSMUSG00000027506 | Tpd52 | 3.26 | 8.79E-06 | 2.95 | 3.95E-05 | 3.44 | 2.95E-06 |
| ENSMUSG00000042734 | Ttc9 | 3.26 | 5.79E-10 | 5.03 | 5.70E-18 | 2.2 | 0.000198989 |
| ENSMUSG00000062995 | Ica1 | 3.26 | 1.73E-13 | 3.35 | 1.55E-14 | 3.85 | 3.27E-17 |
| ENSMUSG00000052301 | Doc2a | 3.25 | 1.11E-07 | 16.3 | 2.94E-39 | 2.63 | 3.22E-05 |
| ENSMUSG00000005803 | Sqrdl | 3.24 | 4.90E-06 | 3.63 | 8.24E-07 | 2.82 | 0.000367536 |
| ENSMUSG00000038060 | Dlec1 | 3.24 | 1.82E-05 | 7.5 | 1.50E-15 | 3.94 | 6.11E-07 |
| ENSMUSG00000040990 | Sh3kbp1 | 3.24 | 2.34E-10 | 3.95 | 4.06E-15 | 2.87 | 3.83E-09 |
| ENSMUSG00000042918 | Mamstr | 3.24 | 7.53E-06 | 2.44 | 0.001410481 | 2.69 | 0.000225281 |
| ENSMUSG00000058070 | Eml1 | 3.24 | 1.49E-09 | 3.5 | 1.38E-10 | 3.91 | 4.20E-12 |
| ENSMUSG00000020814 | Mxra7 | 3.23 | 1.01E-15 | 5.36 | 7.00E-30 | 2.92 | 8.79E-13 |
| ENSMUSG00000052676 | Zmat1 | 3.23 | 2.93E-09 | 2.57 | 8.01E-07 | 2.93 | 1.58E-08 |
| ENSMUSG00000018411 | Mapt | 3.22 | 0.005965428 | 4.26 | 0.000587251 | 8.4 | 2.20E-07 |
| ENSMUSG00000022037 | Clu | 3.21 | 0.002722572 | 3.79 | 0.000730643 | 8.1 | 8.71E-08 |
| ENSMUSG00000026970 | Rbms1 | 3.21 | 2.59E-14 | 2.21 | 4.27E-08 | 2.76 | 3.11E-12 |
| ENSMUSG00000040183 | Ankrd6 | 3.21 | 5.93E-15 | 4.34 | 3.74E-23 | 3.67 | 3.51E-18 |
| ENSMUSG00000082791 | Gm4875 | 3.2 | 2.13E-12 | 2.59 | 2.02E-08 | 2.09 | 2.37E-05 |
| ENSMUSG00000027805 | Pfn2 | 3.19 | 1.34E-14 | 2.62 | 5.06E-11 | 2.08 | 1.73E-06 |
| ENSMUSG00000021384 | Susd3 | 3.18 | 0.010379859 | 8.21 | 8.25E-08 | 5.8 | 2.02E-05 |
| ENSMUSG00000097093 | AC123724.1 | 3.18 | 0.005849642 | 2.49 | 0.040193362 | 3.41 | 0.001932573 |
| ENSMUSG00000050821 | Fam131a | 3.17 | 2.41E-12 | 3.1 | 1.21E-11 | 2.49 | 1.07E-07 |
| ENSMUSG00000052525 | Spdya | 3.17 | 0.001347993 | 2.16 | 0.033979816 | 2.77 | 0.00575086 |
| ENSMUSG00000040998 | Npnt | 3.16 | 2.82E-13 | 4.33 | 1.03E-20 | 2.15 | 3.66E-06 |
| ENSMUSG00000069806 | Cacng7 | 3.16 | 2.34E-15 | 2.05 | 1.00E-06 | 2.06 | 8.25E-07 |
| ENSMUSG00000057969 | Sema3b | 3.15 | 2.37E-11 | 6.93 | 3.01E-29 | 3.06 | 4.35E-10 |
| ENSMUSG00000067276 | Capn6 | 3.15 | 2.08E-07 | 2.69 | 1.09E-05 | 2.29 | 0.000318927 |
| ENSMUSG00000067818 | Myl9 | 3.15 | 2.58E-07 | 3.52 | 1.83E-08 | 3.31 | 1.40E-07 |
| ENSMUSG00000004665 | Cnn2 | 3.14 | 1.05E-14 | 3.76 | 1.29E-19 | 3.94 | 8.35E-21 |
| ENSMUSG00000028645 | Slc2a1 | 3.14 | 1.29E-13 | 3.81 | 3.21E-18 | 3.34 | 5.35E-15 |
| ENSMUSG00000011884 | Gltp | 3.13 | 2.49E-14 | 2.38 | 6.58E-09 | 2.09 | 2.02E-06 |
| ENSMUSG00000037138 | Aff3 | 3.13 | 5.11E-11 | 7.08 | 1.38E-30 | 3.6 | 4.67E-14 |
| ENSMUSG00000028583 | Pdpn | 3.12 | 1.28E-09 | 2.71 | 1.06E-07 | 2.04 | 0.000324537 |
| ENSMUSG00000040093 | Bmf | 3.12 | 4.95E-15 | 4.81 | 6.82E-27 | 5.2 | 2.73E-29 |
| ENSMUSG00000033006 | Sox10 | 3.11 | 0.011793521 | 3.52 | 0.007202853 | 6.65 | 1.18E-05 |
| ENSMUSG00000033313 | Fbxl8 | 3.11 | 2.00E-05 | 5.9 | 9.39E-13 | 4.19 | 3.66E-08 |
| ENSMUSG00000011589 | Fsd1 | 3.1 | 5.12E-14 | 3.61 | 2.88E-17 | 2.4 | 1.59E-08 |
| ENSMUSG00000031887 | Tradd | 3.1 | 2.06E-11 | 3.87 | 4.61E-16 | 3.14 | 1.70E-11 |
| ENSMUSG00000040711 | Sh3pxd2b | 3.09 | 1.85E-08 | 2.58 | 3.23E-06 | 2.02 | 0.000823946 |
| ENSMUSG00000022012 | Enox1 | 3.08 | 1.48E-10 | 2.04 | 0.000115105 | 2.57 | 1.16E-07 |
| ENSMUSG00000046814 | Gchfr | 3.08 | 1.21E-08 | 2.19 | 0.000147061 | 2.77 | 3.94E-07 |
| ENSMUSG00000038605 | Samd10 | 3.07 | 3.42E-12 | 3.45 | 6.69E-15 | 2.2 | 2.77E-06 |
| ENSMUSG00000037446 | Tulp1 | 3.06 | 3.71E-07 | 3.79 | 1.69E-09 | 3.06 | 1.27E-06 |
| ENSMUSG00000049521 | Cdc42ep1 | 3.06 | 1.39E-12 | 4.79 | 1.13E-22 | 6.39 | 3.87E-31 |
| ENSMUSG00000024206 | Rfx2 | 3.05 | 7.50E-10 | 7.68 | 1.52E-31 | 3.35 | 1.08E-11 |
| ENSMUSG00000041120 | Nbl1 | 3.05 | 4.73E-08 | 2.42 | 2.64E-05 | 13.01 | 6.10E-43 |
| ENSMUSG00000020684 | Rasl10b | 3.04 | 2.96E-13 | 4.07 | 1.24E-20 | 3.48 | 1.48E-16 |
| ENSMUSG00000006360 | Crip1 | 3.03 | 3.76E-05 | 6.23 | 6.88E-13 | 3.33 | 9.91E-06 |
| ENSMUSG00000034706 | Dnaic2 | 3.02 | 5.61E-05 | 7.5 | 1.88E-17 | 10.06 | 8.82E-23 |
| ENSMUSG00000039058 | Ak5 | 3.02 | 1.83E-06 | 2.91 | 1.19E-06 | 2.85 | 4.73E-06 |
| ENSMUSG00000036904 | Fzd8 | 3 | 2.06E-11 | 4.04 | 3.45E-18 | 4.22 | 2.87E-19 |
| ENSMUSG00000096299 | Gm21814 | 3 | 0.003519267 | 2.35 | 0.025786428 | 2.8 | 0.005737694 |
| ENSMUSG00000001227 | Sema6b | 2.99 | 6.96E-06 | 3.74 | 4.87E-08 | 3.87 | 2.89E-08 |
| ENSMUSG00000006362 | Cbfa2t3 | 2.99 | 7.95E-07 | 4.72 | 4.46E-14 | 6.57 | 1.92E-20 |
| ENSMUSG00000042228 | Lyn | 2.99 | 1.07E-10 | 2.46 | 1.51E-07 | 4.92 | 8.84E-22 |
| ENSMUSG00000022055 | Nefl | 2.98 | 1.48E-11 | 2.05 | 7.25E-06 | 2.77 | 1.07E-10 |
| ENSMUSG00000028399 | Ptprd | 2.98 | 0.00188012 | 4.55 | 5.78E-06 | 2.97 | 0.001887284 |
| ENSMUSG00000039347 | Atp6v0e2 | 2.98 | 2.24E-13 | 3.45 | 1.55E-16 | 2.3 | 8.69E-08 |
| ENSMUSG00000055799 | Tcf7l1 | 2.98 | 1.17E-13 | 2.8 | 1.16E-12 | 2.54 | 2.03E-10 |
| ENSMUSG00000020155 | Kcnmb1 | 2.97 | 0.013240207 | 2.66 | 0.038098806 | 8 | 1.96E-09 |
| ENSMUSG00000022483 | Col2a1 | 2.97 | 0.000205968 | 2.11 | 0.018157605 | 2.76 | 0.000847963 |
| ENSMUSG00000022574 | Naprt1 | 2.97 | 2.05E-08 | 4.21 | 1.15E-13 | 2.94 | 6.83E-08 |
| ENSMUSG00000035200 | Chrnb4 | 2.97 | 2.33E-05 | 4.82 | 7.72E-10 | 2.62 | 0.000321324 |
| ENSMUSG00000027306 | Nusap1 | 2.96 | 2.08E-13 | 2.27 | 3.08E-08 | 2.13 | 5.57E-07 |
| ENSMUSG00000034110 | Kctd7 | 2.96 | 9.59E-09 | 2.17 | 5.44E-05 | 2.42 | 3.09E-06 |
| ENSMUSG00000037627 | Rgs22 | 2.95 | 0.000951868 | 5.59 | 3.51E-09 | 3.81 | 2.53E-05 |
| ENSMUSG00000045534 | Kcna5 | 2.95 | 0.000394869 | 2.64 | 0.001335222 | 4.51 | 1.93E-07 |
| ENSMUSG00000022150 | Dab2 | 2.94 | 4.63E-05 | 6.68 | 9.10E-14 | 2.06 | 0.011982368 |
| ENSMUSG00000017943 | Gdap1l1 | 2.93 | 4.44E-10 | 2.68 | 8.30E-09 | 2.04 | 0.000106994 |
| ENSMUSG00000022773 | Ypel1 | 2.93 | 2.32E-12 | 2.95 | 7.39E-13 | 2.56 | 6.55E-10 |
| ENSMUSG00000006731 | B4galnt1 | 2.91 | 8.71E-12 | 4.27 | 1.89E-20 | 3.11 | 1.01E-12 |
| ENSMUSG00000025885 | Myo5b | 2.91 | 0.022042659 | 3.12 | 0.01728734 | 3.52 | 0.008836462 |
| ENSMUSG00000044122 | Proca1 | 2.91 | 2.66E-07 | 3.38 | 3.51E-10 | 5.02 | 3.78E-17 |
| ENSMUSG00000001435 | Col18a1 | 2.89 | 5.27E-14 | 5.86 | 5.53E-34 | 2.68 | 1.55E-11 |
| ENSMUSG00000023341 | Mx2 | 2.89 | 0.010822861 | 3.95 | 0.000585679 | 3.33 | 0.005213132 |
| ENSMUSG00000040841 | Six5 | 2.89 | 4.20E-12 | 3.51 | 1.72E-16 | 3.11 | 1.10E-13 |
| ENSMUSG00000041703 | Zic5 | 2.89 | 0.010574904 | 3.56 | 0.000766253 | 3.61 | 0.000938046 |
| ENSMUSG00000070000 | Fcho1 | 2.89 | 3.55E-09 | 4.82 | 1.70E-18 | 2.14 | 9.23E-05 |
| ENSMUSG00000028681 | Ptch2 | 2.88 | 1.72E-08 | 4.74 | 1.92E-17 | 5 | 1.03E-18 |
| ENSMUSG00000020782 | Llgl2 | 2.87 | 4.65E-05 | 7.1 | 3.18E-14 | 3.87 | 2.02E-07 |
| ENSMUSG00000029185 | Fam114a1 | 2.86 | 7.27E-12 | 3.41 | 1.18E-15 | 4.97 | 1.04E-25 |
| ENSMUSG00000040502 | 1/3/09 | 2.86 | 4.13E-12 | 2.69 | 1.37E-10 | 2.01 | 1.62E-05 |
| ENSMUSG00000041362 | 4930506M07Rik | 2.86 | 5.90E-06 | 9.29 | 8.54E-30 | 2.75 | 2.37E-06 |
| ENSMUSG00000022432 | Smc1b | 2.85 | 0.00225506 | 4.54 | 2.58E-07 | 3.16 | 0.000323222 |
| ENSMUSG00000037166 | Ppp1r14a | 2.84 | 0.001285007 | 3.92 | 1.29E-05 | 7.42 | 5.10E-11 |
| ENSMUSG00000052331 | Ankrd44 | 2.84 | 4.44E-09 | 3.44 | 5.71E-13 | 2.77 | 6.30E-09 |
| ENSMUSG00000089832 | Shkbp1 | 2.84 | 3.38E-12 | 2.83 | 4.93E-12 | 2.32 | 4.53E-08 |
| ENSMUSG00000036923 | Stox1 | 2.83 | 0.003279773 | 4.07 | 2.51E-05 | 2.26 | 0.031379578 |
| ENSMUSG00000024663 | Rab3il1 | 2.82 | 2.01E-09 | 3.12 | 2.19E-11 | 19.9 | 7.67E-68 |
| ENSMUSG00000028876 | Epha10 | 2.82 | 0.004630054 | 7.02 | 1.69E-08 | 2.66 | 0.017222128 |
| ENSMUSG00000034382 | AI661453 | 2.82 | 0.006275152 | 6.6 | 6.15E-08 | 4.38 | 3.11E-05 |
| ENSMUSG00000035104 | Eva1a | 2.82 | 0.035795375 | 10.05 | 1.15E-10 | 7.93 | 2.22E-08 |
| ENSMUSG00000078566 | Bnip3 | 2.82 | 0.010146743 | 2.92 | 0.005793202 | 2.33 | 0.043840713 |
| ENSMUSG00000027111 | Itga6 | 2.81 | 5.42E-12 | 3.26 | 3.05E-16 | 3.01 | 4.67E-14 |
| ENSMUSG00000029864 | Gstk1 | 2.81 | 0.000347981 | 4.86 | 4.27E-09 | 5.94 | 2.88E-11 |
| ENSMUSG00000061740 | Cyp2d22 | 2.81 | 0.000135959 | 4.54 | 8.20E-09 | 5.85 | 7.75E-12 |
| ENSMUSG00000027695 | Pld1 | 2.8 | 4.31E-08 | 3.48 | 1.32E-11 | 2.39 | 9.96E-06 |
| ENSMUSG00000031872 | Bean1 | 2.8 | 0.014532389 | 5.51 | 8.14E-06 | 6.39 | 1.48E-06 |
| ENSMUSG00000022040 | Ephx2 | 2.79 | 0.000141769 | 4.08 | 8.27E-08 | 2.11 | 0.011473987 |
| ENSMUSG00000037628 | Cdkn3 | 2.79 | 2.29E-10 | 2.53 | 1.63E-08 | 2.87 | 1.66E-10 |
| ENSMUSG00000068105 | Tnfrsf13c | 2.79 | 0.004974366 | 2.4 | 0.033644108 | 2.67 | 0.020396198 |
| ENSMUSG00000022610 | Mapk12 | 2.77 | 4.31E-12 | 2.98 | 1.12E-13 | 2.23 | 1.51E-07 |
| ENSMUSG00000047507 | Baiap3 | 2.77 | 0.003380811 | 8.34 | 8.59E-12 | 2.58 | 0.011805348 |
| ENSMUSG00000026737 | Pip4k2a | 2.76 | 7.73E-11 | 2.76 | 2.87E-11 | 2.63 | 5.23E-10 |
| ENSMUSG00000037813 | D630003M21Rik | 2.76 | 0.010345204 | 4.62 | 5.12E-06 | 3.11 | 0.002989595 |
| ENSMUSG00000079055 | Slc8a3 | 2.76 | 0.00135829 | 2.83 | 0.001696062 | 11.43 | 9.10E-15 |
| ENSMUSG00000087371 | Gm15541 | 2.76 | 0.001873874 | 3.4 | 6.68E-05 | 3.52 | 5.14E-05 |
| ENSMUSG00000003863 | Ppfia3 | 2.75 | 2.61E-09 | 3.09 | 4.10E-11 | 2.02 | 0.000141079 |
| ENSMUSG00000022265 | Ank | 2.75 | 5.44E-11 | 4.7 | 2.54E-25 | 2.46 | 3.30E-09 |
| ENSMUSG00000089804 | Gm16136 | 2.74 | 0.004599411 | 2.96 | 0.001133234 | 2.49 | 0.015443325 |
| ENSMUSG00000024659 | Anxa1 | 2.73 | 0.00127948 | 3.41 | 2.55E-05 | 2.24 | 0.012442743 |
| ENSMUSG00000024747 | Aldh1a7 | 2.73 | 8.46E-10 | 4.47 | 7.53E-20 | 2.48 | 1.10E-07 |
| ENSMUSG00000038366 | Lasp1 | 2.73 | 1.57E-12 | 2.48 | 2.47E-10 | 2.5 | 2.33E-10 |
| ENSMUSG00000046546 | Fam43a | 2.73 | 1.38E-10 | 2.7 | 8.37E-11 | 3.14 | 1.02E-13 |
| ENSMUSG00000050947 | Amigo1 | 2.73 | 1.10E-09 | 3.75 | 1.29E-16 | 2.68 | 1.83E-09 |
| ENSMUSG00000029032 | Arhgef16 | 2.72 | 0.000834659 | 3.97 | 5.17E-06 | 2.59 | 0.003509016 |
| ENSMUSG00000050914 | Ankrd37 | 2.72 | 0.008655216 | 3.7 | 0.000131752 | 2.79 | 0.005364414 |
| ENSMUSG00000054146 | Krt15 | 2.72 | 0.001178195 | 4.25 | 4.94E-07 | 2.45 | 0.005383988 |
| ENSMUSG00000079235 | Ccdc13 | 2.72 | 0.002222187 | 3.9 | 5.01E-06 | 4.08 | 3.51E-06 |
| ENSMUSG00000034334 | Fam151b | 2.7 | 0.000238615 | 2.58 | 0.000343847 | 2.07 | 0.015499962 |
| ENSMUSG00000026655 | Fam107b | 2.68 | 1.35E-10 | 2.16 | 3.90E-07 | 2.37 | 9.15E-09 |
| ENSMUSG00000037621 | Atoh8 | 2.68 | 0.009158724 | 3.56 | 0.000723187 | 6.67 | 2.45E-07 |
| ENSMUSG00000020422 | Tns3 | 2.67 | 7.58E-10 | 4.79 | 3.76E-23 | 4 | 1.98E-18 |
| ENSMUSG00000040447 | Spns2 | 2.67 | 0.00538305 | 2.81 | 0.005762947 | 6.54 | 4.31E-08 |
| ENSMUSG00000028047 | Thbs3 | 2.66 | 3.82E-09 | 4.16 | 1.13E-18 | 2.75 | 1.01E-09 |
| ENSMUSG00000019320 | Noxo1 | 2.65 | 0.014659986 | 2.12 | 0.041564893 | 2.73 | 0.003935447 |
| ENSMUSG00000004891 | Nes | 2.63 | 3.19E-11 | 4.97 | 5.78E-29 | 2.88 | 2.27E-13 |
| ENSMUSG00000038807 | Rap1gap2 | 2.62 | 3.99E-09 | 5.11 | 2.11E-23 | 2.44 | 1.03E-07 |
| ENSMUSG00000031842 | Pde4c | 2.61 | 0.000249471 | 5.88 | 1.60E-13 | 2.99 | 1.21E-05 |
| ENSMUSG00000052125 | F730043M19Rik | 2.61 | 6.02E-05 | 3.77 | 2.73E-09 | 2.68 | 4.85E-05 |
| ENSMUSG00000038248 | Sobp | 2.6 | 2.06E-05 | 2.47 | 2.47E-05 | 2.41 | 5.58E-05 |
| ENSMUSG00000027078 | Ube2l6 | 2.59 | 2.06E-07 | 2.27 | 9.42E-06 | 3.97 | 9.56E-15 |
| ENSMUSG00000074218 | Cox7a1 | 2.59 | 0.000456254 | 4.49 | 1.05E-09 | 3.25 | 6.95E-06 |
| ENSMUSG00000039007 | Cpq | 2.58 | 4.70E-06 | 2.5 | 1.09E-05 | 2.13 | 0.000670862 |
| ENSMUSG00000045467 | Ttll13 | 2.57 | 0.010895627 | 3.02 | 0.000989624 | 3.6 | 0.000124968 |
| ENSMUSG00000036395 | Glb1l2 | 2.56 | 1.95E-08 | 2.88 | 1.90E-09 | 2.19 | 1.17E-05 |
| ENSMUSG00000039021 | Ttc16 | 2.56 | 0.003853925 | 3 | 0.000537169 | 2.12 | 0.047882533 |
| ENSMUSG00000040146 | Rgl3 | 2.56 | 0.012581171 | 3.61 | 0.000611029 | 3.03 | 0.004165011 |
| ENSMUSG00000002504 | Slc9a3r2 | 2.55 | 6.61E-09 | 2.33 | 3.50E-07 | 2.34 | 3.56E-07 |
| ENSMUSG00000050666 | Vstm4 | 2.55 | 0.002341595 | 6.82 | 5.28E-15 | 9.82 | 4.46E-21 |
| ENSMUSG00000063704 | Mapk15 | 2.55 | 0.000497502 | 4.82 | 4.49E-10 | 2.07 | 0.016136154 |
| ENSMUSG00000021294 | Kif26a | 2.53 | 2.69E-09 | 2.7 | 1.63E-10 | 4.65 | 7.60E-24 |
| ENSMUSG00000041617 | Ccdc74a | 2.53 | 0.002902757 | 3.45 | 2.05E-05 | 2.83 | 0.000899361 |
| ENSMUSG00000060519 | Tor3a | 2.53 | 2.65E-09 | 2.84 | 1.74E-11 | 2.64 | 9.28E-10 |
| ENSMUSG00000040978 | Gm11992 | 2.52 | 0.003254965 | 2.17 | 0.013226583 | 3.83 | 1.07E-06 |
| ENSMUSG00000089665 | Fcor | 2.52 | 4.76E-05 | 2.17 | 0.001099602 | 2.61 | 5.37E-05 |
| ENSMUSG00000096883 | AI848285 | 2.52 | 0.000107204 | 6.68 | 2.99E-18 | 2.89 | 9.97E-06 |
| ENSMUSG00000002980 | Bcam | 2.5 | 0.000992257 | 4.57 | 1.75E-08 | 3.54 | 5.50E-06 |
| ENSMUSG00000046637 | Ttc34 | 2.5 | 0.012208267 | 3.51 | 8.87E-05 | 2.17 | 0.04909246 |
| ENSMUSG00000025723 | Nmb | 2.49 | 2.44E-05 | 2.85 | 1.49E-06 | 2.88 | 1.39E-06 |
| ENSMUSG00000058135 | Gstm1 | 2.49 | 1.89E-09 | 2.39 | 2.18E-08 | 2.22 | 4.92E-07 |
| ENSMUSG00000021185 | 9030617O03Rik | 2.48 | 0.011306113 | 3.07 | 0.001828586 | 2.42 | 0.040442425 |
| ENSMUSG00000027860 | Vangl1 | 2.48 | 2.47E-09 | 4.81 | 4.12E-26 | 3.47 | 7.19E-17 |
| ENSMUSG00000037960 | 1110007C09Rik | 2.48 | 4.67E-06 | 2.66 | 2.58E-07 | 3.71 | 5.47E-12 |
| ENSMUSG00000025856 | Pdgfa | 2.47 | 1.29E-06 | 2.29 | 9.72E-06 | 2.02 | 0.00039226 |
| ENSMUSG00000032334 | Loxl1 | 2.47 | 3.11E-07 | 5.21 | 5.93E-23 | 4.09 | 1.40E-16 |
| ENSMUSG00000041696 | Rasl12 | 2.47 | 0.001580134 | 2.49 | 0.001145509 | 2.96 | 4.84E-05 |
| ENSMUSG00000043003 | Rasef | 2.47 | 0.019067577 | 3.93 | 1.84E-06 | 2.32 | 0.017273479 |
| ENSMUSG00000032285 | Dnaja4 | 2.46 | 4.73E-06 | 2.65 | 3.83E-07 | 2.4 | 1.19E-05 |
| ENSMUSG00000029070 | Mxra8 | 2.44 | 3.84E-09 | 2.73 | 5.05E-11 | 2.06 | 7.22E-06 |
| ENSMUSG00000026471 | Mr1 | 2.43 | 0.010737294 | 3.07 | 0.000204673 | 4.16 | 1.59E-06 |
| ENSMUSG00000041809 | Efhc1 | 2.43 | 2.72E-06 | 3.44 | 7.37E-12 | 3.69 | 8.25E-13 |
| ENSMUSG00000079662 | Ntn3 | 2.43 | 3.18E-06 | 3.8 | 8.08E-13 | 2.9 | 4.41E-08 |
| ENSMUSG00000075702 | Selm | 2.42 | 3.45E-09 | 2.35 | 2.29E-08 | 2.87 | 3.55E-12 |
| ENSMUSG00000091561 | Gm6665 | 2.42 | 1.04E-06 | 2.65 | 1.46E-08 | 2.01 | 0.000209747 |
| ENSMUSG00000030657 | Xylt1 | 2.41 | 0.007409997 | 4.3 | 5.08E-06 | 2.1 | 0.042036152 |
| ENSMUSG00000031227 | Magee1 | 2.41 | 2.97E-08 | 2.19 | 1.47E-06 | 2.44 | 1.95E-08 |
| ENSMUSG00000033066 | Gas7 | 2.41 | 0.000177313 | 3.32 | 1.37E-07 | 2.72 | 1.91E-05 |
| ENSMUSG00000043811 | Rtn4r | 2.41 | 0.000176152 | 3.4 | 2.93E-08 | 2.57 | 6.47E-05 |
| ENSMUSG00000056427 | Slit3 | 2.41 | 9.53E-08 | 2.7 | 1.85E-09 | 2.15 | 6.84E-06 |
| ENSMUSG00000041012 | Cmtm8 | 2.4 | 8.32E-06 | 5.14 | 1.37E-18 | 4.09 | 4.25E-14 |
| ENSMUSG00000037977 | 6430571L13Rik | 2.39 | 0.007980074 | 2.36 | 0.018242582 | 2.63 | 0.005840254 |
| ENSMUSG00000040562 | Gstm2 | 2.39 | 1.46E-06 | 2.7 | 9.89E-09 | 2.37 | 1.61E-06 |
| ENSMUSG00000017774 | Myo1c | 2.38 | 4.75E-09 | 2.67 | 2.06E-11 | 2.04 | 3.10E-06 |
| ENSMUSG00000038742 | Angptl6 | 2.38 | 4.48E-05 | 2.73 | 1.40E-06 | 2.7 | 3.44E-06 |
| ENSMUSG00000047261 | Gap43 | 2.38 | 6.13E-07 | 2.46 | 2.24E-08 | 2.11 | 1.10E-05 |
| ENSMUSG00000094594 | Tpd52-ps | 2.38 | 0.000432016 | 2.72 | 5.64E-05 | 2.66 | 0.000105476 |
| ENSMUSG00000022015 | Tnfsf11 | 2.37 | 0.01902134 | 12.39 | 7.79E-26 | 4.47 | 1.88E-07 |
| ENSMUSG00000027955 | Fam198b | 2.37 | 1.16E-06 | 3.08 | 2.37E-11 | 3.07 | 3.65E-11 |
| ENSMUSG00000051517 | Arhgef39 | 2.36 | 4.77E-06 | 3.26 | 2.45E-10 | 2.72 | 1.13E-07 |
| ENSMUSG00000022469 | Rapgef3 | 2.35 | 0.000601714 | 4.13 | 7.22E-10 | 4.9 | 6.28E-12 |
| ENSMUSG00000026278 | Bok | 2.35 | 3.87E-08 | 2.11 | 2.01E-06 | 2.07 | 5.42E-06 |
| ENSMUSG00000040428 | Plekha4 | 2.34 | 0.005037162 | 2.19 | 0.011014242 | 2.04 | 0.033410787 |
| ENSMUSG00000049115 | Agtr1a | 2.34 | 3.42E-05 | 19.85 | 1.98E-47 | 3.19 | 1.35E-08 |
| ENSMUSG00000067889 | Sptbn2 | 2.33 | 4.70E-06 | 3.62 | 2.15E-12 | 2.44 | 1.80E-06 |
| ENSMUSG00000020828 | Pld2 | 2.32 | 2.46E-07 | 3.5 | 2.93E-15 | 2.49 | 2.76E-08 |
| ENSMUSG00000028434 | Epb4.1l4b | 2.32 | 4.10E-07 | 4.04 | 1.77E-18 | 2.01 | 4.69E-05 |
| ENSMUSG00000030844 | Rgs10 | 2.32 | 6.25E-06 | 2.82 | 1.47E-08 | 2.6 | 3.88E-07 |
| ENSMUSG00000026546 | Ccdc19 | 2.31 | 0.008229566 | 4 | 1.76E-07 | 4.56 | 9.68E-09 |
| ENSMUSG00000041992 | Rapgef5 | 2.31 | 8.60E-06 | 2.87 | 6.17E-09 | 2.25 | 2.11E-05 |
| ENSMUSG00000021066 | Atl1 | 2.3 | 5.84E-06 | 2.36 | 1.73E-06 | 2.04 | 0.000161571 |
| ENSMUSG00000021485 | Mxd3 | 2.29 | 1.23E-05 | 3.43 | 3.76E-11 | 3.97 | 6.73E-14 |
| ENSMUSG00000034684 | Sema3f | 2.29 | 2.53E-05 | 2.47 | 4.37E-06 | 5.09 | 1.59E-16 |
| ENSMUSG00000035547 | Capn5 | 2.28 | 0.00537216 | 8.8 | 2.79E-14 | 6.74 | 1.76E-11 |
| ENSMUSG00000059327 | Eda | 2.28 | 0.005076693 | 3.29 | 4.79E-05 | 4.06 | 6.01E-07 |
| ENSMUSG00000009585 | Apobec3 | 2.27 | 3.35E-07 | 2.66 | 2.50E-10 | 2.06 | 1.03E-05 |
| ENSMUSG00000020704 | Asic2 | 2.27 | 4.60E-05 | 3.94 | 1.73E-13 | 2.07 | 0.000400641 |
| ENSMUSG00000044469 | Tnfaip8l1 | 2.27 | 0.000139744 | 4.15 | 3.24E-13 | 4.84 | 3.90E-16 |
| ENSMUSG00000018654 | Ikzf1 | 2.26 | 0.002031965 | 2.48 | 0.000270242 | 3.22 | 1.09E-06 |
| ENSMUSG00000020788 | Atp2a3 | 2.26 | 0.019107023 | 4.1 | 2.77E-06 | 4.47 | 1.10E-06 |
| ENSMUSG00000020183 | Cpm | 2.25 | 0.001466915 | 10.4 | 1.53E-22 | 4.88 | 4.48E-11 |
| ENSMUSG00000030098 | Grip2 | 2.25 | 0.009612904 | 2.78 | 0.000456842 | 2.11 | 0.03423883 |
| ENSMUSG00000005686 | Ampd3 | 2.24 | 0.001851024 | 2.6 | 0.000175073 | 6.83 | 2.62E-17 |
| ENSMUSG00000022505 | Emp2 | 2.24 | 5.30E-07 | 3.26 | 1.48E-13 | 2.82 | 2.03E-10 |
| ENSMUSG00000035314 | Gdpd5 | 2.24 | 0.001677591 | 4.48 | 6.29E-09 | 3.74 | 3.52E-07 |
| ENSMUSG00000041329 | Atp1b2 | 2.24 | 0.005280012 | 3.22 | 2.30E-05 | 2.33 | 0.005265838 |
| ENSMUSG00000049858 | Suox | 2.24 | 2.46E-05 | 4.49 | 4.59E-16 | 4.01 | 3.80E-14 |
| ENSMUSG00000040033 | Stat2 | 2.23 | 6.09E-08 | 2.58 | 1.89E-10 | 2.37 | 1.07E-08 |
| ENSMUSG00000086342 | Gm12932 | 2.23 | 0.019397663 | 2.74 | 0.000853962 | 2.18 | 0.022507575 |
| ENSMUSG00000090122 | Kcne1l | 2.23 | 0.022880309 | 8.33 | 2.53E-13 | 7.77 | 4.94E-12 |
| ENSMUSG00000027562 | Car2 | 2.21 | 1.17E-05 | 4.27 | 1.13E-18 | 2.41 | 3.60E-07 |
| ENSMUSG00000032565 | Nudt16 | 2.2 | 3.44E-06 | 3.22 | 1.04E-12 | 2.93 | 1.02E-10 |
| ENSMUSG00000036882 | Arhgap33 | 2.2 | 1.44E-07 | 3.39 | 4.06E-16 | 2.06 | 2.31E-06 |
| ENSMUSG00000042766 | Trim46 | 2.17 | 4.09E-05 | 2.63 | 2.18E-07 | 2.16 | 0.000103697 |
| ENSMUSG00000087142 | Gm12454 | 2.16 | 0.010209871 | 2.84 | 6.01E-05 | 3.24 | 8.25E-06 |
| ENSMUSG00000037820 | Tgm2 | 2.15 | 0.029892821 | 15.15 | 1.35E-15 | 7.84 | 8.12E-10 |
| ENSMUSG00000026110 | Mgat4a | 2.14 | 1.23E-05 | 2.47 | 3.80E-08 | 2.38 | 2.58E-07 |
| ENSMUSG00000038732 | Mboat1 | 2.12 | 0.00096383 | 10.84 | 6.14E-39 | 4.69 | 2.94E-16 |
| ENSMUSG00000021684 | Pde8b | 2.11 | 0.000131402 | 2.49 | 2.65E-06 | 10.31 | 1.24E-38 |
| ENSMUSG00000050549 | 5730508B09Rik | 2.1 | 0.023090639 | 3.23 | 2.18E-06 | 2.2 | 0.006639252 |
| ENSMUSG00000072594 | Gm16439 | 2.1 | 0.042978937 | 3.15 | 0.001103813 | 2.2 | 0.038032103 |
| ENSMUSG00000016262 | Sertad4 | 2.08 | 0.018204691 | 3.31 | 1.08E-05 | 3.36 | 1.10E-05 |
| ENSMUSG00000024064 | Galnt14 | 2.07 | 0.019529368 | 4.77 | 8.31E-10 | 3.05 | 0.00011223 |
| ENSMUSG00000024130 | Abca3 | 2.07 | 2.45E-06 | 4.65 | 2.26E-24 | 2.24 | 2.13E-07 |
| ENSMUSG00000010830 | Kdelr3 | 2.06 | 4.95E-06 | 3.46 | 7.40E-15 | 4.89 | 1.26E-23 |
| ENSMUSG00000047547 | Cltb | 2.06 | 2.11E-05 | 2.76 | 2.84E-10 | 2.23 | 2.28E-06 |
| ENSMUSG00000032718 | Mansc1 | 2.05 | 0.018167856 | 2.75 | 0.000194445 | 2.07 | 0.019134692 |
| ENSMUSG00000034485 | Uaca | 2.05 | 2.10E-06 | 2.71 | 1.15E-11 | 3.13 | 8.84E-15 |
| ENSMUSG00000056481 | Cd248 | 2.05 | 0.000212079 | 3.02 | 1.60E-09 | 13.88 | 1.66E-46 |
| ENSMUSG00000025810 | Nrp1 | 2.04 | 0.024358181 | 2.46 | 0.003978657 | 10.01 | 4.90E-15 |
| ENSMUSG00000023224 | Serping1 | 2.03 | 0.000229876 | 6.66 | 8.18E-26 | 5.24 | 7.16E-20 |
| ENSMUSG00000019027 | Dnahc1 | 2.02 | 0.000128525 | 4.06 | 1.11E-16 | 3.24 | 1.17E-11 |
| ENSMUSG00000022358 | Fbxo32 | 2.02 | 0.037995701 | 3.52 | 1.24E-05 | 5.3 | 4.48E-09 |
| ENSMUSG00000048402 | Gli2 | 2.02 | 2.06E-05 | 3.95 | 3.35E-18 | 3.54 | 1.43E-15 |
| ENSMUSG00000069833 | Ahnak | 2.01 | 0.023238948 | 5.14 | 1.85E-09 | 3.03 | 0.000106942 |
| ENSMUSG00000032946 | Rasgrp2 | 2 | 0.000717426 | 2.58 | 3.94E-07 | 3 | 7.79E-09 |
| ENSMUSG00000059991 | Nptx2 | 2 | 0.001409346 | 3.03 | 1.59E-08 | 2.85 | 1.10E-07 |
