# Supplementary material for: In Vitro Generation of Neuromesodermal Progenitors Reveals Distinct Roles for Wnt Signalling in the Specification of Spinal Cord and Paraxial Mesoderm Identity
Source: PLoS Biol. 2014 Aug 26;12(8):e1001937. doi: 10.1371/journal.pbio.1001937 (PMC4144800; doi:10.1371/journal.pbio.1001937)
Supplement: Table S3 — List of the mesodermal specific genes. Pair wised comparisons identifies the genes upregulated in mesodermal conditions compared with all neuronal conditions at day 5. Genes are shown with their Ensembl gene_id number, short gene name and in each of the comparisons the fold change and p adjusted value is calculated using DESeq. (DOC) [file pbio.1001937.s008.doc]

| **ENSEMBL_GENE_ID** | **GENE_NAME** | **Fold change**  **MvsNA** | **padj**  **MvsNA** | **Fold change**  **MvsNH** | **padj.**  **MvsNH** | **Fold change**  **MvsNP** | **padj**  **MvsNP** |
| --- | --- | --- | --- | --- | --- | --- | --- |
| ENSMUSG00000000093 | Tbx2 | **4.71** | 3.39E-06 | **4.17** | 7.20E-06 | **5.47** | 2.13E-07 |
| ENSMUSG00000000142 | Axin2 | **5.73** | 1.53E-06 | **9.96** | 1.13E-10 | **9.15** | 6.47E-10 |
| ENSMUSG00000000706 | Btn1a1 | **23.02** | 4.37E-16 | **13.12** | 1.12E-15 | **8.07** | 3.63E-11 |
| ENSMUSG00000000782 | Tcf7 | **3.69** | 9.27E-19 | **4.26** | 8.69E-24 | **2.24** | 3.83E-08 |
| ENSMUSG00000001604 | Tcea3 | **4.51** | 4.29E-16 | **2.93** | 2.01E-10 | **2.98** | 2.33E-10 |
| ENSMUSG00000001741 | Il16 | **17.58** | 5.19E-11 | **13.71** | 1.26E-11 | **7.75** | 3.49E-08 |
| ENSMUSG00000001819 | Hoxd13 | **9.17** | 2.07E-09 | **15.07** | 4.19E-13 | **7.88** | 4.42E-09 |
| ENSMUSG00000001870 | Ltbp1 | **4.22** | 9.28E-08 | **3.46** | 1.88E-06 | **3.71** | 6.94E-07 |
| ENSMUSG00000001918 | Slc1a5 | **5.52** | 9.31E-29 | **2.09** | 7.79E-07 | **2.68** | 1.70E-11 |
| ENSMUSG00000001930 | Vwf | **4.29** | 0.000412459 | **2.83** | 0.009411343 | **2.36** | 0.046554532 |
| ENSMUSG00000002897 | Il17ra | **2.47** | 4.89E-08 | **2.39** | 5.39E-08 | **2.64** | 1.52E-09 |
| ENSMUSG00000003436 | Dll3 | **13.62** | 2.19E-10 | **23.28** | 7.50E-15 | **14.91** | 1.49E-11 |
| ENSMUSG00000003438 | Timm50 | **2.62** | 3.17E-10 | **2.64** | 3.29E-11 | **2.30** | 2.04E-08 |
| ENSMUSG00000005045 | Chd5 | **5.05** | 6.86E-15 | **2.23** | 4.44E-05 | **2.99** | 2.53E-08 |
| ENSMUSG00000005148 | Klf5 | **8.92** | 4.95E-15 | **4.44** | 1.30E-08 | **6.79** | 1.11E-12 |
| ENSMUSG00000005268 | Prlr | **7.35** | 2.77E-05 | **3.24** | 0.004660086 | **8.53** | 5.41E-06 |
| ENSMUSG00000005357 | Slc1a6 | **4.53** | 9.12E-16 | **3.23** | 2.61E-11 | **4.69** | 8.05E-18 |
| ENSMUSG00000005373 | Mlxipl | **12.67** | 8.74E-16 | **5.10** | 1.08E-08 | **4.20** | 4.96E-07 |
| ENSMUSG00000005503 | Evx1 | **311.42** | 1.64E-17 | **Inf** | 7.21E-25 | **1041.96** | 1.69E-21 |
| ENSMUSG00000006445 | Epha2 | **2.32** | 0.002293418 | **3.99** | 3.52E-08 | **2.21** | 0.003880609 |
| ENSMUSG00000006724 | Cyp27b1 | **5.88** | 1.38E-06 | **3.83** | 9.14E-05 | **5.01** | 9.11E-06 |
| ENSMUSG00000007097 | Atp1a2 | **20.27** | 3.07E-25 | **30.34** | 4.43E-33 | **10.96** | 9.48E-19 |
| ENSMUSG00000007107 | Atp1a4 | **26.25** | 7.59E-08 | **11.62** | 1.69E-07 | **9.89** | 1.97E-06 |
| ENSMUSG00000007440 | Pcdhac1 | **5.22** | 2.36E-21 | **11.76** | 1.66E-43 | **8.66** | 1.29E-34 |
| ENSMUSG00000007653 | Gabrb2 | **4.22** | 0.015738294 | **31.28** | 1.16E-08 | **15.51** | 3.83E-06 |
| ENSMUSG00000009376 | Met | **2.82** | 4.05E-11 | **2.17** | 1.21E-06 | **2.03** | 1.59E-05 |
| ENSMUSG00000009633 | G0s2 | **5.56** | 4.04E-19 | **5.22** | 1.14E-19 | **2.05** | 0.000153615 |
| ENSMUSG00000009900 | Wnt3a | **35.80** | 6.48E-11 | **48.84** | 1.49E-13 | **4.26** | 0.001946021 |
| ENSMUSG00000010122 | Slc47a1 | **5.29** | 2.97E-06 | **5.78** | 3.19E-07 | **4.79** | 7.31E-06 |
| ENSMUSG00000010760 | Phlda2 | **32.99** | 1.77E-09 | **25.55** | 4.31E-09 | **25.84** | 4.05E-09 |
| ENSMUSG00000011256 | Adam19 | **4.15** | 1.67E-13 | **4.88** | 1.73E-17 | **2.26** | 3.18E-05 |
| ENSMUSG00000011267 | Zfp296 | **5.76** | 2.89E-06 | **2.69** | 0.011518911 | **2.46** | 0.025728439 |
| ENSMUSG00000011463 | Cpb1 | **3.98** | 0.038471228 | **10.90** | 0.000141254 | **4.70** | 0.017039708 |
| ENSMUSG00000013089 | Etv5 | **3.33** | 3.97E-16 | **13.45** | 6.21E-61 | **3.37** | 1.93E-16 |
| ENSMUSG00000014361 | Mertk | **7.68** | 6.83E-33 | **5.54** | 1.04E-26 | **5.51** | 3.80E-26 |
| ENSMUSG00000014603 | Alx3 | **4.41** | 1.94E-12 | **8.73** | 1.41E-24 | **7.85** | 9.50E-22 |
| ENSMUSG00000014773 | Dll1 | **15.12** | 1.49E-48 | **9.49** | 2.90E-38 | **13.89** | 5.29E-50 |
| ENSMUSG00000015090 | Ptgds | **2.95** | 0.022991361 | **22.36** | 5.83E-10 | **15.44** | 3.96E-08 |
| ENSMUSG00000015354 | Pcolce2 | **3.27** | 1.23E-10 | **7.71** | 7.05E-28 | **3.67** | 3.00E-13 |
| ENSMUSG00000015396 | Cd83 | **12.76** | 2.16E-22 | **5.06** | 1.09E-12 | **2.94** | 2.05E-06 |
| ENSMUSG00000016128 | Stard13 | **2.03** | 2.95E-05 | **2.21** | 8.84E-07 | **2.04** | 1.93E-05 |
| ENSMUSG00000017697 | Ada | **3.45** | 3.68E-07 | **3.03** | 1.67E-06 | **2.25** | 0.000911662 |
| ENSMUSG00000017724 | Etv4 | **7.18** | 3.91E-25 | **115.80** | 2.45E-97 | **4.59** | 6.68E-17 |
| ENSMUSG00000018604 | Tbx3 | **14.39** | 4.51E-06 | **5.04** | 0.004664392 | **3.81** | 0.027188701 |
| ENSMUSG00000018774 | Cd68 | **4.25** | 5.10E-05 | **2.31** | 0.025790037 | **2.80** | 0.004020172 |
| ENSMUSG00000018973 | Hoxb1 | **34.07** | 8.68E-68 | **37.87** | 5.50E-75 | **6.08** | 5.64E-24 |
| ENSMUSG00000019235 | Rps6kl1 | **16.57** | 6.73E-05 | **13.24** | 0.000128776 | **13.63** | 0.000144945 |
| ENSMUSG00000019880 | Rspo3 | **16.93** | 1.05E-16 | **105.12** | 7.92E-35 | **11.80** | 9.99E-14 |
| ENSMUSG00000019913 | Sim1 | **7.78** | 1.13E-05 | **7.75** | 1.30E-05 | **5.93** | 0.000195181 |
| ENSMUSG00000020099 | Unc5b | **3.35** | 3.56E-15 | **2.60** | 1.13E-10 | **2.30** | 3.21E-08 |
| ENSMUSG00000020105 | Lrig3 | **3.92** | 6.33E-20 | **3.34** | 7.69E-17 | **4.50** | 2.31E-25 |
| ENSMUSG00000020142 | Slc1a4 | **4.59** | 3.53E-24 | **2.46** | 1.24E-09 | **2.93** | 1.22E-13 |
| ENSMUSG00000020154 | Ptprb | **8.72** | 3.41E-10 | **12.55** | 8.03E-14 | **3.12** | 0.000402998 |
| ENSMUSG00000020256 | Aldh1l2 | **8.35** | 5.30E-07 | **11.11** | 8.05E-09 | **6.31** | 1.00E-05 |
| ENSMUSG00000020275 | Rel | **4.78** | 7.45E-08 | **5.19** | 8.14E-09 | **4.25** | 9.07E-07 |
| ENSMUSG00000020303 | Stc2 | **3.52** | 8.00E-14 | **2.08** | 1.96E-05 | **2.00** | 6.21E-05 |
| ENSMUSG00000020524 | Gria1 | **8.37** | 4.85E-06 | **7.96** | 4.92E-06 | **30.64** | 9.52E-10 |
| ENSMUSG00000020541 | Tom1l1 | **2.61** | 3.01E-07 | **2.91** | 3.50E-09 | **3.71** | 5.10E-13 |
| ENSMUSG00000020566 | Atp6v1c2 | **3.84** | 0.000294257 | **5.40** | 9.78E-06 | **4.24** | 0.00025184 |
| ENSMUSG00000020608 | Smc6 | **2.17** | 2.76E-08 | **3.00** | 2.67E-14 | **2.85** | 3.92E-13 |
| ENSMUSG00000020889 | Nr1d1 | **2.15** | 0.000446059 | **2.63** | 1.85E-06 | **2.58** | 3.36E-06 |
| ENSMUSG00000021041 | 2700073G19Rik | **2.39** | 0.019924876 | **2.37** | 0.026624837 | **2.45** | 0.020978395 |
| ENSMUSG00000021071 | Trim9 | **5.07** | 0.003006501 | **3.45** | 0.029957579 | **12.25** | 4.29E-06 |
| ENSMUSG00000021091 | Serpina3n | **7.86** | 0.022001173 | **4.85** | 0.047928528 | **5.38** | 0.045757216 |
| ENSMUSG00000021098 | 4930447C04Rik | **3.19** | 6.26E-05 | **2.11** | 0.02933489 | **2.22** | 0.014149531 |
| ENSMUSG00000021136 | Smoc1 | **11.15** | 4.78E-46 | **5.90** | 3.51E-29 | **2.37** | 8.55E-08 |
| ENSMUSG00000021298 | Gpr132 | **6.04** | 0.010812162 | **21.23** | 3.82E-05 | **4.06** | 0.040516932 |
| ENSMUSG00000021319 | Sfrp4 | **2.80** | 0.002919206 | **3.66** | 9.42E-05 | **2.80** | 0.00256558 |
| ENSMUSG00000021458 | 2010111I01Rik | **2.91** | 2.48E-10 | **3.78** | 1.96E-16 | **2.30** | 4.61E-07 |
| ENSMUSG00000021469 | Msx2 | **7.98** | 0.020612249 | **16.62** | 0.001925295 | **13.18** | 0.005391606 |
| ENSMUSG00000021506 | Pitx1 | **6.61** | 0.014582829 | **7.92** | 0.004177878 | **9.50** | 0.003214576 |
| ENSMUSG00000021509 | Slc25a48 | **22.18** | 4.72E-05 | **14.14** | 5.92E-05 | **18.33** | 8.77E-05 |
| ENSMUSG00000021536 | Adcy2 | **5.92** | 1.01E-10 | **4.71** | 3.15E-09 | **5.02** | 1.02E-09 |
| ENSMUSG00000021567 | Nkd2 | **9.49** | 1.35E-23 | **8.76** | 3.48E-24 | **6.30** | 7.03E-18 |
| ENSMUSG00000021699 | Pde4d | **2.72** | 1.67E-09 | **2.97** | 7.65E-12 | **2.24** | 8.35E-07 |
| ENSMUSG00000021835 | Bmp4 | **6.13** | 8.09E-12 | **4.33** | 6.53E-09 | **2.08** | 0.006179589 |
| ENSMUSG00000021866 | Anxa11 | **4.63** | 2.59E-16 | **2.35** | 2.91E-06 | **4.07** | 7.64E-15 |
| ENSMUSG00000021953 | Tdh | **175.81** | 2.36E-26 | **5.28** | 3.05E-09 | **3.02** | 0.000204538 |
| ENSMUSG00000021974 | Fgf9 | **40.39** | 4.53E-07 | **44.78** | 1.73E-07 | **10.04** | 0.00100027 |
| ENSMUSG00000021991 | Cacna2d3 | **3.09** | 0.030105546 | **2.95** | 0.021064272 | **5.54** | 0.000273453 |
| ENSMUSG00000022039 | Adam2 | **46.14** | 8.44E-12 | **16.60** | 1.06E-09 | **3.83** | 0.00256207 |
| ENSMUSG00000022099 | Epb4.9 | **8.74** | 6.67E-36 | **2.77** | 1.50E-10 | **3.07** | 3.55E-12 |
| ENSMUSG00000022101 | Fgf17 | **50.59** | 2.37E-76 | **56.00** | 4.34E-86 | **67.70** | 1.75E-88 |
| ENSMUSG00000022221 | Ripk3 | **12.06** | 2.21E-41 | **5.06** | 8.40E-24 | **2.03** | 1.18E-05 |
| ENSMUSG00000022371 | Col14a1 | **258.92** | 0.001606403 | **79.79** | 0.005910231 | **26.75** | 0.038641398 |
| ENSMUSG00000022408 | Fam83f | **8.19** | 1.75E-15 | **5.10** | 2.37E-12 | **4.72** | 2.08E-10 |
| ENSMUSG00000022436 | Sh3bp1 | **2.85** | 2.19E-11 | **2.80** | 2.84E-12 | **2.29** | 4.40E-08 |
| ENSMUSG00000022439 | Parvg | **4.83** | 5.69E-06 | **5.23** | 2.31E-07 | **3.91** | 2.27E-05 |
| ENSMUSG00000022449 | Adamts20 | **4.94** | 0.000189407 | **5.46** | 7.03E-05 | **5.08** | 0.000188682 |
| ENSMUSG00000022508 | Bcl6 | **4.51** | 1.73E-13 | **3.28** | 7.53E-10 | **2.86** | 7.40E-08 |
| ENSMUSG00000022595 | Lypd2 | **62.16** | 1.18E-05 | **139.76** | 1.22E-06 | **100.65** | 4.41E-06 |
| ENSMUSG00000022665 | Ccdc80 | **5.67** | 4.36E-06 | **4.28** | 9.33E-05 | **2.35** | 0.036465996 |
| ENSMUSG00000022715 | Cldn26 | **5.80** | 0.011738886 | **5.45** | 0.016029927 | **10.02** | 0.003173308 |
| ENSMUSG00000022817 | Itgb5 | **2.90** | 6.15E-09 | **4.05** | 3.36E-16 | **2.66** | 1.97E-08 |
| ENSMUSG00000022861 | Dgkg | **5.40** | 4.07E-07 | **4.48** | 1.48E-06 | **8.54** | 3.57E-10 |
| ENSMUSG00000023031 | Cela1 | **7.88** | 8.97E-05 | **6.35** | 0.00023129 | **4.58** | 0.003773928 |
| ENSMUSG00000023443 | Esx1 | **8.07** | 5.86E-05 | **9.43** | 1.19E-05 | **7.45** | 9.83E-05 |
| ENSMUSG00000023781 | Hes7 | **124.25** | 1.18E-20 | **253.72** | 8.04E-26 | **65.58** | 5.16E-18 |
| ENSMUSG00000023911 | Flywch2 | **4.24** | 1.07E-14 | **2.04** | 7.15E-05 | **2.04** | 0.000100249 |
| ENSMUSG00000023972 | Ptk7 | **4.33** | 3.42E-22 | **4.57** | 3.20E-26 | **3.54** | 1.40E-18 |
| ENSMUSG00000024044 | Epb4.1l3 | **2.97** | 2.99E-13 | **2.68** | 4.56E-11 | **3.22** | 3.01E-15 |
| ENSMUSG00000024049 | Myom1 | **5.34** | 3.30E-15 | **3.87** | 6.91E-12 | **2.87** | 1.63E-07 |
| ENSMUSG00000024084 | Qpct | **6.29** | 2.87E-09 | **3.69** | 5.90E-06 | **2.27** | 0.007616376 |
| ENSMUSG00000024261 | Syt4 | **2.00** | 0.00245635 | **6.31** | 3.30E-11 | **3.31** | 2.38E-05 |
| ENSMUSG00000024331 | Dsc2 | **7.05** | 6.28E-19 | **2.29** | 0.00016386 | **3.58** | 2.47E-09 |
| ENSMUSG00000024347 | Psd2 | **10.06** | 0.001073507 | **15.96** | 5.75E-05 | **6.00** | 0.012558701 |
| ENSMUSG00000024379 | Tslp | **11.68** | 0.000340419 | **19.20** | 5.75E-06 | **8.68** | 0.000709059 |
| ENSMUSG00000024427 | Spry4 | **6.44** | 3.74E-10 | **20.12** | 1.22E-21 | **4.65** | 1.85E-07 |
| ENSMUSG00000024518 | Rax | **11.47** | 0.017487325 | **27.82** | 0.001675106 | **59.97** | 0.000529537 |
| ENSMUSG00000024619 | Cdx1 | **314.46** | 2.39E-07 | **583.13** | 6.53E-09 | **39.15** | 0.000187488 |
| ENSMUSG00000024647 | Cbln2 | **2.39** | 0.001510553 | **5.62** | 3.17E-09 | **2.22** | 0.009088392 |
| ENSMUSG00000024713 | Pcsk5 | **3.84** | 1.27E-06 | **7.48** | 9.69E-14 | **2.81** | 0.000213887 |
| ENSMUSG00000024871 | Doc2g | **5.48** | 9.73E-11 | **4.08** | 1.57E-08 | **4.44** | 2.78E-09 |
| ENSMUSG00000024901 | Peli3 | **11.80** | 1.53E-25 | **18.18** | 4.03E-36 | **13.99** | 5.67E-31 |
| ENSMUSG00000025013 | Tll2 | **8.16** | 2.50E-05 | **2.57** | 0.028005483 | **45.63** | 3.75E-10 |
| ENSMUSG00000025064 | Col17a1 | **19.98** | 0.00047958 | **14.79** | 0.000773202 | **7.08** | 0.014095453 |
| ENSMUSG00000025081 | Tdrd1 | **17.66** | 1.54E-36 | **7.69** | 9.37E-26 | **5.58** | 1.71E-19 |
| ENSMUSG00000025082 | Vwa2 | **33.50** | 9.79E-85 | **62.40** | 5.12E-115 | **31.79** | 7.12E-90 |
| ENSMUSG00000025091 | Pnliprp2 | **3.21** | 0.00441775 | **9.51** | 7.37E-08 | **4.52** | 0.000181358 |
| ENSMUSG00000025140 | Pycr1 | **3.86** | 4.73E-09 | **4.45** | 2.16E-11 | **3.25** | 1.71E-07 |
| ENSMUSG00000025141 | Myadml2 | **Inf** | 0.000554593 | **16.51** | 0.002144184 | **5.59** | 0.048864281 |
| ENSMUSG00000025142 | Aspscr1 | **2.21** | 3.23E-07 | **2.19** | 1.33E-07 | **2.11** | 6.14E-07 |
| ENSMUSG00000025219 | Fgf8 | **5.06** | 1.33E-20 | **178.87** | 5.31E-110 | **23.48** | 4.13E-62 |
| ENSMUSG00000025255 | Zfhx4 | **4.11** | 2.14E-06 | **2.87** | 0.000552705 | **2.44** | 0.006153575 |
| ENSMUSG00000025403 | Shmt2 | **3.45** | 5.87E-17 | **2.35** | 3.37E-09 | **2.22** | 4.15E-08 |
| ENSMUSG00000025776 | Crispld1 | **20.26** | 2.70E-09 | **32.13** | 1.15E-11 | **4.59** | 0.002354384 |
| ENSMUSG00000025880 | Smad7 | **2.77** | 0.000487712 | **2.66** | 0.00147747 | **2.29** | 0.008191753 |
| ENSMUSG00000025915 | Sgk3 | **8.44** | 2.19E-23 | **3.16** | 4.95E-10 | **6.34** | 1.22E-20 |
| ENSMUSG00000025932 | Eya1 | **2.11** | 0.015010034 | **4.24** | 4.32E-07 | **5.04** | 1.84E-08 |
| ENSMUSG00000026167 | Wnt10a | **18.95** | 2.89E-06 | **21.03** | 3.24E-07 | **25.87** | 6.65E-07 |
| ENSMUSG00000026193 | Fn1 | **9.11** | 1.37E-16 | **3.43** | 1.89E-06 | **4.18** | 2.50E-08 |
| ENSMUSG00000026251 | Chrnd | **8.58** | 0.000427578 | **7.26** | 0.000303043 | **3.90** | 0.013360783 |
| ENSMUSG00000026255 | Efhd1 | **3.06** | 0.005566154 | **4.87** | 2.49E-05 | **5.06** | 2.90E-05 |
| ENSMUSG00000026315 | Serpinb8 | **8.28** | 3.10E-05 | **2.46** | 0.046934968 | **3.85** | 0.004410752 |
| ENSMUSG00000026418 | Tnni1 | **17.00** | 0.00119057 | **5.66** | 0.028423225 | **7.12** | 0.020660717 |
| ENSMUSG00000026432 | Avpr1b | **7.63** | 2.57E-07 | **7.07** | 6.25E-08 | **14.27** | 1.44E-10 |
| ENSMUSG00000026437 | Cdk18 | **2.87** | 7.94E-10 | **3.33** | 5.28E-13 | **2.15** | 7.13E-06 |
| ENSMUSG00000026447 | Pik3c2b | **3.04** | 8.54E-06 | **2.85** | 3.21E-05 | **4.11** | 1.24E-08 |
| ENSMUSG00000026494 | Kif26b | **11.49** | 2.52E-08 | **5.15** | 0.000129982 | **6.20** | 2.14E-05 |
| ENSMUSG00000026497 | Mixl1 | **29.41** | 0.000878778 | **106.17** | 2.64E-05 | **63.77** | 0.000121296 |
| ENSMUSG00000026532 | Spta1 | **11.59** | 2.26E-08 | **6.33** | 6.39E-07 | **2.77** | 0.006380751 |
| ENSMUSG00000026768 | Itga8 | **5.91** | 2.86E-07 | **3.34** | 0.000406368 | **3.20** | 0.000797917 |
| ENSMUSG00000026815 | Gfi1b | **14.07** | 0.001102189 | **16.87** | 0.000130734 | **4.16** | 0.038149914 |
| ENSMUSG00000026923 | Notch1 | **5.77** | 1.99E-30 | **3.35** | 8.59E-17 | **4.81** | 5.96E-27 |
| ENSMUSG00000027004 | Frzb | **13.09** | 2.17E-10 | **17.80** | 3.95E-13 | **10.36** | 2.12E-09 |
| ENSMUSG00000027009 | Itga4 | **4.46** | 0.001188472 | **2.62** | 0.04373858 | **4.17** | 0.003265626 |
| ENSMUSG00000027016 | Zfp385b | **6.14** | 4.01E-15 | **6.33** | 8.13E-16 | **6.37** | 2.50E-15 |
| ENSMUSG00000027071 | P2rx3 | **9.53** | 8.40E-11 | **3.22** | 0.000533763 | **2.34** | 0.019824075 |
| ENSMUSG00000027102 | Hoxd8 | **35.12** | 0.002996489 | **66.09** | 0.0005235 | **11.27** | 0.046802901 |
| ENSMUSG00000027200 | Sema6d | **2.61** | 0.009256784 | **2.20** | 0.03574181 | **2.44** | 0.017575131 |
| ENSMUSG00000027340 | Slc23a2 | **3.75** | 2.05E-14 | **2.39** | 3.20E-07 | **3.41** | 1.20E-12 |
| ENSMUSG00000027456 | Sdcbp2 | **4.95** | 1.93E-09 | **2.63** | 4.96E-05 | **2.42** | 0.000376254 |
| ENSMUSG00000027457 | Snph | **11.64** | 1.90E-10 | **7.89** | 9.54E-09 | **10.96** | 9.53E-11 |
| ENSMUSG00000027765 | P2ry1 | **12.30** | 4.91E-35 | **9.49** | 7.43E-32 | **10.91** | 8.00E-34 |
| ENSMUSG00000027859 | Ngf | **17.95** | 3.25E-18 | **8.80** | 1.40E-14 | **7.50** | 1.91E-12 |
| ENSMUSG00000027863 | Cd2 | **15.75** | 0.00377152 | **47.90** | 0.000147161 | **51.12** | 0.000181358 |
| ENSMUSG00000027894 | Slc6a17 | **6.87** | 0.000396564 | **9.31** | 2.28E-05 | **20.73** | 7.24E-08 |
| ENSMUSG00000027895 | Kcnc4 | **10.34** | 5.22E-09 | **8.55** | 8.75E-10 | **8.44** | 6.75E-09 |
| ENSMUSG00000027947 | Il6ra | **10.80** | 2.88E-08 | **14.35** | 5.60E-10 | **12.46** | 5.24E-09 |
| ENSMUSG00000027954 | Efna1 | **14.00** | 1.33E-12 | **7.81** | 3.65E-09 | **2.41** | 0.020727804 |
| ENSMUSG00000027956 | Tmem144 | **3.39** | 2.01E-08 | **2.27** | 0.000126976 | **2.95** | 2.73E-07 |
| ENSMUSG00000027962 | Vcam1 | **5.30** | 2.08E-09 | **2.38** | 0.002456242 | **3.16** | 2.32E-05 |
| ENSMUSG00000027985 | Lef1 | **10.66** | 2.36E-26 | **102.44** | 4.12E-74 | **9.09** | 5.80E-24 |
| ENSMUSG00000027994 | Ccdc109b | **11.33** | 0.001760431 | **15.24** | 0.000285392 | **9.91** | 0.002340272 |
| ENSMUSG00000028033 | Kcnq5 | **2.90** | 3.28E-07 | **5.75** | 4.27E-18 | **3.84** | 2.81E-11 |
| ENSMUSG00000028039 | Efna3 | **2.90** | 2.22E-11 | **9.54** | 9.95E-45 | **2.92** | 2.43E-12 |
| ENSMUSG00000028132 | Tmem56 | **6.58** | 1.93E-05 | **8.03** | 1.43E-06 | **15.98** | 5.23E-09 |
| ENSMUSG00000028159 | Dapp1 | **2.44** | 1.75E-05 | **5.34** | 9.83E-18 | **2.19** | 6.59E-05 |
| ENSMUSG00000028179 | Cth | **3.33** | 2.27E-12 | **7.49** | 2.09E-30 | **4.48** | 2.08E-18 |
| ENSMUSG00000028496 | Mllt3 | **2.99** | 7.16E-08 | **7.25** | 2.93E-22 | **3.40** | 1.53E-09 |
| ENSMUSG00000028528 | Dnajc6 | **3.71** | 0.001821821 | **7.31** | 1.27E-06 | **7.74** | 1.55E-06 |
| ENSMUSG00000028542 | Slc6a9 | **5.64** | 4.38E-27 | **2.54** | 1.12E-09 | **2.84** | 1.03E-11 |
| ENSMUSG00000028602 | Tnfrsf8 | **Inf** | 1.13E-06 | **12.15** | 3.73E-05 | **9.57** | 0.000231728 |
| ENSMUSG00000028621 | Cyb5rl | **2.59** | 8.60E-09 | **2.03** | 1.41E-05 | **2.53** | 1.09E-08 |
| ENSMUSG00000028766 | Alpl | **10.98** | 9.12E-51 | **8.12** | 1.36E-43 | **5.56** | 6.20E-31 |
| ENSMUSG00000028838 | Extl1 | **3.98** | 8.16E-13 | **2.43** | 1.24E-06 | **3.39** | 4.27E-11 |
| ENSMUSG00000028862 | Map3k6 | **6.30** | 2.20E-11 | **2.06** | 0.008596442 | **2.48** | 0.001276812 |
| ENSMUSG00000028894 | Inpp5b | **2.36** | 2.61E-08 | **2.03** | 3.37E-06 | **2.23** | 1.25E-07 |
| ENSMUSG00000028949 | Smarcd3 | **2.31** | 0.003374036 | **2.75** | 0.000195001 | **2.37** | 0.002857686 |
| ENSMUSG00000029219 | Slc10a4 | **3.25** | 0.045470516 | **7.41** | 0.001304193 | **7.84** | 0.001920909 |
| ENSMUSG00000029245 | Epha5 | **10.19** | 0.007476943 | **34.87** | 4.51E-05 | **9.56** | 0.010056909 |
| ENSMUSG00000029314 | Agpat9 | **5.33** | 0.000413515 | **5.58** | 0.000250594 | **3.30** | 0.016136154 |
| ENSMUSG00000029361 | Nos1 | **3.00** | 3.86E-06 | **2.59** | 2.58E-05 | **4.25** | 7.81E-10 |
| ENSMUSG00000029410 | Ppef2 | **16.34** | 5.17E-32 | **37.22** | 2.58E-47 | **6.37** | 7.64E-19 |
| ENSMUSG00000029413 | Naaa | **16.30** | 8.97E-36 | **21.61** | 2.38E-44 | **12.41** | 2.29E-32 |
| ENSMUSG00000029468 | P2rx7 | **2.98** | 0.019253785 | **5.39** | 8.23E-05 | **5.25** | 0.000171584 |
| ENSMUSG00000029544 | Cabp1 | **9.78** | 9.42E-34 | **4.20** | 3.67E-18 | **2.31** | 5.64E-07 |
| ENSMUSG00000029646 | Cdx2 | **22.61** | 1.73E-10 | **939.37** | 1.32E-30 | **8.47** | 4.69E-06 |
| ENSMUSG00000029752 | Asns | **2.63** | 5.21E-11 | **2.55** | 9.67E-11 | **2.09** | 5.14E-07 |
| ENSMUSG00000029797 | Sspo | **5.57** | 9.23E-07 | **2.20** | 0.032949096 | **2.36** | 0.018321576 |
| ENSMUSG00000029816 | Gpnmb | **42.66** | 1.75E-42 | **10.03** | 5.83E-27 | **5.03** | 5.72E-15 |
| ENSMUSG00000029830 | Svopl | **166.62** | 1.21E-11 | **148.97** | 1.14E-12 | **369.22** | 8.12E-14 |
| ENSMUSG00000029832 | Nfe2l3 | **2.83** | 1.93E-05 | **3.15** | 2.84E-07 | **4.47** | 2.76E-10 |
| ENSMUSG00000029859 | Epha1 | **5.84** | 6.99E-29 | **3.59** | 9.60E-18 | **3.58** | 3.06E-17 |
| ENSMUSG00000029924 | Slc37a3 | **2.20** | 2.14E-07 | **2.23** | 5.31E-08 | **2.08** | 1.01E-06 |
| ENSMUSG00000030020 | Prickle2 | **12.40** | 5.34E-07 | **8.66** | 7.81E-06 | **5.94** | 0.000322496 |
| ENSMUSG00000030022 | Adamts9 | **4.95** | 9.01E-12 | **5.42** | 2.00E-13 | **2.79** | 1.62E-05 |
| ENSMUSG00000030111 | A2m | **4.80** | 3.68E-08 | **2.66** | 0.000630712 | **3.05** | 0.000123131 |
| ENSMUSG00000030247 | Kcnj8 | **Inf** | 6.14E-07 | **43.71** | 1.33E-06 | **17.65** | 6.07E-05 |
| ENSMUSG00000030249 | Abcc9 | **30.50** | 2.89E-05 | **20.09** | 4.71E-05 | **5.69** | 0.015452557 |
| ENSMUSG00000030406 | Gipr | **2.58** | 0.002416793 | **2.54** | 0.002946936 | **5.21** | 1.30E-07 |
| ENSMUSG00000030544 | Mesp1 | **56.80** | 0.000688204 | **422.76** | 4.13E-06 | **259.98** | 1.70E-05 |
| ENSMUSG00000030699 | Tbx6 | **501.80** | 4.58E-35 | **558.55** | 1.17E-38 | **332.04** | 5.81E-35 |
| ENSMUSG00000030768 | Disp1 | **4.62** | 2.09E-23 | **2.92** | 4.03E-13 | **3.73** | 7.51E-19 |
| ENSMUSG00000030825 | Hsd17b14 | **5.68** | 2.66E-09 | **2.04** | 0.014641406 | **2.21** | 0.007010038 |
| ENSMUSG00000030827 | Fgf21 | **40.94** | 0.000810095 | **4.85** | 0.031882931 | **8.59** | 0.009375949 |
| ENSMUSG00000030834 | Abcc6 | **6.58** | 4.92E-09 | **3.06** | 0.000200411 | **6.69** | 6.65E-10 |
| ENSMUSG00000030862 | Cpxm2 | **9.53** | 1.24E-11 | **37.81** | 1.85E-20 | **7.01** | 1.34E-09 |
| ENSMUSG00000031026 | Trim66 | **6.28** | 0.000554593 | **8.53** | 6.05E-05 | **12.39** | 7.23E-06 |
| ENSMUSG00000031129 | Slc9a9 | **4.62** | 1.80E-06 | **4.38** | 1.82E-07 | **2.32** | 0.003221819 |
| ENSMUSG00000031137 | Fgf13 | **2.21** | 3.18E-07 | **4.43** | 6.29E-23 | **3.09** | 6.90E-14 |
| ENSMUSG00000031146 | Plp2 | **4.45** | 1.21E-21 | **2.79** | 7.06E-12 | **3.13** | 2.51E-14 |
| ENSMUSG00000031214 | Ophn1 | **2.59** | 1.13E-09 | **2.35** | 2.18E-08 | **3.10** | 8.17E-14 |
| ENSMUSG00000031253 | Srpx2 | **11.40** | 1.05E-05 | **4.43** | 0.002310818 | **3.31** | 0.018938306 |
| ENSMUSG00000031297 | Slc7a3 | **6.46** | 4.43E-32 | **2.81** | 4.97E-11 | **3.79** | 7.60E-18 |
| ENSMUSG00000031326 | Cdx4 | **545.26** | 3.24E-07 | **Inf** | 5.18E-11 | **13.79** | 0.012231594 |
| ENSMUSG00000031391 | L1cam | **15.90** | 1.27E-13 | **4.61** | 6.50E-06 | **10.13** | 3.36E-11 |
| ENSMUSG00000031523 | Dlc1 | **6.00** | 4.44E-10 | **2.82** | 0.000263404 | **2.85** | 0.000290478 |
| ENSMUSG00000031659 | Adcy7 | **2.97** | 8.52E-08 | **2.15** | 0.000102572 | **3.47** | 3.96E-10 |
| ENSMUSG00000031661 | Nkd1 | **10.17** | 0.002298938 | **24.59** | 2.28E-05 | **9.11** | 0.003766208 |
| ENSMUSG00000031665 | Sall1 | **2.19** | 1.13E-07 | **2.64** | 2.80E-11 | **2.35** | 6.89E-09 |
| ENSMUSG00000031870 | Pgr | **3.19** | 4.21E-09 | **3.36** | 3.21E-10 | **3.99** | 8.52E-12 |
| ENSMUSG00000031906 | Smpd3 | **11.22** | 0.034437074 | **11.97** | 0.028265725 | **16.02** | 0.017894154 |
| ENSMUSG00000032011 | Thy1 | **7.55** | 3.87E-23 | **4.03** | 5.69E-13 | **4.73** | 9.59E-16 |
| ENSMUSG00000032076 | Cadm1 | **2.76** | 8.75E-13 | **3.21** | 6.67E-16 | **4.11** | 8.25E-23 |
| ENSMUSG00000032092 | Mpzl2 | **13.61** | 5.40E-19 | **4.91** | 5.16E-09 | **5.81** | 1.97E-10 |
| ENSMUSG00000032327 | Stra6 | **10.87** | 7.08E-27 | **3.21** | 1.67E-08 | **2.67** | 3.84E-06 |
| ENSMUSG00000032368 | Zic1 | **4.95** | 0.001555745 | **13.03** | 3.31E-07 | **6.22** | 0.000319093 |
| ENSMUSG00000032418 | Me1 | **2.27** | 1.71E-07 | **2.17** | 3.97E-07 | **2.93** | 6.88E-12 |
| ENSMUSG00000032601 | Prkar2a | **2.06** | 8.54E-07 | **2.26** | 2.00E-08 | **2.23** | 9.32E-08 |
| ENSMUSG00000032656 | 01/03/03 | **6.08** | 0.010204856 | **7.00** | 0.00477248 | **6.62** | 0.008534103 |
| ENSMUSG00000032698 | Lmo2 | **130.72** | 4.66E-48 | **204.29** | 5.65E-58 | **11.66** | 6.03E-19 |
| ENSMUSG00000032715 | Trib3 | **8.43** | 2.13E-14 | **2.71** | 0.000335913 | **2.25** | 0.004400832 |
| ENSMUSG00000032852 | Rspo4 | **6.62** | 5.25E-06 | **5.62** | 1.37E-05 | **3.05** | 0.007655011 |
| ENSMUSG00000032899 | Styk1 | **28.60** | 4.18E-08 | **41.39** | 4.33E-10 | **30.97** | 1.02E-08 |
| ENSMUSG00000033063 | Cntnap3 | **4.88** | 0.004019676 | **10.21** | 2.25E-05 | **10.53** | 3.33E-05 |
| ENSMUSG00000033102 | Cdc14b | **2.91** | 3.17E-13 | **4.93** | 2.17E-27 | **3.83** | 4.85E-20 |
| ENSMUSG00000033147 | Slc22a15 | **6.39** | 5.95E-09 | **4.85** | 2.22E-07 | **3.10** | 0.000321324 |
| ENSMUSG00000033227 | Wnt6 | **55.19** | 7.21E-27 | **50.40** | 4.26E-28 | **10.57** | 2.84E-13 |
| ENSMUSG00000033256 | Shf | **3.67** | 1.11E-10 | **4.40** | 2.36E-14 | **2.86** | 8.43E-08 |
| ENSMUSG00000033278 | Ptprm | **7.69** | 1.78E-08 | **2.75** | 0.005591763 | **3.80** | 0.000168393 |
| ENSMUSG00000033420 | Antxr1 | **4.01** | 8.57E-19 | **3.15** | 2.28E-14 | **2.92** | 2.56E-12 |
| ENSMUSG00000033427 | Upb1 | **2.14** | 0.02804985 | **2.42** | 0.016864735 | **2.34** | 0.028051655 |
| ENSMUSG00000033717 | Adra2a | **5.88** | 8.47E-07 | **4.61** | 1.11E-05 | **4.00** | 8.27E-05 |
| ENSMUSG00000033763 | Mtss1l | **2.36** | 1.12E-08 | **2.04** | 2.28E-06 | **2.12** | 8.03E-07 |
| ENSMUSG00000033769 | Exoc6b | **3.15** | 3.04E-14 | **2.30** | 1.90E-08 | **2.79** | 4.42E-12 |
| ENSMUSG00000033774 | Npbwr1 | **88.22** | 0.000150115 | **1001.59** | 1.45E-07 | **474.03** | 1.16E-06 |
| ENSMUSG00000033909 | Usp36 | **2.93** | 4.42E-13 | **2.17** | 1.60E-07 | **2.38** | 4.52E-09 |
| ENSMUSG00000034057 | Myrfl | **17.09** | 3.23E-12 | **36.23** | 9.55E-18 | **7.69** | 4.95E-09 |
| ENSMUSG00000034177 | Rnf43 | **3.44** | 6.23E-06 | **2.28** | 0.00187071 | **4.24** | 3.18E-08 |
| ENSMUSG00000034271 | Jdp2 | **5.15** | 2.24E-14 | **2.43** | 4.31E-05 | **3.01** | 1.64E-07 |
| ENSMUSG00000034324 | Tmem132c | **4.41** | 2.57E-07 | **16.76** | 4.87E-22 | **3.26** | 3.86E-05 |
| ENSMUSG00000034402 | Kcnh5 | **2.88** | 0.009416706 | **13.32** | 1.04E-09 | **32.20** | 2.90E-11 |
| ENSMUSG00000034486 | Gbx2 | **6.94** | 0.000363954 | **13.12** | 1.62E-06 | **5.28** | 0.002249215 |
| ENSMUSG00000034936 | Arl4d | **4.40** | 3.00E-08 | **4.10** | 3.75E-08 | **3.50** | 1.70E-06 |
| ENSMUSG00000035245 | Eogt | **10.01** | 2.61E-30 | **10.10** | 2.32E-32 | **8.10** | 2.48E-27 |
| ENSMUSG00000035283 | Adrb1 | **5.87** | 0.000172452 | **4.02** | 0.000812661 | **5.83** | 8.03E-05 |
| ENSMUSG00000035357 | Pdzrn3 | **6.85** | 1.24E-34 | **7.77** | 1.23E-41 | **5.22** | 6.73E-28 |
| ENSMUSG00000035692 | Isg15 | **8.86** | 8.14E-06 | **4.31** | 0.000631335 | **3.59** | 0.003880609 |
| ENSMUSG00000036040 | Adamtsl2 | **17.84** | 2.35E-11 | **5.38** | 2.77E-06 | **11.10** | 3.08E-10 |
| ENSMUSG00000036111 | Lmo1 | **3.88** | 0.008179278 | **4.48** | 0.003390903 | **14.10** | 3.94E-07 |
| ENSMUSG00000036492 | Rnf39 | **4.60** | 7.59E-06 | **2.30** | 0.007735038 | **3.36** | 0.000433855 |
| ENSMUSG00000036972 | Zic4 | **4.53** | 0.001688706 | **13.85** | 1.31E-07 | **5.12** | 0.000828929 |
| ENSMUSG00000037016 | Frem2 | **6.24** | 1.83E-06 | **2.90** | 0.005068703 | **5.98** | 1.55E-06 |
| ENSMUSG00000037035 | Inhbb | **11.03** | 1.19E-12 | **7.09** | 3.94E-10 | **4.00** | 6.35E-06 |
| ENSMUSG00000037188 | Grhl3 | **2.85** | 1.37E-06 | **8.60** | 5.18E-25 | **12.48** | 2.93E-32 |
| ENSMUSG00000037335 | Hand1 | **5.40** | 2.72E-06 | **3.94** | 8.08E-05 | **5.70** | 7.57E-06 |
| ENSMUSG00000037347 | Chst7 | **4.19** | 9.08E-14 | **2.43** | 1.39E-06 | **2.17** | 5.32E-05 |
| ENSMUSG00000037580 | Gch1 | **3.42** | 2.41E-05 | **4.14** | 7.70E-07 | **3.15** | 0.000100905 |
| ENSMUSG00000037686 | Aspg | **2.78** | 1.35E-05 | **3.90** | 5.88E-09 | **2.45** | 0.000232244 |
| ENSMUSG00000037736 | Limch1 | **4.66** | 2.40E-07 | **3.52** | 1.57E-05 | **3.90** | 2.98E-06 |
| ENSMUSG00000037771 | Slc32a1 | **2.49** | 0.023015252 | **4.71** | 0.000174601 | **7.46** | 1.75E-05 |
| ENSMUSG00000037994 | Slc9b2 | **3.42** | 1.62E-05 | **2.53** | 0.001414168 | **2.92** | 0.000237634 |
| ENSMUSG00000038009 | Dnajc22 | **5.26** | 1.03E-20 | **2.96** | 9.58E-11 | **3.36** | 5.43E-13 |
| ENSMUSG00000038037 | Socs1 | **2.89** | 3.68E-08 | **3.99** | 3.99E-13 | **2.77** | 1.04E-07 |
| ENSMUSG00000038068 | Rnf144b | **4.63** | 6.04E-19 | **3.80** | 2.76E-16 | **3.66** | 4.76E-15 |
| ENSMUSG00000038112 | AW551984 | **3.35** | 1.90E-05 | **4.55** | 1.03E-08 | **3.33** | 1.17E-05 |
| ENSMUSG00000038128 | Camk4 | **2.99** | 6.94E-11 | **4.34** | 1.74E-19 | **2.05** | 1.45E-05 |
| ENSMUSG00000038175 | Mylip | **16.02** | 1.01E-07 | **9.48** | 6.44E-06 | **4.80** | 0.00256558 |
| ENSMUSG00000038264 | Sema7a | **4.19** | 2.93E-12 | **3.66** | 7.59E-12 | **2.36** | 1.34E-05 |
| ENSMUSG00000038279 | Nop2 | **2.66** | 3.89E-11 | **2.13** | 2.11E-07 | **2.04** | 1.21E-06 |
| ENSMUSG00000038508 | Gdf15 | **9.02** | 7.56E-06 | **2.77** | 0.00947427 | **2.49** | 0.03423883 |
| ENSMUSG00000038530 | Rgs4 | **15.59** | 3.26E-07 | **7.76** | 8.63E-05 | **20.54** | 2.02E-08 |
| ENSMUSG00000038539 | Atf5 | **4.97** | 3.88E-15 | **3.34** | 1.15E-09 | **3.51** | 2.08E-10 |
| ENSMUSG00000038550 | Gm129 | **3.88** | 7.67E-14 | **2.91** | 7.95E-10 | **2.81** | 4.62E-09 |
| ENSMUSG00000038572 | Bpifb5 | **24.05** | 1.14E-08 | **10.92** | 1.94E-06 | **10.32** | 5.22E-06 |
| ENSMUSG00000038679 | Trps1 | **12.86** | 1.69E-07 | **11.01** | 4.06E-07 | **3.30** | 0.018390457 |
| ENSMUSG00000038879 | Nipal2 | **7.08** | 4.71E-06 | **2.90** | 0.012605174 | **3.20** | 0.006475396 |
| ENSMUSG00000039092 | Sptlc3 | **27.41** | 4.05E-05 | **61.08** | 1.86E-06 | **30.97** | 3.18E-05 |
| ENSMUSG00000039202 | Abhd2 | **3.28** | 5.08E-06 | **2.76** | 6.16E-05 | **3.08** | 1.06E-05 |
| ENSMUSG00000039316 | Rftn1 | **5.08** | 3.23E-26 | **8.69** | 5.28E-46 | **6.10** | 4.92E-34 |
| ENSMUSG00000039728 | Slc6a5 | **33.26** | 1.45E-05 | **32.03** | 4.77E-06 | **3.88** | 0.037490706 |
| ENSMUSG00000039908 | Slc26a11 | **3.70** | 4.04E-16 | **2.94** | 5.00E-12 | **3.62** | 1.61E-16 |
| ENSMUSG00000040026 | Saa3 | **61.83** | 1.58E-06 | **139.01** | 1.01E-07 | **17.18** | 8.45E-05 |
| ENSMUSG00000040118 | Cacna2d1 | **6.99** | 1.61E-09 | **7.43** | 4.46E-10 | **16.75** | 2.13E-17 |
| ENSMUSG00000040121 | Rep15 | **22.70** | 0.000374193 | **3.99** | 0.042264658 | **7.83** | 0.0049483 |
| ENSMUSG00000040260 | Daam2 | **11.87** | 1.29E-07 | **3.86** | 0.002769735 | **4.20** | 0.001665506 |
| ENSMUSG00000040296 | Ddx58 | **5.86** | 1.51E-24 | **3.83** | 1.51E-15 | **4.68** | 4.13E-20 |
| ENSMUSG00000040387 | Klhl32 | **3.56** | 4.00E-08 | **3.11** | 6.41E-08 | **4.63** | 1.63E-12 |
| ENSMUSG00000040433 | Zbtb38 | **3.57** | 3.29E-16 | **2.82** | 1.72E-11 | **3.30** | 1.01E-14 |
| ENSMUSG00000040435 | Ppp1r15a | **4.55** | 1.46E-05 | **2.26** | 0.02951397 | **3.15** | 0.001053648 |
| ENSMUSG00000040717 | Il17rd | **6.62** | 1.46E-07 | **15.30** | 5.78E-14 | **4.94** | 8.21E-06 |
| ENSMUSG00000041313 | Slc7a1 | **6.44** | 1.80E-30 | **2.49** | 9.73E-09 | **2.85** | 3.80E-11 |
| ENSMUSG00000041624 | Gucy1a2 | **2.22** | 0.000676855 | **2.33** | 0.000117625 | **2.52** | 4.05E-05 |
| ENSMUSG00000041782 | Lad1 | **32.18** | 1.24E-32 | **4.29** | 1.07E-09 | **7.14** | 1.61E-15 |
| ENSMUSG00000041930 | Fam222a | **4.63** | 3.27E-07 | **3.30** | 4.61E-05 | **7.37** | 1.68E-11 |
| ENSMUSG00000042064 | Myo3b | **20.47** | 2.15E-05 | **31.23** | 3.89E-07 | **29.35** | 2.13E-06 |
| ENSMUSG00000042102 | Dmgdh | **27.75** | 4.54E-30 | **66.84** | 8.94E-40 | **24.41** | 2.30E-30 |
| ENSMUSG00000042118 | Bhmt2 | **Inf** | 1.25E-17 | **31.10** | 2.34E-16 | **23.08** | 7.46E-14 |
| ENSMUSG00000042190 | Cmklr1 | **7.06** | 1.78E-17 | **2.37** | 8.72E-06 | **2.97** | 9.23E-08 |
| ENSMUSG00000042244 | Pglyrp3 | **93.87** | 0.002608517 | **Inf** | 3.05E-06 | **1475.87** | 3.21E-05 |
| ENSMUSG00000042284 | Itga1 | **8.62** | 2.29E-20 | **6.29** | 3.19E-17 | **3.30** | 4.35E-08 |
| ENSMUSG00000042417 | Ccno | **40.81** | 2.01E-11 | **7.08** | 1.34E-07 | **5.20** | 8.94E-06 |
| ENSMUSG00000042448 | Hoxd1 | **13.15** | 1.12E-21 | **74.26** | 7.57E-46 | **5.89** | 8.79E-13 |
| ENSMUSG00000042499 | Hoxd11 | **44.66** | 0.001780426 | **49.70** | 0.000382584 | **35.80** | 0.001108517 |
| ENSMUSG00000042501 | Cpa6 | **34.79** | 1.62E-05 | **56.93** | 6.76E-07 | **67.44** | 9.60E-07 |
| ENSMUSG00000042675 | Ypel3 | **2.60** | 0.003432738 | **2.81** | 0.001199549 | **2.10** | 0.032542208 |
| ENSMUSG00000042793 | Lgr6 | **7.11** | 1.99E-05 | **4.38** | 0.000139742 | **3.62** | 0.001665287 |
| ENSMUSG00000042810 | Krba1 | **5.96** | 3.00E-31 | **4.22** | 8.05E-23 | **4.93** | 2.97E-27 |
| ENSMUSG00000042942 | Greb1l | **3.57** | 8.90E-09 | **3.88** | 4.92E-10 | **6.88** | 7.29E-19 |
| ENSMUSG00000042988 | Notum | **157.45** | 6.25E-05 | **1280.09** | 8.53E-08 | **91.03** | 0.000179536 |
| ENSMUSG00000043017 | Ptgir | **10.93** | 8.36E-06 | **5.59** | 0.000139514 | **5.57** | 0.000196898 |
| ENSMUSG00000043165 | Lor | **7.78** | 0.035859731 | **12.51** | 0.008879962 | **26.27** | 0.000972698 |
| ENSMUSG00000043289 | Mei4 | **9.32** | 4.75E-20 | **4.36** | 2.46E-11 | **3.28** | 2.80E-08 |
| ENSMUSG00000044017 | Gpr133 | **40.27** | 2.71E-33 | **14.79** | 1.16E-25 | **4.64** | 3.72E-10 |
| ENSMUSG00000044033 | Ccdc141 | **21.16** | 1.11E-06 | **4.97** | 0.006016266 | **5.48** | 0.004625533 |
| ENSMUSG00000044034 | Npb | **4.79** | 0.000378337 | **2.74** | 0.016769523 | **3.22** | 0.005587091 |
| ENSMUSG00000044042 | Fmn1 | **4.78** | 6.84E-14 | **2.02** | 0.000436549 | **2.23** | 9.73E-05 |
| ENSMUSG00000044288 | Cnr1 | **19.77** | 6.87E-47 | **15.18** | 5.21E-44 | **14.09** | 1.12E-41 |
| ENSMUSG00000044352 | Sowaha | **36.78** | 1.83E-11 | **7.17** | 7.07E-07 | **9.94** | 8.55E-08 |
| ENSMUSG00000044734 | Serpinb1a | **34.03** | 6.34E-05 | **14.20** | 0.000432672 | **8.42** | 0.005201142 |
| ENSMUSG00000044835 | Ankrd45 | **3.23** | 7.84E-09 | **2.80** | 3.06E-07 | **2.34** | 2.99E-05 |
| ENSMUSG00000045087 | S1pr5 | **82.42** | 2.46E-20 | **99.16** | 1.10E-23 | **81.40** | 6.91E-22 |
| ENSMUSG00000045095 | Magi1 | **7.19** | 3.20E-38 | **6.69** | 4.84E-38 | **7.78** | 3.14E-43 |
| ENSMUSG00000045201 | Lrrc3b | **2.99** | 5.75E-08 | **5.16** | 3.95E-16 | **2.61** | 3.01E-06 |
| ENSMUSG00000045312 | Lhfpl2 | **5.29** | 5.95E-20 | **3.35** | 1.51E-12 | **2.95** | 1.10E-09 |
| ENSMUSG00000045348 | Nyap1 | **3.24** | 6.93E-11 | **2.65** | 1.31E-08 | **3.22** | 1.74E-11 |
| ENSMUSG00000045817 | Zfp36l2 | **2.20** | 2.74E-08 | **2.33** | 7.90E-09 | **2.08** | 1.31E-06 |
| ENSMUSG00000046085 | 4931422A03Rik | **11.07** | 1.64E-05 | **2.85** | 0.032105891 | **5.26** | 0.001171505 |
| ENSMUSG00000046318 | Ccbe1 | **40.51** | 3.09E-26 | **38.28** | 5.12E-28 | **17.77** | 4.49E-20 |
| ENSMUSG00000046687 | Gm5424 | **2.18** | 8.74E-07 | **2.40** | 5.92E-09 | **2.56** | 4.86E-10 |
| ENSMUSG00000046727 | Cystm1 | **2.98** | 9.76E-10 | **2.45** | 2.58E-07 | **3.23** | 1.92E-11 |
| ENSMUSG00000046750 | BC089491 | **3.26** | 2.44E-06 | **3.15** | 2.20E-06 | **2.41** | 0.000476139 |
| ENSMUSG00000046999 | 1110032F04Rik | **10.50** | 3.92E-18 | **14.24** | 1.53E-22 | **2.70** | 5.70E-05 |
| ENSMUSG00000047002 | Msgn1 | **12890.51** | 6.98E-17 | **Inf** | 9.04E-20 | **10332.47** | 2.49E-18 |
| ENSMUSG00000047443 | Fam132b | **4.52** | 9.13E-16 | **2.16** | 1.34E-05 | **3.08** | 1.43E-10 |
| ENSMUSG00000047495 | Dlgap2 | **3.55** | 0.01275385 | **3.90** | 0.009101383 | **14.52** | 1.48E-05 |
| ENSMUSG00000047562 | Mmp10 | **Inf** | 0.001742162 | **13.64** | 0.006871485 | **23.43** | 0.005752374 |
| ENSMUSG00000047636 | Cdcp2 | **175.25** | 3.26E-08 | **41.87** | 6.26E-07 | **55.73** | 2.48E-07 |
| ENSMUSG00000047643 | Gm5454 | **2.91** | 6.07E-07 | **12.95** | 1.76E-31 | **2.18** | 0.000309139 |
| ENSMUSG00000047842 | Diras2 | **10.95** | 2.35E-05 | **3.11** | 0.015005265 | **3.82** | 0.006532399 |
| ENSMUSG00000047959 | Kcna3 | **3.17** | 0.000222394 | **2.86** | 0.00055005 | **6.76** | 1.02E-09 |
| ENSMUSG00000048065 | Cyb5r2 | **15.62** | 9.05E-07 | **9.86** | 1.32E-05 | **7.00** | 0.000222188 |
| ENSMUSG00000048332 | Lhfp | **6.36** | 1.15E-27 | **7.37** | 5.57E-33 | **7.65** | 5.04E-34 |
| ENSMUSG00000048450 | Msx1 | **58.73** | 2.34E-05 | **166.15** | 1.65E-07 | **6.23** | 0.048492 |
| ENSMUSG00000048699 | 4732456N10Rik | **4.25** | 0.000308351 | **10.21** | 1.22E-08 | **2.57** | 0.016995949 |
| ENSMUSG00000048763 | Hoxb3 | **21.95** | 1.97E-27 | **6.61** | 4.69E-14 | **2.28** | 0.002846238 |
| ENSMUSG00000049252 | Lrp1b | **11.75** | 5.09E-06 | **60.15** | 3.44E-10 | **109.46** | 4.90E-10 |
| ENSMUSG00000049265 | Kcnk3 | **119.26** | 1.98E-17 | **49.02** | 9.56E-15 | **64.31** | 9.25E-16 |
| ENSMUSG00000049404 | Rarres1 | **8.31** | 0.001197326 | **12.58** | 7.93E-05 | **4.02** | 0.02997774 |
| ENSMUSG00000049556 | Lingo1 | **4.36** | 9.03E-20 | **5.75** | 8.37E-29 | **2.68** | 4.17E-10 |
| ENSMUSG00000049598 | Vsig8 | **5.52** | 0.007486476 | **4.30** | 0.014194163 | **3.91** | 0.032488653 |
| ENSMUSG00000049649 | Gpr3 | **13.44** | 2.64E-07 | **8.34** | 9.04E-07 | **6.72** | 1.13E-05 |
| ENSMUSG00000050010 | Shisa3 | **62.40** | 5.52E-09 | **36.68** | 7.06E-08 | **15.91** | 2.23E-05 |
| ENSMUSG00000050335 | Lgals3 | **6.86** | 4.73E-18 | **2.55** | 3.17E-06 | **2.57** | 4.46E-06 |
| ENSMUSG00000050447 | Lypd6 | **2.19** | 0.008014587 | **3.29** | 9.22E-05 | **3.94** | 5.50E-06 |
| ENSMUSG00000050663 | Trhde | **27.65** | 8.40E-05 | **25.45** | 2.27E-05 | **30.11** | 3.32E-05 |
| ENSMUSG00000050671 | Ism2 | **2.29** | 0.021448133 | **3.38** | 0.000365984 | **2.33** | 0.02621736 |
| ENSMUSG00000050917 | Fgf4 | **40.32** | 2.41E-69 | **14.59** | 1.30E-50 | **14.82** | 5.29E-50 |
| ENSMUSG00000051159 | Cited1 | **81.13** | 6.44E-10 | **55.41** | 1.87E-09 | **32.26** | 1.20E-07 |
| ENSMUSG00000051323 | Pcdh19 | **33.71** | 1.67E-18 | **199.63** | 1.20E-34 | **73.86** | 3.73E-26 |
| ENSMUSG00000051401 | Kctd16 | **48.15** | 5.01E-11 | **Inf** | 1.08E-15 | **39.39** | 2.15E-11 |
| ENSMUSG00000051413 | Plagl2 | **2.17** | 1.27E-05 | **2.09** | 2.96E-05 | **2.14** | 2.55E-05 |
| ENSMUSG00000051510 | Mafg | **2.01** | 1.47E-06 | **2.30** | 8.92E-09 | **2.45** | 7.43E-10 |
| ENSMUSG00000051851 | Cxx1c | **10.05** | 1.57E-25 | **8.10** | 8.19E-24 | **3.19** | 1.14E-08 |
| ENSMUSG00000052026 | Slc6a7 | **67.19** | 6.17E-07 | **10.39** | 1.31E-05 | **6.03** | 0.000811878 |
| ENSMUSG00000052396 | Mogat2 | **10.45** | 2.61E-20 | **7.24** | 1.99E-16 | **2.93** | 8.29E-06 |
| ENSMUSG00000052609 | Plekhg3 | **3.62** | 7.13E-17 | **2.55** | 2.72E-10 | **2.90** | 1.20E-12 |
| ENSMUSG00000052821 | Cysltr1 | **8.10** | 6.28E-08 | **3.08** | 0.002487001 | **3.76** | 0.000342779 |
| ENSMUSG00000053367 | Gm6792 | **7.90** | 5.26E-05 | **5.98** | 0.000319126 | **6.64** | 0.000419794 |
| ENSMUSG00000053613 | 4732444A12Rik | **90.05** | 1.38E-05 | **621.19** | 9.08E-09 | **74.45** | 1.18E-05 |
| ENSMUSG00000054580 | Pla2r1 | **10.26** | 1.83E-10 | **3.74** | 0.000163572 | **7.07** | 4.25E-08 |
| ENSMUSG00000054626 | Xlr | **8.22** | 0.000478228 | **16.58** | 8.88E-06 | **6.47** | 0.001107004 |
| ENSMUSG00000054988 | Agtr1b | **4.79** | 8.00E-05 | **28.78** | 6.09E-14 | **9.13** | 3.39E-08 |
| ENSMUSG00000055214 | Pld5 | **37.07** | 2.88E-07 | **90.66** | 4.23E-10 | **58.95** | 7.33E-09 |
| ENSMUSG00000055540 | Epha6 | **15.13** | 0.003043219 | **18.13** | 0.00056285 | **5.14** | 0.044117708 |
| ENSMUSG00000055555 | 4930502E18Rik | **13.58** | 1.11E-06 | **10.67** | 2.21E-06 | **5.33** | 0.000688935 |
| ENSMUSG00000055761 | Nkain3 | **6.64** | 2.79E-11 | **9.43** | 7.15E-16 | **5.42** | 7.09E-10 |
| ENSMUSG00000055780 | Usp26 | **12.38** | 1.52E-33 | **6.96** | 2.53E-25 | **3.94** | 4.39E-14 |
| ENSMUSG00000055891 | Ubl4b | **8.02** | 0.017699943 | **Inf** | 3.24E-05 | **12.05** | 0.007027229 |
| ENSMUSG00000055994 | Nod2 | **2.55** | 4.32E-05 | **6.63** | 1.09E-17 | **2.73** | 4.96E-06 |
| ENSMUSG00000056258 | Kcnq3 | **4.94** | 0.031910718 | **4.29** | 0.036850098 | **6.49** | 0.008236398 |
| ENSMUSG00000056856 | Jakmip3 | **45.79** | 0.001967071 | **46.75** | 0.001421499 | **30.97** | 0.004380883 |
| ENSMUSG00000057058 | Skap1 | **9.26** | 5.59E-16 | **4.00** | 4.01E-10 | **4.74** | 5.57E-11 |
| ENSMUSG00000057315 | Arhgap24 | **7.16** | 3.18E-16 | **2.97** | 1.92E-06 | **3.84** | 5.93E-09 |
| ENSMUSG00000057378 | Ryr3 | **18.04** | 0.036058772 | **33.93** | 0.013137919 | **62.01** | 0.005520877 |
| ENSMUSG00000057455 | Rit2 | **30.75** | 0.000366225 | **69.14** | 6.05E-05 | **10.62** | 0.006890503 |
| ENSMUSG00000057762 | Gm6169 | **4.58** | 2.57E-23 | **2.69** | 1.84E-11 | **2.94** | 1.75E-13 |
| ENSMUSG00000058099 | Nfam1 | **4.61** | 0.006912451 | **13.72** | 1.02E-06 | **33.93** | 5.36E-10 |
| ENSMUSG00000058297 | Spock2 | **4.67** | 2.77E-23 | **6.03** | 1.13E-33 | **4.62** | 4.88E-25 |
| ENSMUSG00000058400 | Qrfpr | **5.02** | 0.002894599 | **Inf** | 4.18E-09 | **43.46** | 4.44E-07 |
| ENSMUSG00000058571 | Gpc6 | **3.56** | 6.61E-07 | **2.15** | 0.003461775 | **2.54** | 0.000309166 |
| ENSMUSG00000058806 | Col13a1 | **28.72** | 2.31E-17 | **54.56** | 1.19E-24 | **4.69** | 1.55E-05 |
| ENSMUSG00000058897 | Col25a1 | **4.90** | 0.000291179 | **7.29** | 4.37E-06 | **2.52** | 0.047454675 |
| ENSMUSG00000059022 | Kcp | **7.27** | 1.80E-12 | **3.29** | 1.22E-05 | **3.57** | 3.04E-06 |
| ENSMUSG00000059334 | Zfp36l3 | **32.87** | 6.25E-14 | **2.08** | 0.037528835 | **7.25** | 2.67E-08 |
| ENSMUSG00000059901 | Adamts14 | **6.86** | 1.10E-23 | **3.19** | 1.56E-11 | **2.90** | 1.45E-09 |
| ENSMUSG00000059921 | Unc5c | **10.42** | 0.014788135 | **12.10** | 0.008335798 | **7.08** | 0.047176178 |
| ENSMUSG00000060180 | Myh13 | **11.91** | 7.01E-07 | **9.85** | 1.19E-06 | **5.32** | 0.000373582 |
| ENSMUSG00000060548 | Tnfrsf19 | **14.57** | 1.14E-09 | **13.64** | 6.25E-10 | **13.56** | 9.91E-10 |
| ENSMUSG00000060621 | Nkpd1 | **2.77** | 0.000453369 | **2.84** | 0.00026296 | **3.03** | 0.000167528 |
| ENSMUSG00000060961 | Slc4a4 | **3.22** | 0.000160268 | **2.33** | 0.007766518 | **2.53** | 0.004066725 |
| ENSMUSG00000061132 | Blnk | **2.56** | 0.017691318 | **12.57** | 3.52E-07 | **2.69** | 0.032875827 |
| ENSMUSG00000061143 | Maml3 | **2.93** | 2.04E-12 | **3.89** | 1.35E-19 | **2.80** | 1.45E-11 |
| ENSMUSG00000061603 | Akap6 | **2.25** | 0.030770707 | **4.72** | 2.52E-06 | **3.25** | 0.000576287 |
| ENSMUSG00000062327 | T | **7.49** | 4.70E-10 | **5.87** | 2.37E-08 | **11.85** | 3.99E-14 |
| ENSMUSG00000062372 | Otof | **5.22** | 0.007952911 | **13.37** | 4.64E-05 | **6.05** | 0.003937431 |
| ENSMUSG00000062542 | Syt9 | **9.17** | 7.71E-43 | **5.05** | 2.68E-26 | **5.49** | 1.14E-28 |
| ENSMUSG00000062609 | Kcnj15 | **2.78** | 0.002896005 | **6.30** | 2.22E-07 | **2.88** | 0.004073957 |
| ENSMUSG00000065663 | Gm22579 | **2.98** | 0.000196919 | **2.22** | 0.017710846 | **2.44** | 0.005325649 |
| ENSMUSG00000066842 | Hmcn1 | **26.34** | 2.52E-17 | **24.55** | 5.01E-18 | **10.80** | 2.99E-11 |
| ENSMUSG00000067341 | H2-Eb2 | **2.71** | 0.024792949 | **5.91** | 0.000205742 | **2.83** | 0.038641398 |
| ENSMUSG00000067704 | Wfdc13 | **17.19** | 0.000800774 | **40.73** | 4.54E-05 | **21.09** | 0.000410585 |
| ENSMUSG00000068323 | Slc4a5 | **3.60** | 4.11E-06 | **2.00** | 0.006560895 | **3.38** | 5.04E-06 |
| ENSMUSG00000070327 | Rnf213 | **24.72** | 2.11E-34 | **13.28** | 2.16E-26 | **17.59** | 2.62E-31 |
| ENSMUSG00000070366 | Ppapdc1a | **3.72** | 0.010210733 | **30.29** | 1.27E-06 | **4.79** | 0.004930489 |
| ENSMUSG00000070803 | Cited4 | **8.40** | 8.97E-15 | **8.40** | 2.40E-16 | **10.51** | 6.05E-18 |
| ENSMUSG00000070880 | Gad1 | **12.30** | 8.13E-22 | **48.63** | 7.54E-42 | **10.26** | 3.59E-21 |
| ENSMUSG00000070999 | Ccin | **6.06** | 0.000813766 | **2.83** | 0.031831765 | **12.03** | 8.99E-06 |
| ENSMUSG00000071068 | Treml2 | **18.34** | 6.56E-08 | **9.48** | 1.99E-07 | **5.36** | 0.000104258 |
| ENSMUSG00000071235 | Vrtn | **2.85** | 0.000320213 | **2.88** | 0.000281524 | **4.67** | 1.02E-07 |
| ENSMUSG00000071860 | 2900055J20Rik | **41.82** | 0.001147588 | **23.27** | 0.000749713 | **Inf** | 0.000111103 |
| ENSMUSG00000072294 | Klf12 | **7.04** | 6.53E-22 | **4.26** | 6.47E-16 | **2.96** | 5.95E-09 |
| ENSMUSG00000072662 | Mansc4 | **5.49** | 0.002623302 | **3.98** | 0.00776764 | **6.49** | 0.001088935 |
| ENSMUSG00000072952 | Gm5878 | **75.07** | 4.12E-07 | **9.18** | 4.93E-05 | **22.05** | 2.36E-06 |
| ENSMUSG00000074444 | Gm15284 | **91.28** | 1.14E-08 | **Inf** | 3.18E-11 | **140.81** | 1.94E-09 |
| ENSMUSG00000074578 | 1500012F01Rik | **4.04** | 8.22E-21 | **3.99** | 1.31E-21 | **2.67** | 1.18E-11 |
| ENSMUSG00000074766 | Ism1 | **56.07** | 1.62E-11 | **78.47** | 1.32E-13 | **17.90** | 1.53E-07 |
| ENSMUSG00000074934 | Grem1 | **36.14** | 2.02E-26 | **13.27** | 5.03E-18 | **8.37** | 1.58E-13 |
| ENSMUSG00000075304 | Sp5 | **12.69** | 1.43E-16 | **206.30** | 3.92E-51 | **19.01** | 3.11E-22 |
| ENSMUSG00000075543 | Prhoxnb | **19.01** | 1.95E-12 | **176.51** | 1.55E-21 | **9.39** | 3.84E-09 |
| ENSMUSG00000075589 | Gm11536 | **22.38** | 9.91E-16 | **10.07** | 1.09E-12 | **2.88** | 0.001162807 |
| ENSMUSG00000076434 | Wfdc3 | **11.78** | 0.000615313 | **10.13** | 0.00086929 | **12.40** | 0.000554267 |
| ENSMUSG00000076441 | Ass1 | **2.21** | 0.000829952 | **2.35** | 8.46E-05 | **2.56** | 2.31E-05 |
| ENSMUSG00000078202 | Nrarp | **8.35** | 1.69E-05 | **12.13** | 2.73E-07 | **9.83** | 2.53E-06 |
| ENSMUSG00000078706 | Gm53 | **38.05** | 3.01E-07 | **92.67** | 5.77E-10 | **14.08** | 9.50E-05 |
| ENSMUSG00000078922 | Tgtp1 | **26.08** | 9.39E-06 | **11.61** | 0.000226963 | **8.76** | 0.001370931 |
| ENSMUSG00000079681 | Zglp1 | **3.05** | 0.000693532 | **2.56** | 0.007969881 | **3.25** | 0.000759423 |
| ENSMUSG00000081037 | Gm13929 | **Inf** | 4.07E-14 | **4.94** | 6.09E-06 | **2.68** | 0.011977161 |
| ENSMUSG00000081664 | Gm15544 | **4.25** | 7.99E-15 | **2.29** | 1.83E-06 | **2.05** | 5.71E-05 |
| ENSMUSG00000081683 | Fzd10 | **18.37** | 1.33E-11 | **13.28** | 2.09E-10 | **4.14** | 0.000529779 |
| ENSMUSG00000084822 | Gm11768 | **151.10** | 5.26E-07 | **414.95** | 1.35E-08 | **77.44** | 1.70E-06 |
| ENSMUSG00000085015 | Gm14424 | **20.57** | 7.65E-12 | **4.35** | 3.08E-05 | **3.27** | 0.001554276 |
| ENSMUSG00000085071 | Gm14066 | **56.24** | 6.92E-11 | **34.68** | 2.37E-10 | **15.05** | 6.10E-07 |
| ENSMUSG00000085463 | 9430024E24Rik | **79.68** | 2.16E-06 | **471.12** | 2.22E-08 | **173.18** | 2.41E-07 |
| ENSMUSG00000085568 | Gm14858 | **19.85** | 0.015739196 | **44.64** | 0.006432244 | **16.84** | 0.029498498 |
| ENSMUSG00000085696 | 2700086A05Rik | **28.66** | 5.08E-13 | **41.73** | 3.40E-16 | **3.72** | 0.003022421 |
| ENSMUSG00000085715 | Tsix | **7.38** | 0.000199369 | **2.88** | 0.028011232 | **4.29** | 0.004930489 |
| ENSMUSG00000085873 | Gm12750 | **9.23** | 8.24E-10 | **3.14** | 0.001066913 | **3.12** | 0.002123643 |
| ENSMUSG00000086126 | 5730457N03Rik | **676.36** | 1.71E-14 | **2537.62** | 9.42E-18 | **714.65** | 8.42E-16 |
| ENSMUSG00000086290 | Snhg12 | **2.20** | 2.74E-07 | **2.82** | 4.26E-12 | **2.28** | 4.21E-08 |
| ENSMUSG00000086559 | Gm14426 | **38.77** | 8.50E-05 | **19.36** | 0.000121204 | **6.32** | 0.011443772 |
| ENSMUSG00000086711 | Gm15482 | **17.94** | 0.000537827 | **35.94** | 2.45E-05 | **10.34** | 0.002340272 |
| ENSMUSG00000086841 | 2410006H16Rik | **5.11** | 1.08E-20 | **3.60** | 1.49E-14 | **2.05** | 4.20E-05 |
| ENSMUSG00000087028 | Gm13387 | **12.71** | 6.57E-10 | **13.60** | 4.01E-11 | **6.43** | 7.16E-07 |
| ENSMUSG00000090164 | BC035044 | **42.98** | 5.52E-05 | **33.02** | 2.45E-05 | **9.58** | 0.002754332 |
| ENSMUSG00000090665 | Gad1-ps | **6.94** | 0.001916466 | **Inf** | 1.46E-07 | **22.59** | 3.75E-05 |
| ENSMUSG00000092203 | 1110038B12Rik | **2.85** | 2.03E-12 | **2.89** | 2.99E-13 | **2.27** | 2.40E-08 |
| ENSMUSG00000092569 | Gm20544 | **3.08** | 0.00311227 | **3.87** | 0.000320946 | **3.67** | 0.000528739 |
| ENSMUSG00000093470 | Gm20645 | **4.07** | 2.25E-09 | **6.26** | 5.71E-15 | **3.29** | 3.23E-07 |
| ENSMUSG00000094786 | Gm14403 | **3.98** | 1.96E-13 | **2.75** | 1.09E-08 | **2.08** | 8.62E-05 |
| ENSMUSG00000095407 | Tmem200c | **3.24** | 0.002815135 | **3.63** | 0.001462839 | **9.04** | 8.76E-08 |
| ENSMUSG00000096107 | Gm16505 | **25.56** | 3.09E-14 | **23.68** | 1.04E-15 | **9.28** | 1.31E-09 |
| ENSMUSG00000096221 | 1500002C15Rik | **5.99** | 2.49E-14 | **2.79** | 6.28E-07 | **2.09** | 0.000768084 |
| ENSMUSG00000096225 | Lhx8 | **3.46** | 0.029840612 | **7.72** | 0.000164372 | **8.78** | 0.000256309 |
| ENSMUSG00000096847 | Tmem151b | **2.55** | 6.38E-07 | **3.24** | 4.22E-12 | **2.78** | 6.11E-09 |
| ENSMUSG00000096952 | RP23-155H5.3 | **5.28** | 4.14E-07 | **2.57** | 0.002396704 | **2.70** | 0.001536412 |
| ENSMUSG00000097072 | AC120148.1 | **3.67** | 0.012764928 | **8.02** | 1.46E-05 | **3.00** | 0.037142021 |
| ENSMUSG00000097140 | RP24-267K12.1 | **3.67** | 0.000784536 | **2.34** | 0.045692206 | **2.55** | 0.031043312 |
| ENSMUSG00000097154 | CT009715.1 | **19.67** | 0.001136894 | **10.85** | 0.000868655 | **11.68** | 0.00136753 |
| ENSMUSG00000097188 | AC025794.1 | **3.02** | 1.40E-14 | **2.93** | 1.44E-13 | **2.08** | 5.21E-07 |
| ENSMUSG00000097195 | Snhg5 | **2.98** | 2.75E-14 | **3.23** | 4.60E-16 | **2.44** | 7.87E-10 |
| ENSMUSG00000097303 | AC125399.1 | **4.23** | 1.92E-05 | **2.95** | 0.000287026 | **3.20** | 0.000329278 |
| ENSMUSG00000097384 | RP24-67K5.1 | **8.50** | 8.80E-05 | **31.71** | 3.44E-09 | **6.72** | 0.000192926 |
| ENSMUSG00000097386 | 9130213A22Rik | **34.83** | 2.37E-05 | **54.97** | 7.23E-07 | **23.23** | 5.98E-05 |
| ENSMUSG00000097434 | AC162464.1 | **10.71** | 0.002934156 | **8.15** | 0.002437213 | **4.12** | 0.046616395 |
| ENSMUSG00000097796 | AC164431.1 | **9.39** | 0.000169751 | **8.81** | 6.04E-05 | **8.51** | 0.000159414 |
| ENSMUSG00000097917 | AC133083.1 | **7.42** | 0.002880521 | **6.42** | 0.003725003 | **14.34** | 0.000315152 |
